# Serum biomarkers for the diagnosis and management of adolescent polyendocrine metabolic ovarian syndrome: a systematic review and meta-analysis

**DOI:** 10.1016/j.ebiom.2026.106471

**Published:** 2026-09-24

**Authors:** Yue Shi, Chi Chiu Wang, Elisabet Stener-Victorin, Pui Wah Chung, Yao Wang

**Affiliations:** a Department of Obstetrics and Gynaecology, Faculty of Medicine, The Chinese University of Hong Kong, Hong Kong Special Administrative Region of China; b Li Ka Shing Institute of Health Sciences, The Chinese University of Hong Kong, Hong Kong Special Administrative Region of China; c Department of Physiology and Pharmacology, Karolinska Institute, Stockholm, Sweden

**Keywords:** Adolescent, Polyendocrine metabolic ovarian syndrome (PMOS), Polycystic ovary syndrome (PCOS), Biomarker, Diagnostic performance, Risk stratification, Meta-analysis

## Abstract

**Background:**

Adolescent polyendocrine metabolic ovarian syndrome (PMOS) poses immediate and lifelong risks to women’s health. However, reliable biomarkers for adolescent PMOS remain elusive in current clinical practice.

**Methods:**

We systematically searched six databases from inception to April 2026, following PRISMA guidelines (PROSPERO No.: CRD420251033057). Eligible studies included adolescents with PMOS and healthy controls. Primary outcomes were effect sizes of serum reproductive hormones, insulin indices, and adipokines (Hedges’ g in random-effects models), with diagnostic performance assessed by AUC, sensitivity, and specificity.

**Findings:**

Fifty-one moderate-to-high quality studies comprising 4892 adolescents were included. Large effect sizes were observed for total testosterone (TT, 1.10), free androgen index (FAI, 1.19), ratio of luteinising hormone to follicle-stimulating hormone (LH/FSH, 1.07), anti-Müllerian hormone (AMH, 0.98), and adiponectin (-1.12), all with moderate-to-low certainty. Insulin resistance indices (homoeostatic model assessment of insulin resistance [HOMA-IR], 0.64; quantitative insulin sensitivity check index [QUICKI], -0.47) and leptin (0.40) showed small effect sizes with low certainty. In adolescents with obesity, AMH (1.39) and adiponectin (-1.54) showed pronounced effects. For diagnostic performance, TT (cutoff = 55.96 ng/dL), FAI (cutoff = 6.78), and LH/FSH (cutoff = 1.59) each achieved an AUC of 0.74. AMH effectively identified PMOS in obese group (AUC = 0.85, cutoff = 4.47 ng/mL) and the <17-year-old group (AUC = 0.77, cufoff = 6.05 ng/mL). For metabolic stratification, HOMA-IR performed modestly overall AUC of 0.65 (cutoff = 4.35), while adiponectin (AUC = 0.78, cutoff = 10.92 μg/mL) and leptin (AUC = 0.67, cutoff = 36.17 ng/mL) emerged as BMI-dependent markers in obese and non-obese groups, respectively. Of note, LH/FSH and dehydroepiandrosterone sulphate (DHEAS) showed significant publication bias; thus, these results should be interpreted with caution.

**Interpretation:**

TT and FAI are primary diagnostic biomarkers in adolescent PMOS, with LH/FSH providing supportive value. AMH is discriminative in adolescents with obesity in early-mid adolescence. HOMA-IR is a primary biomarker for metabolic monitoring, while adiponectin and leptin are BMI-dependent markers for early metabolic risk stratification.

**Funding:**

This study was supported by HMRF, 10.13039/501100004853CUHK direct grant, CUHK-Chulalongkorn University Research Fund, 1 + 1 + 1 CUHK-CUHK(SZ)-GDST Joint Collaboration Fund, and PhD International Mobility for Partnerships and Collaboration Award.

**Research in context**

**Evidence before this study**

Polycystic ovary syndrome (PMOS), previously known as polyendocrine metabolic ovarian syndrome (PCOS), is the most common gynaecological endocrinopathy in adolescents and is associated with immediate and long-term reproductive and metabolic health risks. According to the 2025 International evidence-based recommendations, adolescent PMOS should be diagnosed based on the coexistence of irregular menstrual cycles and hyperandrogenism. However, adolescent-specific biochemical thresholds remain poorly defined, and informative circulating biomarkers for adolescent PMOS diagnosis remain elusive. Importantly, obesity and pubertal maturation status substantially affect reproductive and metabolic profiles in adolescent PMOS. Together, these challenges highlight the need for biomarker-guided diagnostic and metabolic assessment and evaluation of subgroup-specific performance according to BMI and pubertal stage. Therefore, to evaluate the diagnostic performance of potential biomarkers in adolescent PMOS, we conducted a systematic search of PubMed, Embase, Web of Science, Cochrane Library, Scopus, and ScienceDirect from their inception to April 2026, using the terms “adolescent, teenager, girl” AND “polycystic ovary syndrome, PCOS” AND “reproductive hormones, insulin resistance, adipokine”. PMOS was diagnosed by National Institutes of Health (NIH) criteria or the 2003 Rotterdam criteria, while controls had no PMOS features. 51 studies were included in the synthesis, with 36 of moderate quality and 15 of high quality.

**Added value of this study**

This systematic review and meta-analysis provides a comprehensive quantitative evaluation of potential biomarkers in adolescent PMOS and further estimates their diagnostic performance and thresholds acrossing BMI, age, and diagnostic criteria. Total testosterone (TT) and free androgen index (FAI) are the primary biomarkers for diagnosis in overall adolescent, with the ratio of luteinising hormone to follicle-stimulating hormone (LH/FSH) providing complementary diagnostic value. Notably, anti-Müllerian hormone (AMH) showed discrimination in adolescents with obesity during early-to-mid adolescence. Homoeostatic model assessment of insulin resistance (HOMA-IR) remains the primary index for metabolic risk assessment, whereas quantitative insulin sensitivity check index (QUICKI), adiponectin, and leptin may facilitate phenotype-specific metabolic risk assessment.

**Implications of all the available evidence**

The available evidence supports a stratified biomarker framework for the diagnosis and assessment of adolescent PMOS in a pubertal stage- and BMI-dependent manner. Our findings provide quantitative evidence for biomarker selection, threshold estimation, and diagnostic performance to inform biomarker-guided individualised clinical evaluation in adolescent PMOS. Further multiethnic, large-scale clinical validation of adolescent-specific biomarker thresholds is warranted before routine clinical implementation.

## Introduction

Polyendocrine metabolic ovarian syndrome (PMOS), previously known as polycystic ovary syndrome (PCOS), is the most common gynaecological endocrinopathy, affecting 8%–13% of reproductive-age women.^1^^,^^2^ In adults, it is characterised by menstrual irregularities, hyperandrogenism, and polycystic ovarian morphology (PCOM).^3^ Moreover, the recent renaming from PCOS to PMOS emphasises not only reproductive endocrine features but also metabolic dysfunction. Accordingly, elevations in total testosterone (TT) and dehydroepiandrosterone sulphate (DHEAS) are indicators of biochemical hyperandrogenism.^4^ Increased luteinising hormone (LH) and anti-Müllerian hormone (AMH) levels and insufficient follicle-stimulating hormone (FSH) reflect ovarian dysfunction.^5^^,^^6^ In parallel, PMOS is commonly accompanied by insulin resistance, as evidenced by hyperinsulinemia and adipokine abnormalities.^7^, ^8^, ^9^

Growing evidence suggests that the pathophysiology of PMOS may originate as early as peripuberty and become clinically apparent during adolescence. Adolescent PMOS has an estimated prevalence of 6.3%–9.8% among adolescent females across diagnostic criteria,^10^ with an increased global burden over the past three decades.^11^ Of note, 50–60% of adolescents with PMOS will eventually develop PMOS and retain PMOS-related phenotypes in adulthood.^12^^,^^13^ It increases the long-term risk of a broad spectrum of metabolic sequelae, including hypertension, type 2 diabetes, and metabolic dysfunction-associated steatotic liver disease (MASLD).^14^, ^15^, ^16^, ^17^ Importantly, it further impairs fertility capacity and is associated with an augmented risk of adverse pregnancy outcomes and gynaecological cancer in adulthood.^15^ Therefore, timely detection of adolescent PMOS is imperative to improve women’s lifelong metabolic and reproductive health.

The 2025 International Recommendations define adolescent PMOS by menstrual irregularities and biochemical hyperandrogenism.^9^ However, current diagnosis remains challenging due to the application of adult criteria to adolescents. This may lead to diagnostic uncertainty because several features of adult PMOS largely overlap with natural pubertal physiology, such as early postmenarcheal menstrual irregularities, mild acne, elevated LH and androgen levels, and PCOM.^12^^,^^18^ Moreover, adolescence is accompanied by remarkable reproductive and metabolic remodelling, including a surge in sex steroids, LH, and AMH, and a declined insulin sensitivity until late puberty.^19^^,^^20^ Additionally, nearly half of adolescent PMOS has been conceptualised as a post-pubertal central obesity syndrome.^21^ Considering the strong effects of pubertal transition and obesity on reproductive and metabolic hormone profiles,^22^, ^23^, ^24^ biomarker thresholds should be interpreted within age- and body weight-specific contexts.

Herein, we conducted this systematic review and meta-analysis to comprehensively synthesise current evidence on biomarkers for adolescent PMOS diagnosis, including reproductive hormones, insulin resistance indices, and adipokine levels, stratified by obesity, age, and diagnostic criteria. We aimed to assess their effect sizes and diagnostic performance in overall adolescents and each subgroup, and to estimate thresholds to inform the diagnosis and management of adolescent PMOS.

## Methods

### Search strategy

We systematically searched six electronic databases (PubMed, Embase, Web of Science, Cochrane Library, Scopus, and ScienceDirect) from their inception to April 2026 for evidence on hormone parameters, insulin resistance indices, and adipokines in adolescent PMOS. Different search terms were used for each database across five main contexts (adolescent, PCOS, reproductive hormone, insulin resistance, adipokine). Details of the search strategies are reported in the Supplementary Table S1.

### Study selection and data abstraction

Two review authors (Y.S. and C.C.W.) independently screened the articles, and extracted data on (1) publication characteristics of the publication (author, year, region); (2) participant characteristics of participants (number, age, body mass index (BMI)); (3) diagnostic criteria; and (4) each outcome (mean, standard deviation (SD)). Adolescence was defined as mean age 10–19 years old.^25^ Adolescent PMOS was diagnosed according to National Institutes of Health (NIH) criteria or 2003 Rotterdam criteria.^26^^,^^27^ Healthy controls included adolescents with regular menstruation, no sign of clinical or biochemical hyperandrogenism, and no other features of PMOS (e.g., PCOM, insulin resistance, etc.). To determine basal reproductive hormone levels, fasting blood samples were collected during the follicular phase in the healthy control group and in the adolescent PMOS group with distinguishable menstrual cycles, and on the day of the clinical visit in the adolescent PMOS group with completely irregular cycles or amenorrhoea. Primary outcomes were the effect sizes of clinical hormone parameters (TT, free androgen index [FAI], LH/FSH, DHEAS, and AMH), clinical insulin resistance parameters (homoeostatic model assessment of insulin resistance [HOMA-IR], quantitative insulin sensitivity check index [QUICKI]), and adipokines (adiponectin and leptin) between adolescents with and without PMOS. Secondary outcomes were the optimal cutoff value, area under the receiver operating characteristic curve (AUC), sensitivity, and specificity of the biomarkers for adolescent PMOS diagnosis. We discussed four cases involving diagnostic criteria with a third review author (Y.W.) to resolve discrepancies, contacted study authors of the trials to request clarification for incomplete data, and grouped the publications together for multiple publications from a single trial. Data were extracted using Excel (Microsoft Corp). The review protocol was registered on PROSPERO under registration number CRD420251033057.

### Effect size estimation

Random-effects analyses were used in this meta-analysis. Effect size was calculated as Hedges’ g using the R package *meta* (v8.2.1)^28^; Hedges’ g is a variant of Cohen’s d that corrects for bias due to sample size and is interpreted as follows: small effect (g = 0.2), medium effect (g = 0.5), and large effect (g = 0.8).^29^

### Diagnostic performance estimation

To estimate the diagnostic performance, AUC was estimated as Φ((μ_1_ − μ_0_)/sqrt(σ_1_^2^ + σ_0_^2^)), where Φ denotes the cumulative distribution function; μ_1_, σ_1_ denote the pooled mean and standard deviation (SD) of the adolescent PMOS group; and μ_0_, σ_0_ denote the pooled mean and SD of the healthy group.^30^^,^^31^ If the level of a parameter was higher in the adolescent PMOS group, under a given cutoff value (c), sensitivity was calculated as P(X > c | adolescent PMOS) = 1 − *p*_norm_(c, mean = μ_1_, SD = σ_1_), while specificity was calculated as P(X ≤ c | HC) = *p*_norm_(c, mean = μ_0_, SD = σ_0_).^32^^,^^33^ If the level of a parameter was lower in the adolescent PMOS group, sensitivity was calculated as P(X < c | adolescent PMOS) = *p*_norm_(c, mean = μ_1_, SD = σ_1_), while specificity was calculated as P(X ≥ c | HC) = 1 − *p*_norm_(c, mean = μ_0_, SD = σ_0_).^32^^,^^33^ The Youden index was defined as sensitivity + specificity - 1.^34^ The cutoff value was determined at the maximum Youden index.^34^ The receiver operating characteristic (ROC) curve for each parameter or biomarker was generated from a series of cutoff values estimated using the R packages *stats* and *pROC*.^33^ The maximum Youden index was identified using the optimise function in R software (v4.1.0).^35^

### Risk of bias assessment

The methodological quality of the studies included studies was assessed by two review authors (Y.S. and C.C.W.) using an 11-item checklist which was recommended by the Agency for Healthcare Research and Quality (AHRQ) for cross-sectional studies by two review authors (Y.S. & C.C.W).^36^ An item was scored ‘0’ if it was answered ‘NO’ or ‘UNCLEAR’; if it was answered ‘YES’, the item was scored ‘1’. Article quality was classified as follows: low quality = 0–3; moderate quality = 4–7; high quality = 8–11.^36^ We discussed discrepancies with a third review author (Y.W.) to reach consensus.

Publication bias was assessed using Egger’s regression test and visual funnel plot symmetry with the R package *meta* (v8.2.1).^28^ A *p*-value < 0.05 for Egger’s test and an asymmetric funnel plot indicated the presence of publication bias, and post hoc trim-and-fill analyses were conducted to account for publication bias. The absolute shift and relative percentage shift between the original and adjusted pooled effect sizes were computed to quantify the impact of publication bias.^37^

### Heterogeneity and sensitivity assessment

Between-study heterogeneity was assessed using the Cochran *Q* statistic (*p* < 0.05 indicated heterogeneity), between-study variance was assessed using *τ*^2^, and the magnitude of between-study variation attributable to true differences in effect sizes rather than chance was assessed using *I*^2^ (*I*^2^ ≥ 50% was considered substantial heterogeneity) via the R package *meta* (v8.2.1).^28^^,^^38^ When substantial heterogeneity was observed, we conducted subgroup analyses to investigate potential explanations using the R package *meta* (v8.2.1).^28^^,^^39^ Subgroup analyses were conducted for: (1) obesity status (obese group: mean BMI > 30, or mean BMI *z*-score > 1.96, or mean BMI standard deviation score (SDS) > 2; non-obese group: mean BMI ≤ 30, or mean BMI *z*-score ≤ 1.96, or mean SDS ≤ 2)^40^; (2) puberty status (early-mid adolescence group: <17 years old; late adolescence group: ≥17 years old)^41^; and (3) diagnostic criteria (NIH criteria group: PMOS is diagnosed by the presence of both two (a) oligomenorrhea or amenorrhoea and (b) clinical or laboratory evidence of hyperandrogenemia, after the exclusion of related disorders^26^; 2-in-3 Rotterdam criteria group: PMOS is diagnosed by the presence of two of the following three features: (a) oligo- or anovulation, (b) clinical and/or biochemical signs of hyperandrogenism, and (c) polycystic ovaries, after the exclusion of related disorders^27^; 3-in-3 Rotterdam criteria group: PMOS is diagnosed by the presence of all the three aforementioned features).^27^

To further explore heterogeneity within subgroups, we performed subgroup-stratified univariate meta-regression using the R package *metafor* (v4.6.0).^42^ Parameters containing more than 10 studies were analysed to ensure adequate statistical power for stable estimation of subgroup-specific random-effects variance and reliable detection of potential effect modifiers. Moderator analyses were conducted using random-effects models. The significance of each covariate was evaluated using the omnibus test of moderators based on a Wald-type test, and the proportion of between-study variance was quantified by R^2^.^43^

Moreover, sensitivity analyses were conducted using a random-effects models with the R package *meta* (v8.2.1).^28^^,^^44^ We first applied the leave-one-out method to assess the leave-one-out method to assess the influence of individual studies on the pooled effect estimates. In addition, group-omission sensitivity analysis was conducted to assess the potential impact of study quality on pooled estimates by omitting all low- to moderate-quality studies.

### Evidence certainty assessment

Evidence certainty assessment was conducted following Grading of Recommendation Assessment, Development and Evaluation (GRADE) working group approach.^45^ Given that all included studies were observational studies, the baseline certainty of evidence started from a ‘LOW’ level. The rating was upgraded for a large effect size, assuming no major residual confounding would explain the observed effect. Conversely, evidence was downgraded for serious limitations in risk of bias, indirectness of evidence, inconsistency across studies, or imprecision of results. Summary of findings tables were created using the GRADEpro Guideline Development Tool (https://gdt.gradepro.org/).

### Role of the funding source

The funders of the study had no role in study design, data collection, data analysis, data interpretation, or writing of the report.

## Results

### Trial flow and quality assessment

This study was conducted followed PRISMA reporting guidelines.^46^ A total of 10,926 studies were initially identified. After screening and eligibility assessment, studies were excluded without full text or lacked targeted populations, criteria, or biomarkers (Supplementary Table S2). 51 studies were included in the meta-analysis (Fig. 1), comprising 4892 adolescents aged from 12 to 19 years old, with 2388 PMOS cases and 2504 healthy controls (Table 1).

**Fig. 1:**
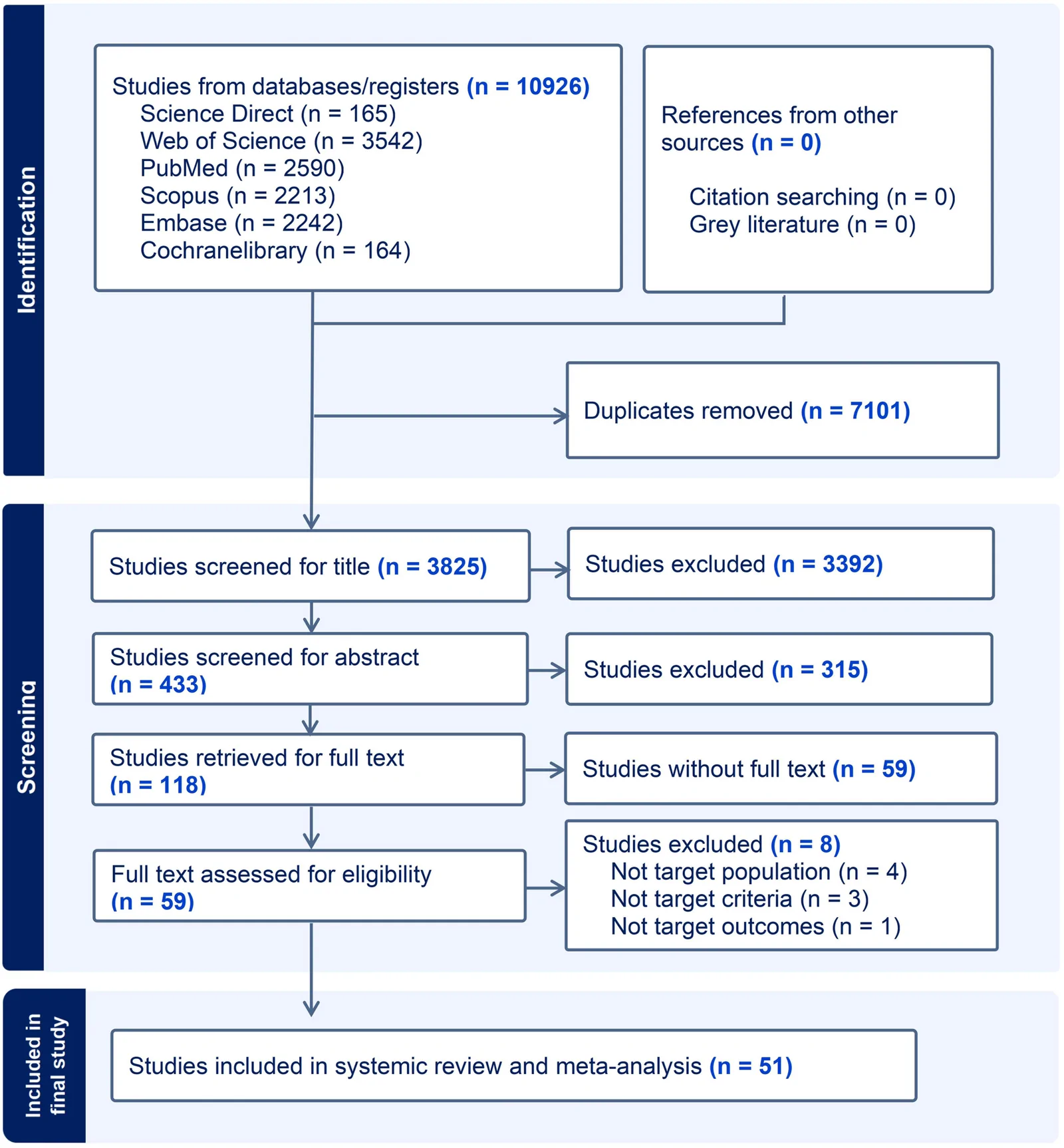
**Study selection process.** We systematically searched six electronic databases (PubMed, Embase, Web of Science, Cochrane Library, Scopus, and ScienceDirect) from their inception to April, 2026. A total of 10,926 studies were initially identified as likely meeting the inclusion criteria, and evaluation of these studies resulted in 51 studies included in the meta-analysis.

**Table 1:** Characteristics of the studies included in the synthesis.

Author	Region	Year	No. (PMOS)	No. (HC)	Age (PMOS)	Age (HC)	BMI (PMOS)	BMI (HC)	Criteria	Parameters
J. D. Veldhuis	USA	2001	11	9	16.9 ± 0.48	16.7 ± 0.53	22.6 ± 1.06	22.0 ± 1.12	NIH	DHEAS, Leptin
Vered D. Lewy	USA	2001	12	10	12.0 ± 0.7	12.1 ± 0.6	33.1 ± 1.8	31.4 ± 1.3	NIH	TT, Leptin
Miriam E. Silfen	USA	2003	22	15	15.5 ± 1.4	14.4 ± 1.5	35.9 ± 6.2	35.8 ± 7.1	NIH	TT, DHEAS, QUICKI
Fatma Demirel (1)	Turkey	2007	23	31	15.1 ± 1.3	15.5 ± 1.3	21.6 ± 2.4	29.6 ± 2.1	NIH	LH/FSH, DHEAS, HOMA-IR, Leptin
Fatma Demirel (2)	Turkey	2007	23	31	15.5 ± 0.9	15.5 ± 1.3	21.6 ± 2.4	21.1 ± 2.1	NIH	LH/FSH, DHEAS, HOMA-IR, Leptin
Aysun Bideci	Turkey	2008	16	19	15.2 ± 0.8	14.8 ± 1.0	30.4 ± 3.4	30.3 ± 2.9	Rotterdam	DHEAS, LH/FSH, Leptin, HOMA-IR
Brooke Rossi	USA	2008	43	31	15.6 ± 1.5	14.8 ± 1.8	2.18 ± 0.38 (z-score)	2.18 ± 0.39 (z-score)	NIH	TT, FAI
Franca Fruzzetti (1)	Italy	2009	120	30	12–19	13–19	24.5 ± 4.9	23.5 ± 2.3	NIH	TT, FAI, HOMA-IR
Franca Fruzzetti (2)	Italy	2009	53	30	17.2 ± 1.8	17.4 ± 1.6	25.1 ± 5.3	23.1 ± 4.1	NIH	TT, FAI, HOMA-IR
Orit Pinhas-Hamiel (1), obese cohort	Israel	2009	14	14	15.2 ± 1.2	14.2 ± 2.1	29.6 ± 13.2	18.6 ± 1.7	Rotterdam	TT, DHEAS, AMH, Adiponectin, Leptin
Orit Pinhas-Hamiel (2), non-obese cohort	Israel	2009	16	14	15.5 ± 1.7	14.2 ± 2.1	20.1 ± 2	18.6 ± 1.7	Rotterdam	TT, DHEAS, AMH, Adiponectin, Leptin
Ayla Güven (1), obese cohort	Turkey	2010	10	7	14.7 ± 0.9	15.1 ± 1	34.2 ± 5	29.7 ± 4	Rotterdam	TT, DHEAS, LH/FSH, HOMA-IR, QUICKI, Adiponectin
Ayla Güven (2), non-obese cohort	Turkey	2010	12	9	15.7 ± 1	15.1 ± 0.8	20.1 ± 1	20.1 ± 1	Rotterdam	TT, DHEAS, LH/FSH, HOMA-IR, QUICKI, Adiponectin
Gideon de Sousa	Germany	2010	31	19	15.0 ± 1.0	15.2 ± 1.1	32.7 ± 6.2	32.4 ± 4.0	NIH	TT, HOMA-IR, QUICKI
L. Yasar (1), non-obese cohort	Turkey	2010	20	21	17.45 ± 0.99	17.19 ± 0.40	21.43 ± 2.07	18.64 ± 1.58	Rotterdam	Adiponectin
L. Yasar (2), obese cohort	Turkey	2010	22	23	17.36 ± 0.79	16.91 ± 1.04	29.99 ± 1.97	31.40 ± 5.17	Rotterdam	Adiponectin
Stefano Angioni	Italy	2010	79	50	16.37 ± 3.79	16.87 ± 4.55	20.84 ± 2.19	20.76 ± 2.37	Rotterdam	TT, DHEAS, LH/FSH, QUICKI
Xiaoli Chen	China	2010	19	20	17.89 ± 1.24	18.6 ± 0.68	21.46 ± 4.60	20.11 ± 2.31	Rotterdam	TT, FAI, HOMA-IR
Ferhat Cekmez	Turkey	2011	48	37	16.9 ± 0.3	17.2 ± 0.2	35.1 ± 4.3	30.7 ± 2.2	Rotterdam	TT, FAI, HOMA-IR, Adiponectin
Aviva B Sopher	USA	2012	13	11	16.4 ± 2.1	18.8 ± 2.36	22.4 ± 2.7	22.4 ± 2.5	NIH	TT, DHEAS, HOMA-IR
Haleh Rahmanpour	Iran	2012	30	71	17.73 ± 1.01	17.69 ± 1.29	23.4 ± 3.09	21.06 ± 2.85	NIH	HOMA-IR
Paola Villa	Italy	2012	86	48	15.7 ± 1.4	15.3 ± 1.7	25.7 ± 5.4	23.9 ± 4.9	NIH	TT, FAI, HOMA-IR, QUICKI
Rüya Deveer	Turkey	2012	25	25	18.16 ± 3.07	18.20 ± 2.34	23.32 ± 3.95	21.84 ± 2.87	Rotterdam	HOMA-IR
Andrea Hsu Roe	USA	2013	148	57	16.9 ± 1.9	16.6 ± 2.5	28.5 ± 7.4	24.7 ± 7.1	Rotterdam	DHEAS
Tμgba Kale-Gurbuz	Turkey	2013	17	21	18.17 ± 1.62	17.52 ± 1.16	30.80 ± 4.82	30.77 ± 2.86	Rotterdam	TT, DHEAS, LH/FSH, HOMA-IR, Adiponectin, Leptin
Aviva B Sopher	USA	2014	15	16	16.6 ± 2.1	18.6 ± 2.6	nonobese	nonobese	NIH	TT, DHEAS, AMH
Melissa Pawelczak	USA	2014	19	7	15.2 ± 1.84	14.08 ± 1.7	2.07 ± 0.48 (z-score)	2.04 ± 0.25 (z-score)	Rotterdam	HOMA-IR
Sevil Cankaya	Turkey	2014	39	40	17.79 ± 1.59	17.43 ± 1.69	21.51 ± 1.92	21.25 ± 1.75	Rotterdam	TT, DHEA-S, HOMA-IR, Adiponectin
Aytekin Tokmak	Turkey	2015	43	47	18.9 ± 2.2	18.4 ± 2.4	22.9 ± 4.7	21.8 ± 2.8	3/3 of Rotterdam	LH/FSH, AMH, DHEAS, HOMA-IR
Claudio Villarroel	Chile	2015	26	63	17.3 ± 1.9	16.6 ± 1.5	1.0 ± 1.0 (SDS)	0.4 ± 0.8 (SDS)	NIH	LH/FSH, AMH, DHEAS, HOMA-IR
Aylin Yetim	Turkey	2016	53	26	16.72 ± 1.41	15.18 ± 2.00	1.40 (−1.74 to 9.20) (SDS)	0.78 (−1.99 to 3.14) (SDS)	Rotterdam	DHEAS
Kara S Hμghan	USA	2016	91	30	15.8 ± 0.2	14.6 ± 0.3	37.9 ± 0.7	35.3 ± 1.1	NIH	HOMA-IR, Leptin, Adiponectin
Joon Young Kim	USA	2017	46	43	14.9 ± 0.2	14.4 ± 0.2	37.7 ± 1.1	33.1 ± 1.1	NIH	Adiponectin
Melanie Cree-Green	USA	2017	28	20	15.53 ± 1.56	14.97 ± 2.07	1.73 ± 0.96	1.67 ± 0.74	NIH	TT, FAI, AMH, Adiponectin, HOMA-IR, QUICKI
Sonali S Patel	USA	2017	36	17	14.81 ± 1.56	13.94 ± 1.75	35.60 ± 3.66	32.16 ± 2.57	NIH	TT, FAI, Leptin, Adiponectin
Aytekin Tokmak	Turkey	2018	42	47	19.0 ± 2.0	18.0 ± 3.0	23.8 ± 4.6	20.8 ± 2.9	3/3 of Rotterdam	DHEAS, HOMA-IR
Esra Isci Bostanci	Turkey	2018	31	35	18.65 ± 2.4	18.23 ± 2.1	22.64 ± 3.58	22.52 ± 5.06	Rotterdam	HOMA-IR, LH/FSH
Flora Bacopoulou	Greece	2018	23	19	16.9 ± 2.1	17.9 ± 2.2	20.9 ± 1.2	20.3 ± 1.3	Rotterdam	TT, DHEAS, QUICKI
Seda Ates	Turkey	2018	77	33	17.68 ± 1.19	17.94 ± 1.05	24.87 ± 5.03	21.42 ± 2.73	NIH	TT, FAI, LH/FSH, HOMA-IR
Antigoni Manousopoulou	Turkey	2019	20	20	18.2 ± 2.9	17.3 ± 2.1	21.3 ± 1.6	21.0 ± 2.0	Rotterdam	TT, FAI, DHEAS, QUICKI
İlker Tolga Özgen	Turkey	2019	41	30	14.40 ± 1.50	14.39 ± 2.01	30.54 ± 5.13	30.49 ± 4.47	Rotterdam	TT, HOMA-IR
Donna F Vine	Australia	2020	33	151	17.10 ± 0.30	17.03 ± 0.10	25.86 ± 6.02	22.57 ± 3.65	NIH	TT, FAI, DHEAS, HOMA-IR
Elena Khashchenko	Russia	2020	130	30	16.0 (15.0–17.0)	16.0 (15.0–17.0)	22.4 (19.9–27.2)	20.2 (18.4–21.8)	Rotterdam	TT, DHEAS, Leptin
Agnieszka Zachurzok	Poland	2021	27	27	16.7 ± 1.2	16.1 ± 1.1	1.1 ± 0.9	1.0 ± 1.0	NIH	TT, DHEAS
							(z score)	(z score)		
Elif Sağsak	Turkey	2021	20	72	16 ± 1.58	14.9 ± 1.72	25.4 ± 4.96	21 ± 2.82	3/3 of Rotterdam	TT, FAI, HOMA-IR
Elvan Bayramoğlu (1), obese cohort	Turkey	2021	22	21	16.4 ± 1.1	16.26 ± 0.98	31.55 ± 4.2	33.03 ± 3.83	NIH	HOMA-IR
Elvan Bayramoğlu (2), non-obese cohort	Turkey	2021	16	19	16.3 ± 1.06	16.14 ± 1.01	22.05 ± 2.8	21.15 ± 2.08	NIH	HOMA-IR
Selma Tunç	Turkey	2021	55	25	15.54 ± 2.37	15.88 ± 1.34	1.44 ± 0.89 (SDS)	1.34 ± 0.67 (SDS)	Rotterdam	LH/FSH, DHEAS, AMH
Shivi Jain	India	2021	50	50	16.18 + 1.48	16.38 + 1.27	27.10 + 4.55	26.22 + 2.44	Rotterdam	LH/FSH
Merve Gungor	Turkey	2022	38	27	16.3 ± 1.8	16.4 ± 1.5	22.3 ± 3.5	23.4 ± 4.2	NIH	FAI, DHEAS, HOMA-IR
Giannouli, A (1), non-obese cohort	Greece	2023	19	35	15.36 ± 1.56	15.45 ± 1.59	21.27 ± 2.25	23.23 ± 4.19	Rotterdam	TT, DHEAS
Giannouli, A (2), obese cohort	Greece	2023	21	35	15.54 ± 1.65	15.45 ± 1.59	29.64 ± 3.92	23.23 ± 4.19	Rotterdam	TT, DHEAS
Gönül Büyükyılmaz (1), obese cohort	Turkey	2023	35	26	16.2 ± 1.4	15.9 ± 1.3	32.3 ± 3.9	26.7 ± 2.9	NIH	TT, FAI, LH/FSH, AMH
Gönül Büyükyılmaz (2), non-obese cohort	Turkey	2023	21	44	16 ± 1.3	16 ± 1.4	22.3 ± 2.6	20.2 ± 2	NIH	TT, FAI, LH/FSH, AMH
Marta Díaz	Spain	2023	73	28	15.8 ± 0.2	16.3 ± 0.3	23.5 ± 0.4	22.6 ± 0.4	NIH	Adiponectin
Karolina Skrzyńska	Poland	2024	47	33	15.68 ± 1.18	15.79 ± 1.22	26.66 ± 6.41	25.44 ± 7.24	NIH	TT, DHEAS, HOMA-IR
Rachel C Whooten (1), non-obese cohort	USA	2024	50	332	13.3 ± 0.9	13.1 ± 0.8	0.88 ± 1.01 (z score)	0.25 ± 1.08 (z score)	NIH	Leptin, Adiponectin
Rachel C Whooten (2), non-obese cohort	USA	2024	53	310	17.8 ± 0.8	17.7 ± 0.7	0.78 ± 1.03 (z-score)	0.33 ± 0.97 (z score)	NIH	Leptin, Adiponectin
Leyla Kara	Turkey	2025	69	63	15.66 (14.16–16.73)	15.72 (14.16–16.9)	25.95 ± 3.98	25.07 ± 5.25	Rotterdam	FAI, DHEAS
Elzbieta Forys	Poland	2025	66	30	16.60 ± 1.48	16.43 ± 1.11	26.63 ± 8.59	23.87 ± 4.52	Rotterdam	HOMA-IR, QUICKI, Leptin

Criteria: Rotterdam 2003: PMOS is diagnosed by having two of the following three: (1) oligo- or anovulation, (2) clinical and/or biochemical signs of hyperandrogenism, (3) polycystic ovaries, after the exclusion of related disorders. National Institutes of Health (NIH): PMOS is diagnosed by having both two (1) oligomenorrhea or amenorrhoea, (2) clinical or laboratory evidence of hyperandrogenemia, after the exclusion of related disorders.

Total testosterone (TT), free androgen index (FAI), ratio of luteinising hormone to follicle-stimulating hormone (LH/FSH), dehydroepiandrosterone sulphate (DHEAS), anti-Müllerian hormone (AMH), homoeostatic model assessment of insulin resistance (HOMA-IR), quantitative insulin sensitivity check index (QUICKI), polyendocrine metabolic ovarian syndrome (PMOS), healthy control (HC).

Quality assessment identified 36 moderate quality studies and 15 high quality studies (Supplementary Table S3). Among them, 28 studies measured TT,^47^, ^48^, ^49^, ^50^, ^51^, ^52^, ^53^, ^54^, ^55^, ^56^, ^57^, ^58^, ^59^, ^60^, ^61^, ^62^, ^63^, ^64^, ^65^, ^66^, ^67^, ^68^, ^69^, ^70^, ^71^, ^72^, ^73^, ^74^ 14 studies reported FAI,^49^^,^^52^^,^^54^^,^^56^^,^^60^^,^^63^^,^^65^^,^^66^^,^^68^^,^^69^^,^^72^^,^^74^, ^75^, ^76^ 26 studies examined DHEAS,^48^^,^^50^^,^^53^^,^^57^, ^58^, ^59^^,^^61^^,^^62^^,^^64^^,^^66^, ^67^, ^68^^,^^70^^,^^71^^,^^73^^,^^75^, ^76^, ^77^, ^78^, ^79^, ^80^, ^81^, ^82^, ^83^, ^84^, ^85^ 12 studies provided data on LH/FSH,^23^^,^^53^^,^^57^^,^^58^^,^^63^^,^^72^^,^^78^^,^^79^^,^^81^^,^^82^^,^^85^^,^^86^ and 7 studies assayed AMH.^50^^,^^61^^,^^65^^,^^72^^,^^81^^,^^82^^,^^85^ Among metabolic parameters, HOMA-IR was available in 28 studies,^51^, ^52^, ^53^^,^^55^, ^56^, ^57^^,^^59^^,^^60^^,^^62^^,^^63^^,^^68^^,^^69^^,^^73^, ^74^, ^75^^,^^78^^,^^79^^,^^81^^,^^82^^,^^84^^,^^86^, ^87^, ^88^, ^89^, ^90^, ^91^, ^92^ QUICKI in 9 studies,^48^^,^^51^^,^^57^^,^^58^^,^^60^^,^^64^, ^65^, ^66^^,^^92^ adiponectin in 12 studies,^50^^,^^52^, ^53^, ^54^^,^^57^^,^^62^^,^^65^^,^^88^^,^^93^, ^94^, ^95^, ^96^ and leptin in 11 studies.^47^^,^^50^^,^^53^^,^^54^^,^^67^^,^^77^, ^78^, ^79^^,^^88^^,^^92^^,^^96^

For publication bias, the funnel plots indicated that TT, FAI, AMH, HOMA-IR, QUICKI, adiponectin, and leptin appeared roughly symmetrical by visual inspection, and Egger’s test showed *p* values > 0.05; therefore, these results were relatively stable. However, LH/FSH and DHEA-S showed asymmetrical funnel plots and *p*-values < 0.05, indicating a risk of publication bias. The effect sizes were reduced by 33.3% for DHEAS and 18.3% for LH/FSH after trim-and-fill analysis; therefore, these results should be interpreted with caution (Supplementary Fig. S1). In the sensitivity analysis, removing each study had no significant effect on the effect sizes of the biomarkers, and we retained the original results as shown in Supplementary Table S4. However, the effect size of adiponectin was significantly reduced after removing studies of moderate quality, indicating that the pooled result may be influenced by study quality (Supplementary Table S5). In terms of evidence certainty, TT, FAI, AMH, and adiponectin showed moderate certainty, while LH/FSH, HOMA-IR, QUICKI, and leptin showed low certainty. DHEAS was ranked with very low certainty due to significant publication bias and limited effect size (Supplementary Table S6).

### Adolescent PMOS is featured by high TT and FAI in overall population, with increased AMH in obesity and early-mid puberty subgroup

We first examined the effects of reproductive hormones using stratified analyses based on BMI, age and diagnosis criteria. In overall adolescents, TT (Hedges’ g = 1.10, 95% CI: 0.87–1.34, *p* < 0.001), FAI (Hedges’ g = 1.19, 95% CI: 0.93–1.45, *p* < 0.001), LH/FSH (Hedges’ g = 1.07, 95% CI: 0.88–1.27, *p* < 0.001), and AMH (Hedges’ g = 0.98, 95% CI: 0.50–1.46, *p* < 0.001) showed large overall effect sizes, while DHEAS showed a medium effect size (Hedges’ g = 0.64, 95% CI: 0.47–0.81, *p* < 0.001) (Fig. 2). Notably, TT (Hedges’ g = 3.05, 95% CI: 2.38–3.72, *p* < 0.001) and FAI (Hedges’ g = 2.61, 95% CI: 1.98–3.24, *p* < 0.001) showed considerable effect sizes using 3-of-3 Rotterdam criteria. Differences in diagnostic criteria further explained 45.03% and 93.9% of the heterogeneity in TT and FAI, respectively, within the early-mid puberty group (Supplementary Fig. S2A and B and Table S7). By contrast, DHEAS and LH/FSH showed comparable effect sizes across BMI, age, and diagnostic-criteria subgroups (Fig. 2C and D, Supplementary Figs. 2C and D, 3C and D). AMH showed BMI- and age-dependent patterns, with a greater effect size in the adolescents with obesity (Hedges’ g = 1.39, 95% CI: 0.92–1.86, *p* < 0.001) (Fig. 2E) and <17 years old subgroup (Hedges’ g = 1.28, 95% CI: 0.86–1.69, *p* < 0.001) (Supplementary Fig. S3E). Meta-regression demonstrated that age explained 59.1% of AMH heterogeneity within the non-obese group (Supplementary Table S7). Collectively, our data suggested that TT and FAI were more sensitive to diagnostic-criteria differences, whereas AMH showed BMI- and age-dependent differences.

**Fig. 2:**
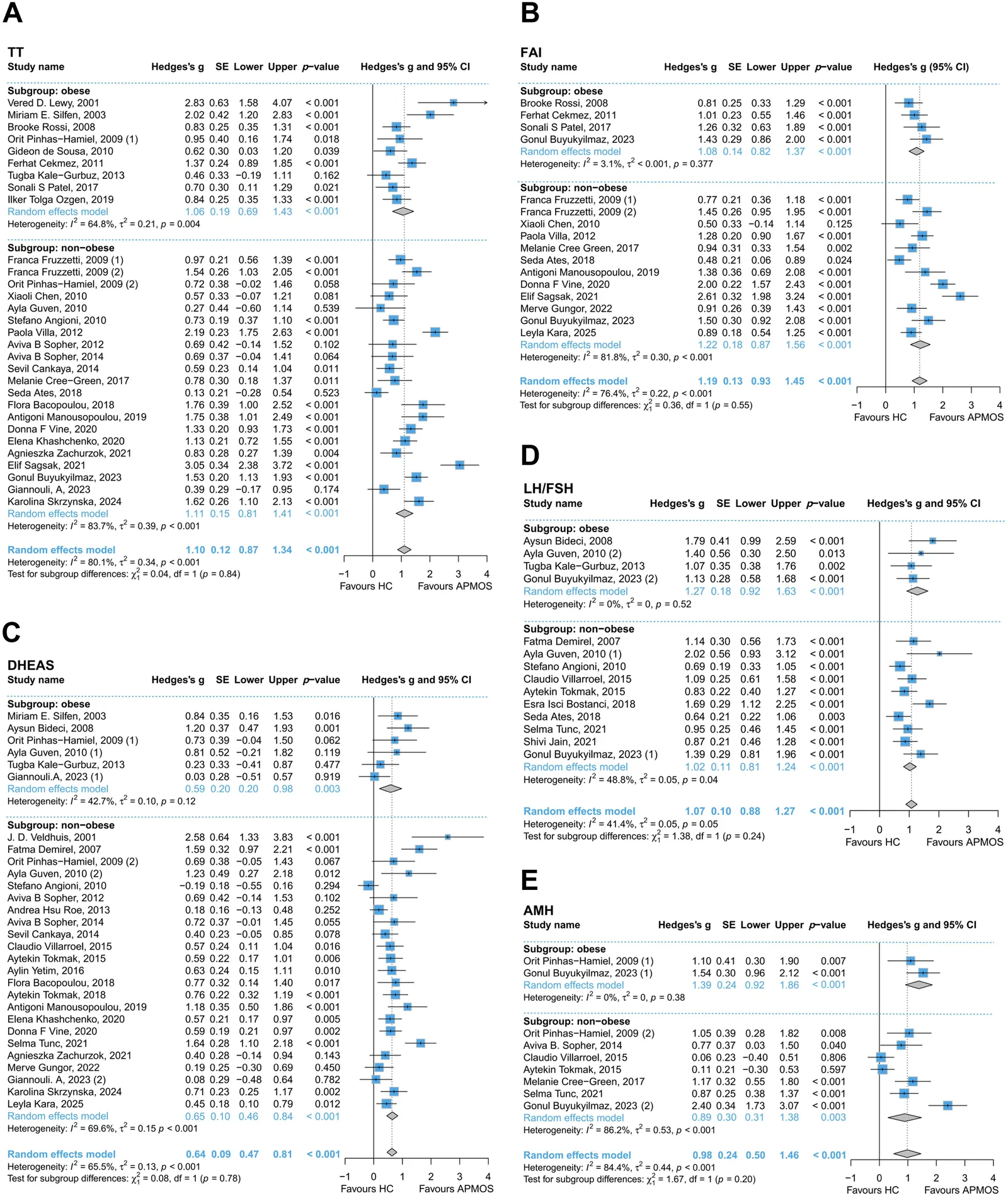
**Effect sizes of the reproductive hormone levels in overall adolescent PMOS and subgroups stratified by BMI (A–E).** Effect size was calculated using Hedges’ g. Among clinical hormone parameters, (**A**) 28 studies examined TT, (**B**) 14 studies examined FAI, (**C**) 26 studies examined DHEAS, (**D**) 12 studies examined LH/FSH, and (**E**) 7 studies examined AMH. Abbreviation: total testosterone (TT), free androgen index (FAI), ratio of luteinising hormone to follicle-stimulating hormone (LH/FSH), dehydroepiandrosterone sulphate (DHEAS), Anti-Müllerian hormone (AMH), polyendocrine metabolic ovarian syndrome (PMOS), healthy control (HC), confidence interval (CI).

### HOMA-IR is consistently elevated in adolescent PMOS, aligned with BMI- and age-dependent alterations in QUICKI and adipokines

Insulin resistance is associated with hyperandrogenism, low-grade inflammation, and metabolic dysfunction in PMOS.^2^ We therefore evaluated insulin resistance indices and adipokines in adolescent PMOS. Compared with healthy controls, adolescents with PMOS had higher HOMA-IR (Hedges’ g = 0.64, 95% CI: 0.44–0.84, *p* < 0.001) and lower QUICKI (Hedges’ g = -0.47, 95% CI: -0.73 to -0.20, *p* < 0.001). For adipokines, adiponectin demonstrated a large difference (Hedges’ g = -1.12, 95% CI: -1.55 to -0.69, *p* < 0.001), while leptin significantly differs between groups with a medium effect size (Hedges’ g = 0.40, 95% CI: 0.09–0.71, *p* < 0.001) (Fig. 3).

**Fig. 3:**
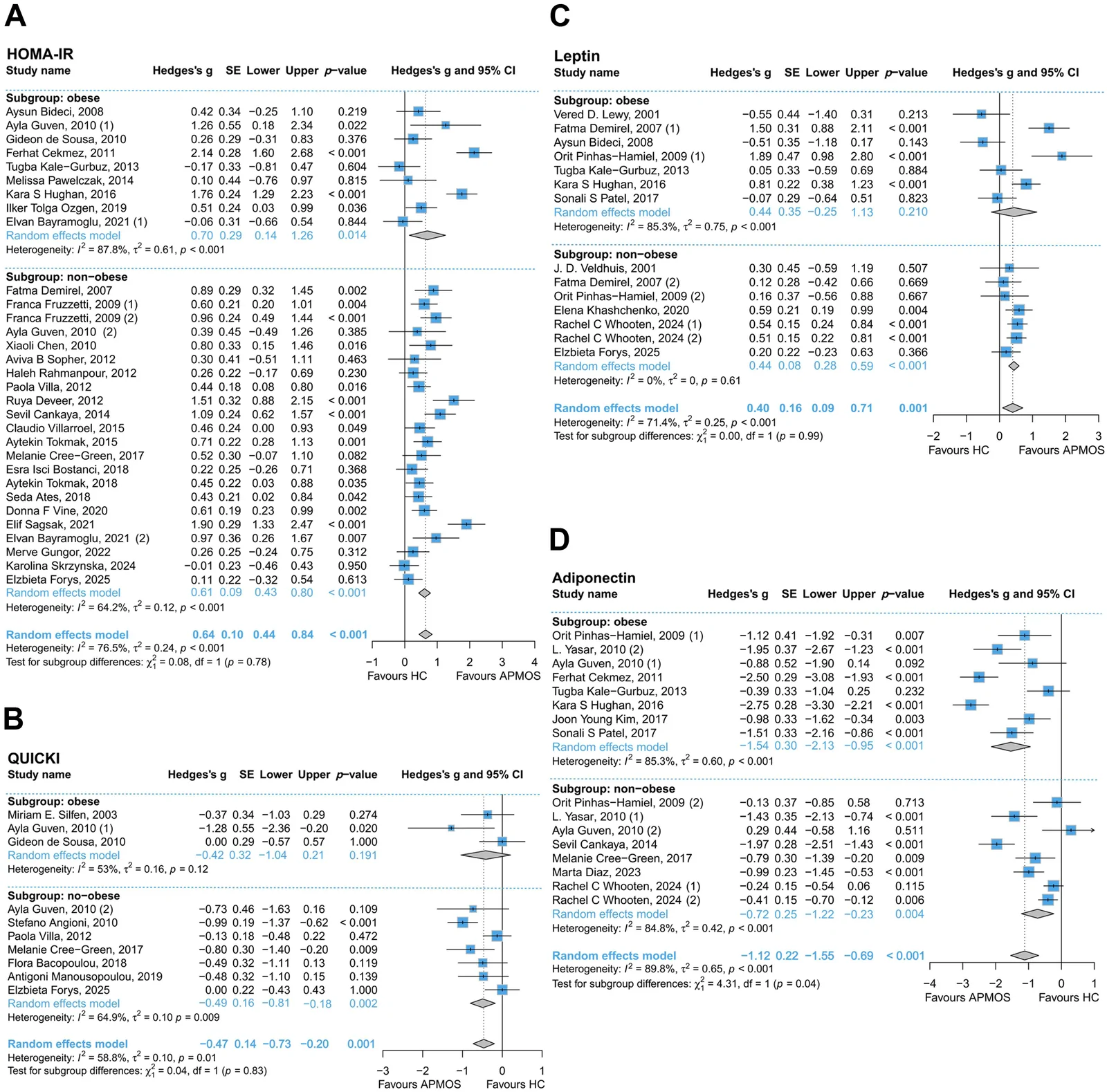
**Effect sizes of the insulin resistance indices and adipokines in overall adolescent PMOS overall and in subgroups stratified by BMI (A–D).** Effect size was calculated using Hedges’ g. Among clinical insulin resistance indices, (**A**) 28 studies examined HOMA-IR and (**B**) 9 studies examined QUICKI. Among the novel adipokine parameters, (**C**) 11 studies examined leptin and (**D**) 12 studies examined adiponectin. Abbreviations: homoeostatic model assessment of insulin resistance (HOMA-IR), quantitative insulin sensitivity check index (QUICKI), polyendocrine metabolic ovarian syndrome (PMOS), healthy control (HC), confidence interval (CI).

Subgroup analysis showed that HOMA-IR was consistently increased in adolescents with PMOS across age, BMI, and diagnostic-criteria strata (Fig. 3A, Supplementary Fig. S4A and 5A). By contrast, QUICKI (Hedges’ g = -0.49, 95% CI: -0.81 to -0.18, *p* = 0.002) and leptin (Hedges’ g = 0.44, 95% CI: 0.28–0.59, *p* < 0.001) were significantly altered only in the non-obese group (Fig. 3B and C), with these differences further restricted to the < 17 years old group (QUICKI: Hedges’ g = -0.47, 95% CI: -0.76 to -0.17, *p* = 0.002; leptin: Hedges’ g = 0.42, 95% CI: 0.05–0.78, *p* < 0.001) (Supplementary Fig. S4B and C). Adiponectin also displayed BMI-dependent patterns with a marked effect size in the obese subgroup (Hedges’ g = -1.54, 95% CI: -2.13 to -0.95, *p* < 0.001) (Fig. 3D), and obesity status explained over half of heterogeneity in adiponectin levels among studies in the <17 years old group or using NIH criteria. In addition, all insulin resistance indices and adipokines presented robust effect sizes across diverse diagnostic criteria (Supplementary Fig. S5 and Table S7). Overall, these findings indicate that HOMA-IR serves as a consistent biomarker for metabolic assessment in adolescent PMOS, while QUICKI, leptin, and adiponectin demonstrate BMI- and age-dependent variation.

### Biomarker assessment for adolescent PMOS diagnosis and metabolic evaluation

We next estimated the performance of these biomarkers for adolescent PMOS diagnosis and metabolic risk stratification. In the overall population, TT (threshold = 55.96 ng/dL), FAI (threshold = 6.78), and LH/FSH (threshold = 1.59) achieved the highest discriminative power, with comparable AUC around 0.74. Moreover, HOMA-IR revealed adolescent PMOS-related insulin resistance (AUC = 0.65, threshold = 4.35), and leptin (AUC = 0.69, threshold = 34.05 ng/mL) may capture adipose-insulin dysregulation. By contrast, DHEAS (AUC = 0.64, threshold = 274.04 μg/dL) and AMH (AUC = 0.65, threshold = 8.32 ng/mL) showed suboptimal diagnostic capability, while QUICKI (AUC = 0.50, threshold = 0.28) and adipokine (AUC = 0.60, threshold = 9.77 μg/mL) are less effective to feature metabolic risk (Fig. 4, Table 2).

**Fig. 4:**
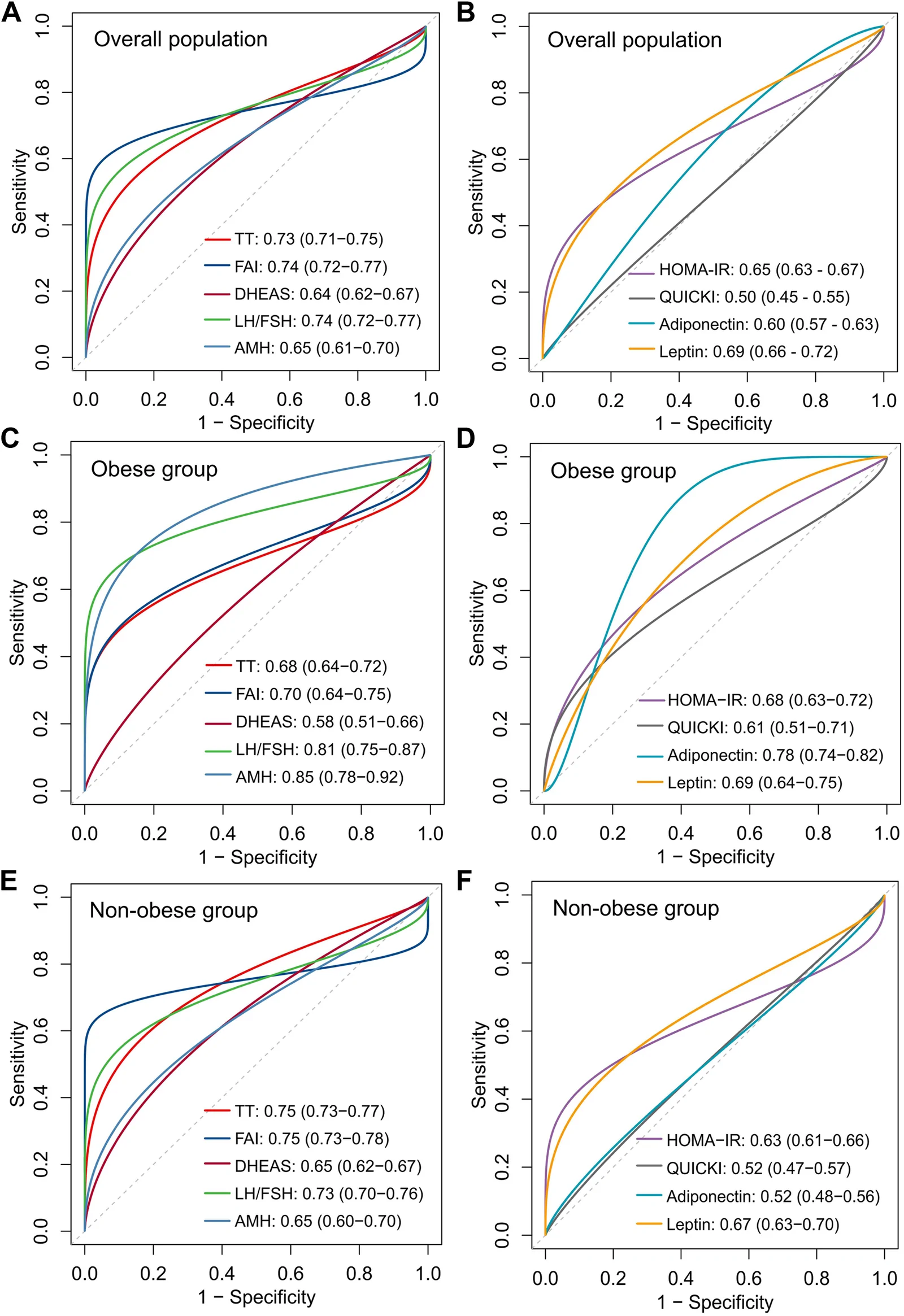
**Diagnostic performance of the biomarkers in overall adolescent PMOS overall and in subgroups stratified by BMI (A–F).** To illustrate the diagnostic performance of the biomarkers, receiver operating characteristic (ROC) curves of the biomarkers were generated in overall adolescents for (**A**) reproductive hormone parameters and (**B**) metabolic parameters, in the obese group for (**C**) reproductive hormone parameters and (**D**) metabolic parameters, and in the non-obese group for (**E**) reproductive hormone parameters and (**F**) metabolic parameters. Under a binormal assumption and moderate heterogeneity, the AUC of each biomarker was calculated, and ROC curves were generated from a series of cutoff values. Abbreviations: total testosterone (TT), free androgen index (FAI), ratio of luteinising hormone to follicle-stimulating hormone (LH/FSH), dehydroepiandrosterone sulphate (DHEAS), anti-Müllerian hormone (AMH), homoeostatic model assessment of insulin resistance (HOMA-IR), quantitative insulin sensitivity check index (QUICKI), area under the receiver operating characteristic curve (AUC).

**Table 2:** Predictive performance of the biomarkers.

Group	Biomarker	AUC	AUC (lower)	AUC (higher)	Cutoff	Sensitivity	Specificity
Overall population	TT (ng/dL)	0.73	0.71	0.75	55.96	0.54	0.86
	FAI	0.74	0.72	0.77	6.78	0.60	0.95
	DHEAS (μg/dL)	0.64	0.62	0.67	274.04	0.49	0.73
	LH/FSH	0.74	0.72	0.77	1.59	0.56	0.90
	AMH (ng/mL)	0.65	0.61	0.70	8.32	0.46	0.78
	HOMA-IR	0.65	0.63	0.67	4.35	0.43	0.86
	QUICKI	0.50	0.45	0.55	0.28	0.17	0.85
	Adiponectin (μg/mL)	0.60	0.57	0.63	9.77	0.71	0.44
	Leptin (ng/mL)	0.69	0.66	0.72	34.05	0.51	0.78
Obese group	TT (ng/dL)	0.68	0.64	0.72	61.53	0.48	0.90
	FAI	0.70	0.64	0.75	9.09	0.50	0.89
	DHEAS (μg/dL)	0.58	0.51	0.66	227.47	0.46	0.66
	LH/FSH	0.81	0.75	0.87	1.55	0.65	0.92
	AMH (ng/mL)	0.85	0.78	0.92	4.47	0.71	0.85
	HOMA-IR	0.68	0.63	0.72	6.18	0.52	0.75
	QUICKI	0.61	0.51	0.71	0.30	0.38	0.83
	Adiponectin (μg/mL)	0.78	0.74	0.82	10.92	0.88	0.60
	Leptin (ng/mL)	0.69	0.64	0.75	30.80	0.69	0.59
Non-obese group	TT (ng/dL)	0.75	0.73	0.77	53.79	0.58	0.83
	FAI	0.75	0.73	0.78	5.85	0.63	0.97
	DHEAS (μg/dL)	0.65	0.62	0.67	276.64	0.50	0.72
	LH/FSH	0.73	0.70	0.76	1.60	0.54	0.90
	AMH (ng/mL)	0.65	0.60	0.70	9.03	0.45	0.80
	HOMA-IR	0.63	0.61	0.66	3.49	0.42	0.90
	QUICKI	0.52	0.47	0.57	0.33	0.29	0.75
	Adiponectin (μg/mL)	0.52	0.48	0.56	3.88	0.24	0.82
	Leptin (ng/mL)	0.67	0.63	0.70	36.17	0.46	0.83
<17 years old group	TT (ng/dL)	0.76	0.73	0.78	53.66	0.59	0.81
	FAI	0.74	0.72	0.77	7.30	0.59	0.95
	DHEAS (μg/dL)	0.62	0.59	0.65	241.60	0.56	0.62
	LH/FSH	0.75	0.71	0.78	1.73	0.56	0.89
	AMH (ng/mL)	0.77	0.72	0.81	6.05	0.60	0.93
	HOMA-IR	0.63	0.61	0.66	4.76	0.43	0.81
	QUICKI	0.52	0.48	0.57	0.40	0.52	0.52
	Adiponectin (μg/mL)	0.57	0.54	0.61	8.10	0.55	0.56
	Leptin (ng/mL)	0.71	0.68	0.74	33.59	0.53	0.81
≥17 years old group	TT (ng/dL)	0.67	0.62	0.71	59.24	0.48	0.92
	FAI	0.76	0.72	0.81	5.54	0.62	0.95
	DHEAS (μg/dL)	0.70	0.66	0.74	308.64	0.50	0.84
	LH/FSH	0.74	0.70	0.79	1.36	0.56	0.92
	AMH (ng/mL)	0.56	0.47	0.64	11.74	0.32	0.79
	HOMA-IR	0.63	0.59	0.66	3.76	0.43	0.92
	QUICKI	0.63	0.47	0.80	0.34	0.41	0.85
	Adiponectin (μg/mL)	0.65	0.61	0.70	9.66	0.76	0.48
	Leptin (ng/mL)	0.61	0.54	0.68	33.32	0.45	0.72
NIH criteria group	TT (ng/dL)	0.78	0.75	0.80	48.51	0.61	0.83
	FAI	0.74	0.72	0.77	7.23	0.59	0.95
	DHEAS (μg/dL)	0.70	0.66	0.74	235.97	0.52	0.82
	LH/FSH	0.73	0.68	0.78	1.59	0.54	0.87
	AMH (ng/mL)	0.68	0.62	0.74	5.70	0.61	0.65
	HOMA-IR	0.64	0.61	0.67	4.50	0.42	0.86
	QUICKI	0.50	0.43	0.57	0.00	0.00	1.00
	Adiponectin (μg/mL)	0.57	0.54	0.61	9.00	0.80	0.34
	Leptin (ng/mL)	0.72	0.69	0.75	27.49	0.65	0.66
2 in 3, Rotterdam criteria group	TT (ng/dL)	0.69	0.66	0.72	65.68	0.49	0.86
	FAI	0.73	0.67	0.78	5.50	0.57	0.95
	DHEAS (μg/dL)	0.61	0.58	0.64	252.82	0.53	0.62
	LH/FSH	0.76	0.73	0.80	1.57	0.60	0.93
	AMH (ng/mL)	0.75	0.68	0.82	6.53	0.60	0.95
	HOMA-IR	0.66	0.62	0.70	4.65	0.44	0.84
	QUICKI	0.66	0.60	0.71	0.33	0.47	0.78
	Adiponectin (μg/mL)	0.72	0.67	0.77	12.43	0.75	0.58
	Leptin (ng/mL)	0.64	0.59	0.69	40.54	0.43	0.87
3 in 3, Rotterdam criteria group	TT (ng/dL)	0.96	0.93	1.00	57.34	0.88	0.95
	FAI	0.89	0.81	0.97	5.28	0.83	0.99
	DHEAS (μg/dL)	0.68	0.61	0.76	415.26	0.49	0.82
	LH/FSH	0.72	0.62	0.82	1.32	0.54	0.92
	AMH (ng/mL)	0.53	0.41	0.65	14.50	0.26	0.82
	HOMA-IR	0.67	0.60	0.74	2.55	0.48	0.93

Total testosterone (TT), free androgen index (FAI), ratio of luteinising hormone to follicle-stimulating hormone (LH/FSH), dehydroepiandrosterone sulphate (DHEA-S), antimullerian hormone (AMH), homoeostatic model assessment of insulin resistance (HOMA-IR), quantitative insulin sensitivity check index (QUICKI), area under the receiver operating characteristic curve (AUC).

We then assessed whether biomarkers showed subgroup-specific differences. TT and FAI showed increasing AUC underlying 2-of-3 Rotterdam criteria (TT = 0.69, FAI = 0.73), NIH criteria (TT = 0.78, FAI = 0.74), and 3-in-3 Rotterdam criteria (TT = 0.96, FAI = 0.89). Moreover, AMH outperformed other biomarkers in discriminating PMOS in adolescents with obesity (AUC = 0.85, threshold: 4.47 ng/mL), which was retained in the <17-year-old group (AUC = 0.77, threshold = 6.05 ng/mL). Similarly, adiponectin effectively characterised adolescent PMOS in the obese group (AUC = 0.78, threshold = 10.92 μg/mL). By contrast, leptin outperformed other biomarkers to reflect PMOS in the non-obese group (AUC = 0.67, threshold = 36.17 ng/mL), especially in early-to-mid puberty (AUC = 0.71, threshold = 33.32 ng/mL). In addition, DHEAS, LH/FSH, HOMA-IR, and QUICKI exhibited relatively stable performance to capture adolescent PMOS across subgroups (Fig. 4, Supplementary Fig. S6, and Table 2). Taken together, our findings support a comprehensive, stratified, biomarker-informed approach to adolescent PMOS diagnosis and metabolic assessment (Fig. 5).

**Fig. 5:**
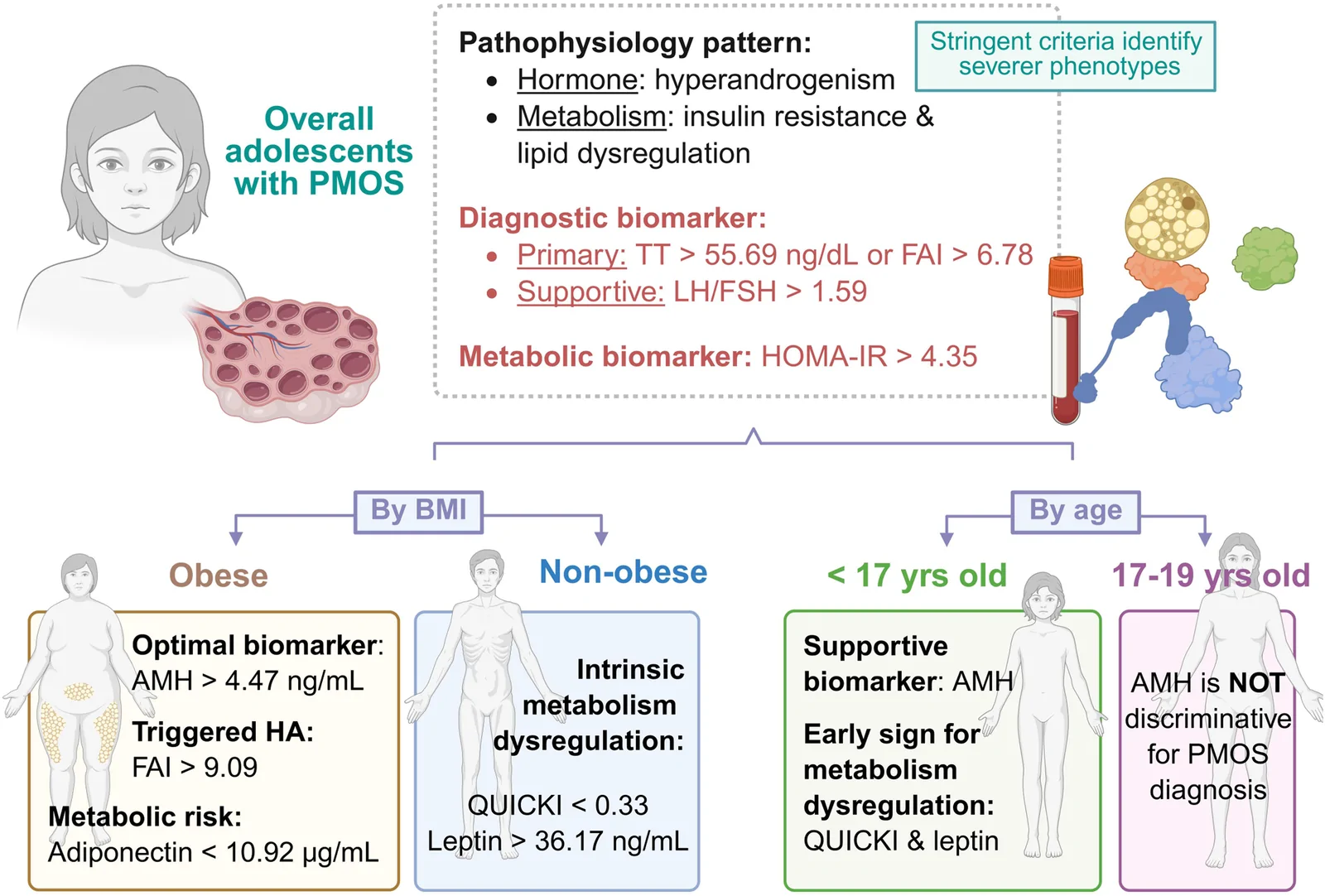
**Stratified biomarker-guided framework for the diagnosis and metabolic monitoring of adolescent PMOS.** The overall PMOS pathophysiology is characterised by hyperandrogenism, insulin resistance, and lipid dysregulation, in which stringent diagnostic criteria identify severe phenotypes. In the general population, TT > 55.69 ng/dL or FAI > 6.78 can serve as primary biomarkers to differentiate PMOS, while LH/FSH > 1.59 can support PMOS diagnosis. HOMA-IR > 4.35 can be used as a basic index for metabolic risk stratification. In particular, in adolescents with obesity, AMH > 4.47 ng/mL may be an optimal biomarker for PMOS diagnosis, while a higher threshold of FAI (>9.09) may be applied to define biochemical hyperandrogenism. Adiponectin < 10.92 μg/mL is an obesity-specific indicator for metabolic monitoring. In non-obese group, intrinsic metabolic dysregulation can be monitored through QUICKI < 0.33 and leptin > 36.17 ng/mL. Age-stratified analysis further reveals that in adolescents younger than 17 years old, AMH may be a supportive diagnostic biomarker, while QUICKI and leptin may be early indicators of metabolic dysregulation. Importantly, AMH is not discriminative for PMOS diagnosis in late puberty. Abbreviations: polyendocrine metabolic ovarian syndrome (PMOS), total testosterone (TT), free androgen index (FAI), ratio of luteinising hormone to follicle-stimulating hormone (LH/FSH), dehydroepiandrosterone sulphate (DHEAS), anti-Müllerian hormone (AMH), homoeostatic model assessment of insulin resistance (HOMA-IR), quantitative insulin sensitivity check index (QUICKI), hyperandrogenism (HA).

## Discussion

This systematic review and meta-analysis provides a comprehensive quantitative evaluation of endocrine and metabolic biomarkers for adolescent PMOS, integrating effect-size estimation, diagnostic-performance analysis, and cutoff-threshold evaluation. The pooled evidence suggests that TT and FAI are robust hormonal markers for adolescent PMOS diagnosis in general populations, whereas HOMA-IR remains the most consistent biomarker for metabolic assessment. Further stratified subgroup analysis revealed that AMH emerged as a discriminative biomarker in individuals with obesity during early-to-mid puberty, while adiponectin and leptin showed distinct metabolic relevance in the obese and non-obese groups, respectively. Moreover, consistent with the new nomenclature in the recent global consensus,^2^^,^^97^ our findings further provide biomarker-level evidence underscoring the endocrine and metabolic heterogeneity of adolescent PCOS.^98^ Collectively, these findings provide quantitative evidence for a phenotype-specific biomarker framework and support the implementation of age- and BMI-stratified approaches for the diagnosis and metabolic assessment of adolescent PMOS.

The transition from puberty to adulthood is accompanied by profound reproductive endocrine remodelling. For instance, LH/FSH ratio with a classic pattern of 2–3 reflects neuroendocrine dysregulation in adult PMOS.^99^ However, it showed a lower threshold of 1.59 for distinguishing PMOS in overall adolescents. In contrast, compared with a range of 2.50–4.97 for FAI in adults with PMOS,^100^^,^^101^ it was higher (at 6.78) in overall adolescents with PMOS and further increased in the obese group (at 9.09). Therefore, a higher FAI threshold may be considered in adolescents, especially for subjects with obesity. AMH is suggested as an alternative index for adult PMOS diagnosis.^1^ In our study, AMH exhibited a strong effect size in adolescents with obesity, with an AUC of 0.85 at a threshold of 4.47 ng/mL. However, its discriminative value was largely restricted to early-to-mid puberty (AUC = 0.77) but not in late adolescence (AUC = 0.56), possibly attributed to the physiological increases in antral follicle count and circulating AMH during pubertal maturation.^102^ Collectively, these findings are in agreement with the notion that PMOS exhibits distinct clinical and biochemical profiles between puberty and adulthood.^103^

PMOS is increasingly recognised as a heterogenous metabolic disorder.^2^ Obesity presents a major factor implicated in the development of PMOS.^2^ Consistent with this concept, our findings support the need for obesity-stratified strategies in metabolic assessment in adolescents with PMOS. Adiponectin, a metabolically beneficial adipokine with insulin-sensitising and anti-inflammatory properties,^104^ was markedly decreased in the obese group. In parallel, distinct decreased QUICKI and increased leptin were observed in non-obese group, further suggesting that metabolic dysfunction presents an intrinsic feature of adolescent PMOS.^2^ Similarly, pubertal development trajectory also modulates metabolic biomarkers. QUICKI and leptin were predominantly altered in patients under 17 years, aligning with the transient insulin resistance that is pronounced during early-to-mid adolescence.^105^ Therefore, BMI-age-specific strategies are warranted to enable timely metabolic risk identification and intervention for adolescent PMOS.

In addition, our study further evaluated the impact of diverse diagnostic criteria on biomarker performance. Previously, adolescent PMOS was commonly diagnosed using adult-derived criteria, including the 1990 NIH criteria (oligo-anovulation and hyperandrogenism) or the 2003 Rotterdam criteria (2 of 3 items among oligo-anovulation, hyperandrogenism, and PCOM).^26^^,^^27^ Moreover, some regions employ more conservative standards requiring fulfilment of all three items in the Rotterdam criteria.^106^ In our data, biomarkers showed broadly comparable effect sizes and diagnostic performance between NIH criteria and the 2-of-3 Rotterdam criteria. By contrast, one study employing 3-of-3 Rotterdam criteria demonstrated larger effect size for TT and FAI, likely reflecting a more pronounced hyperandrogenic phenotype. Therefore, these findings indicate that biomarker performance is generally robust across commonly used diagnostic criteria and the NIH-aligned approach may be sufficient for biomarker-based adolescent PMOS assessment. Nevertheless, prospective validation studies using the 2025 International adolescent-specific recommendations are needed to further calibrate biomarker thresholds and confirm their clinical utility.

Although our study comprehensively assessed reproductive and metabolic biomarkers in adolescent PMOS and provided the pooled estimates of diagnostic thresholds and effect sizes across subgroups, several limitations should be considered. First, diagnostic criteria varied across the included studies, and thresholds for biochemical hyperandrogenism were often predefined rather than derived from adolescent-specific reference populations. These inconsistencies may have introduced classification bias and influenced the pooled estimates of diagnostic cutoffs. Second, although controls were generally described as asymptomatic, subclinical PMOS features may not have been systematically excluded. This inclusion may reduce case–control differences and lead to underestimation of the biomarker effect sizes. Third, biomarker cutoffs were derived from pooled data across diverse populations. Given that hormonal profiles may substantially differ across ethnic groups, further validation is required to establish population-specific thresholds before clinical implementation. Fourth, we found publication bias existed in LH/FSH and DHEAS results, while adiponectin exhibited a limited effect size in high-quality studies. Therefore, their pooled effect sizes may be overestimated, and these results should be interpreted with caution. Moreover, methodological heterogeneity in biomarker measurement across studies may have affected the estimates. For instance, androgen assays using liquid chromatography-tandem mass spectrometry or immunoassays may have introduced variability in analytical sensitivity, specificity and cross-study comparability. Finally, our analysis was limited to the diagnostic value of individual biomarkers. Future research should develop and validate multi-biomarker panels integrating endocrine and metabolic profiles to determine whether combined models improve diagnostic accuracy, risk stratification, and clinical management in adolescent PMOS.

In conclusion, our study provides quantitative evidence supporting a stratified biomarker framework for the diagnosis and metabolic assessment in adolescent PMOS. In the overall adolescent population, TT and FAI emerged as the primary diagnostic biomarkers, supported by FSH/LH, while HOMA-IR served as the primary biomarker for metabolic assessment. Moreover, AMH, adiponectin, and leptin demonstrated phenotype-specific utility according to obesity status and pubertal stage. The biomarker-level evidence suggests that adolescent PMOS is a heterogeneous polyendocrine metabolic disorder, highlighting the need for age- and BMI-specific biomarker thresholds for precision diagnosis and individualised metabolic monitoring. Future large-scale, multiethnic adolescent cohorts are warranted to validate diagnostic performance and establish generalisable and reliable cutoffs in clinical implementation.

## Contributors

Y.W. formulated the research question and conceived the study. C.C.W. and E.S.V. formulated the inclusion and exclusion criteria and designed the search terms. Y.S. and C.C.W. screened the records, extracted data, and assessed the risk of bias and certainty of evidence. Y.S. performed the statistical analysis, and drafted the initial version of the manuscript. Y.W. and P.W.C revised the initial version of the manuscript. Y.W. had final responsibility for the decision to submit the manuscript for publication. All the authors interpreted the data, revised the manuscript, approved the final version of the manuscript, and had full access to all the data in the study.

## Data sharing statement

This meta-analysis is an analysis of previously published data without producing new data. Details of meta-analysis process are available on request to the corresponding author.

## Declaration of interests

The authors declare that they have no competing interests.
