# Supplementary material for: Serum biomarkers for the diagnosis and management of adolescent polyendocrine metabolic ovarian syndrome: a systematic review and meta-analysis
Source: eBioMedicine. 2026 Sep 24;132:106471. doi: 10.1016/j.ebiom.2026.106471 (PMC13634440; doi:10.1016/j.ebiom.2026.106471)
Supplement: Supplementary file 1 [file mmc1.pdf]

Supplementary Figure 1

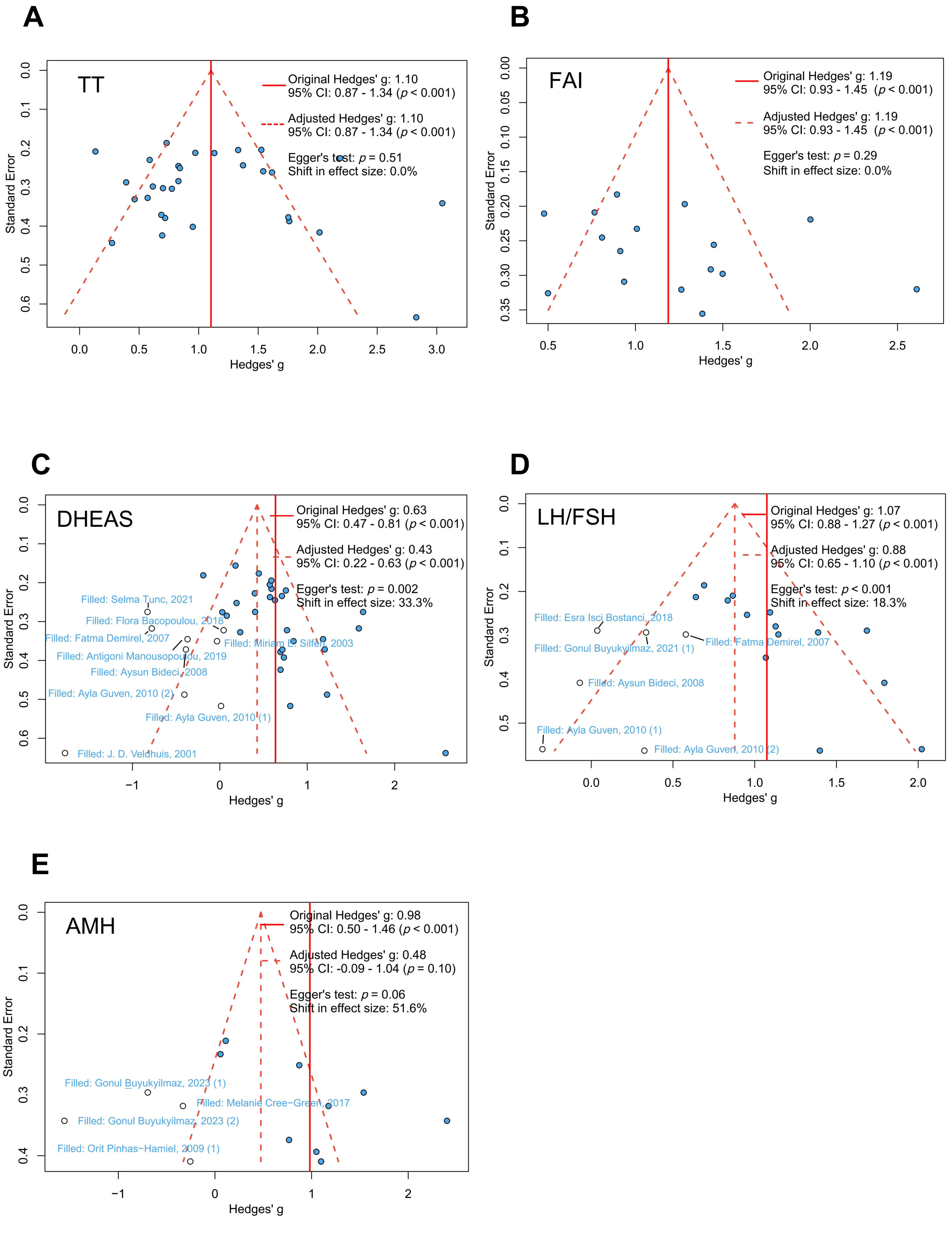

Supplementary Figure 1

(Continued)

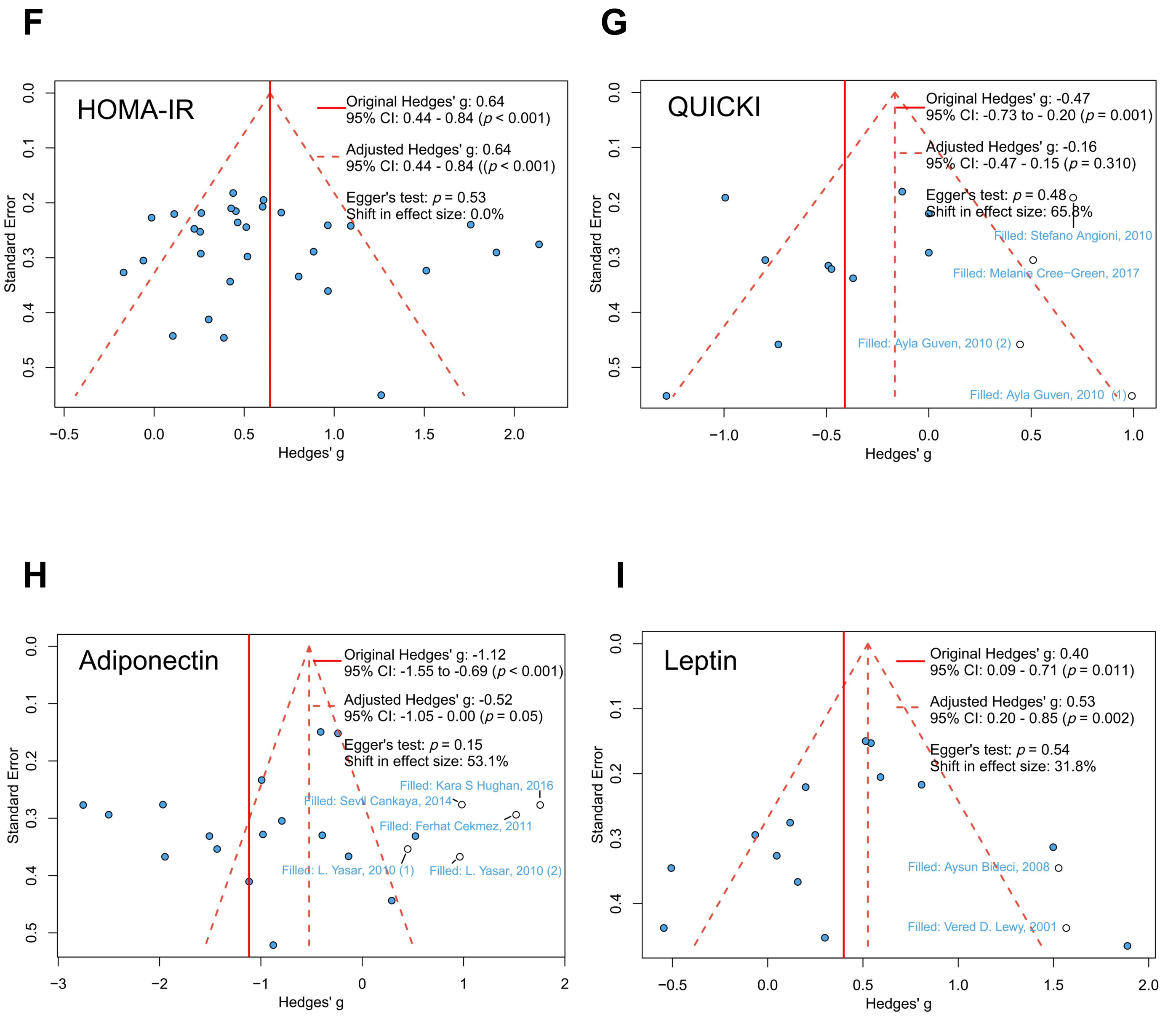

Supplementary figure 1. Publication bias assessment (A-I). Publication bias was assessed with Egger's regression test and funnel plot symmetry. Post-hoc trim-and-fill analyses were conducted to account for publication bias. Abbreviation: total testosterone (TT), free androgen index (FAI), ratio of luteinizing hormone to follicle-stimulating hormone (LH/FSH), dehydroepiandrosterone sulphate (DHEA-S), Anti-Müllerian hormone (AMH), homeostatic model assessment of insulin resistance (HOMA-IR), quantitative insulin sensitivity check index (QUICKI).

Supplementary Figure 2

A

TT

Study name	Hedges's g	SE	Lower	Upper	p-value
**Subgroup: NIH criteria**					
Vered D. Lewy, 2001	2.83	0.63	1.58	4.07	< 0.001
Miriam E. Silfen, 2003	2.02	0.42	1.20	2.83	< 0.001
Brooke Rossi, 2008	0.83	0.25	0.35	1.31	< 0.001
Franca Fruzzetti, 2009 (1)	0.97	0.21	0.56	1.39	< 0.001
Franca Fruzzetti, 2009 (2)	1.54	0.26	1.03	2.05	< 0.001
Gideon de Sousa, 2010	0.62	0.30	0.03	1.20	0.039
Paola Villa, 2012	2.19	0.23	1.75	2.63	< 0.001
Aviva B Sopher, 2012	0.69	0.42	−0.14	1.52	0.102
Aviva B Sopher, 2014	0.69	0.37	−0.04	1.41	0.064
Melanie Cree−Green, 2017	0.78	0.30	0.18	1.37	0.011
Sonali S Patel, 2017	0.70	0.30	0.11	1.29	0.021
Seda Ates, 2018	0.13	0.21	−0.28	0.54	0.523
Donna F Vine, 2020	1.33	0.20	0.93	1.73	< 0.001
Agnieszka Zachurzok, 2021	0.83	0.28	0.27	1.39	0.004
Gonul Buyukyilmaz, 2023	1.53	0.20	1.13	1.93	< 0.001
Karolina Skrzynska, 2024	1.62	0.26	1.10	2.13	< 0.001
Random effects model	1.16	0.16	0.85	1.47	< 0.001
Heterogeneity: $I^2 = 81.1\%$, $\tau^2 = 0.31$, $p < 0.001$					
**Subgroup: 2 in 3, Rotterdam criteria**					
Orit Pinhas−Hamiel, 2009 (1)	0.95	0.40	0.16	1.74	0.018
Orit Pinhas−Hamiel, 2009 (2)	0.72	0.38	−0.02	1.46	0.058
Xiaoli Chen, 2010	0.57	0.33	−0.07	1.21	0.081
Ayla Guven, 2010	0.27	0.44	−0.60	1.14	0.539
Stefano Angioni, 2010	0.73	0.19	0.37	1.10	< 0.001
Ferhat Cekmez, 2011	1.37	0.24	0.89	1.85	< 0.001
Tugba Kale−Gurbuz, 2013	0.46	0.33	−0.19	1.11	0.162
Sevil Cankaya, 2014	0.59	0.23	0.14	1.04	0.011
Flora Bacopoulou, 2018	1.76	0.39	1.00	2.52	< 0.001
Antigoni Manousopoulou, 2019	1.75	0.38	1.01	2.49	< 0.001
Ilker Tolga Ozgen, 2019	0.84	0.25	0.35	1.33	< 0.001
Elena Khashchenko, 2020	1.13	0.21	0.72	1.55	< 0.001
Giannouli, A, 2023	0.39	0.29	−0.17	0.95	0.174
**Random effects model**	0.88	0.12	0.65	1.12	< 0.001
Heterogeneity: $I^2 = 53.4\%$, $\tau^2 = 0.10$, $p = 0.012$					
**Subgroup: 3 in 3, Rotterdam criteria**					
Elif Sagsak, 2021	3.05	0.34	2.38	3.72	< 0.001
**Random effects model**	**1.10**	**0.12**	**0.87**	**1.34**	**< 0.001**
Heterogeneity: $I^2 = 80.1\%$, $\tau^2 = 0.34$, $p < 0.001$					
Test for subgroup differences: $\chi^2_2 = 35.88$, df = 2 ($p < 0.001$)					

Hedges's g and 95% CI

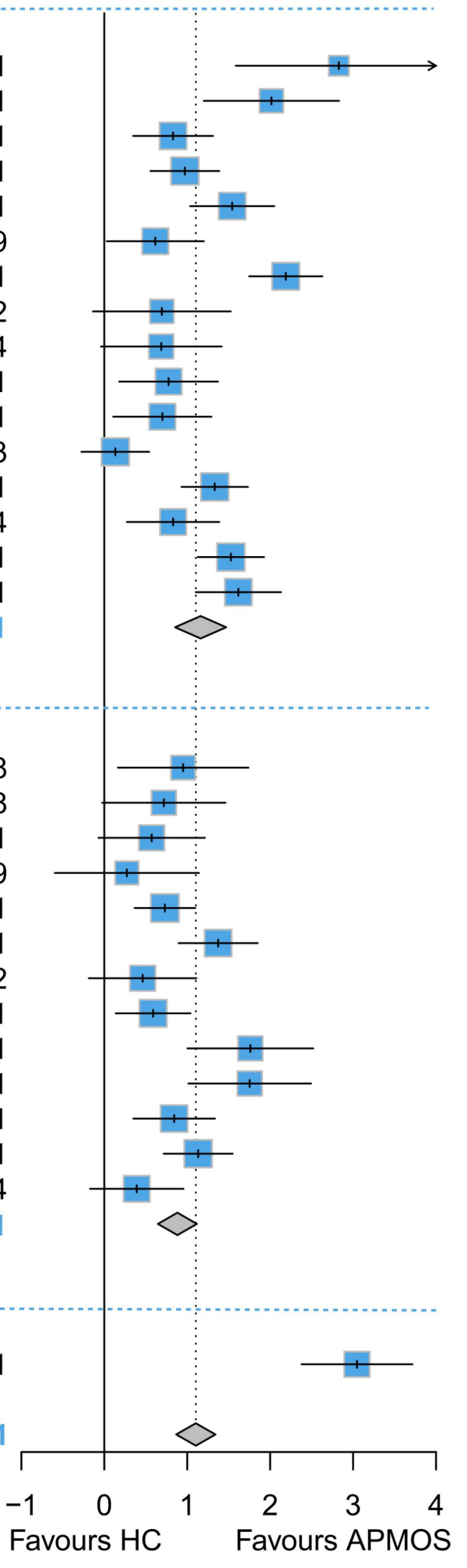

B

FAI

Study name	Hedges's g	SE	Lower	Upper	p-value
**Subgroup: NIH criteria**					
Brooke Rossi, 2008	0.81	0.25	0.33	1.29	< 0.001
Franca Fruzzetti, 2009 (1)	0.77	0.21	0.36	1.18	< 0.001
Franca Fruzzetti, 2009 (2)	1.45	0.26	0.95	1.95	< 0.001
Paola Villa, 2012	1.28	0.20	0.90	1.67	< 0.001
Melanie Cree Green, 2017	0.94	0.31	0.33	1.54	0.002
Sonali S Patel, 2017	1.26	0.32	0.63	1.89	< 0.001
Seda Ates, 2018	0.48	0.21	0.06	0.89	0.024
Donna F Vine, 2020	2.00	0.22	1.57	2.43	< 0.001
Merve Gungor, 2022	0.91	0.26	0.39	1.43	< 0.001
Gonul Buyukyilmaz, 2023	1.43	0.29	0.86	2.00	< 0.001
Gonul Buyukyilmaz, 2023	1.50	0.30	0.92	2.08	< 0.001
Random effects model	1.16	0.14	0.89	1.43	< 0.001
Heterogeneity: $I^2 = 72.3\%$, $\tau^2 = 0.15$, $p < 0.001$					
**Subgroup: 2 in 3, Rotterdam criteria**					
Xiaoli Chen, 2010	0.50	0.33	−0.14	1.14	0.125
Ferhat Cekmez, 2011	1.01	0.23	0.55	1.46	< 0.001
Antigoni Manousopoulou, 2019	1.38	0.36	0.69	2.08	< 0.001
Leyla Kara, 2025	0.89	0.18	0.54	1.25	< 0.001
Random effects model	0.93	0.12	0.69	1.17	< 0.001
Heterogeneity: $I^2 = 14.8\%$, $\tau^2 = < 0.001$, $p = 0.32$					
**Subgroup: 3 in 3, Rotterdam criteria**					
Elif Sagsak, 2021	2.61	0.32	1.98	3.24	< 0.001
**Random effects model**	**1.19**	**0.13**	**0.93**	**1.45**	**< 0.001**
Heterogeneity: $I^2 = 76.4\%$, $\tau^2 = 0.22$, $p < 0.001$					
Test for subgroup differences: $\chi^2_2 = 24.02$, df = 2 ($p < 0.001$)					

Hedges's g and 95% CI

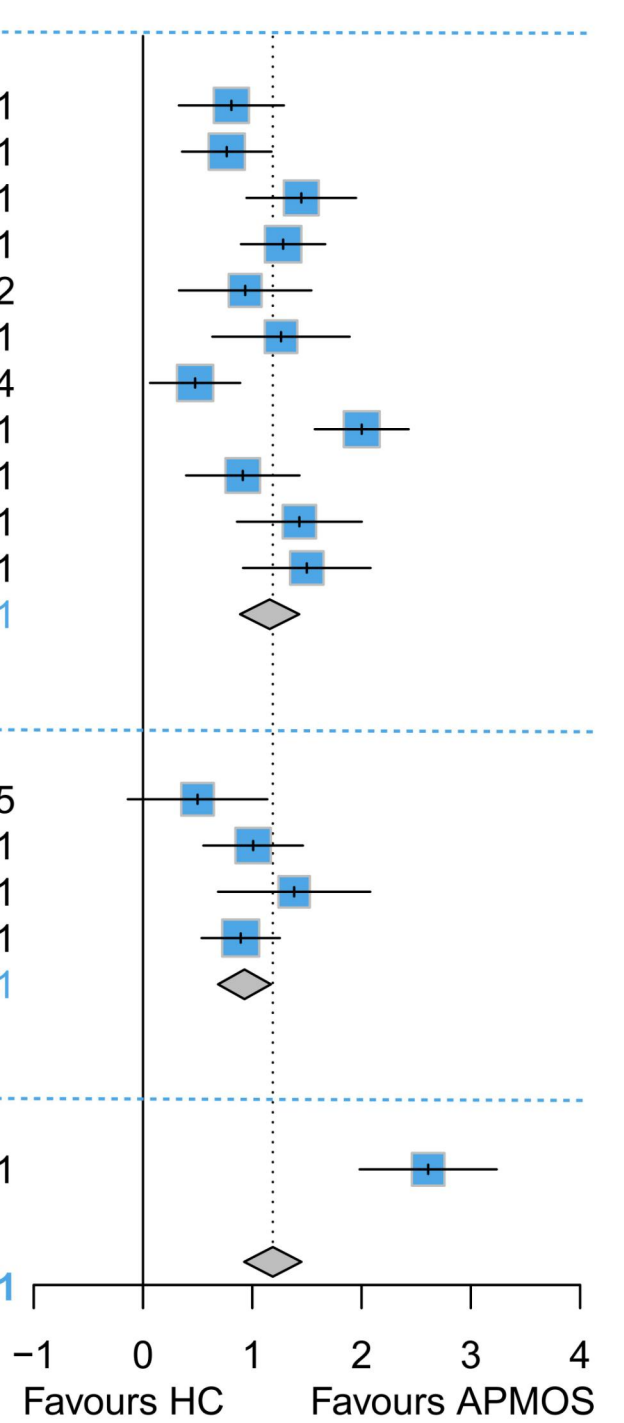

Supplementary Figure 2

(Continued)

C

DHEAS

Study name	Hedges's g	SE	Lower	Upper	*p*–value
**Subgroup: NIH criteria**					
J. D. Veldhuis, 2001	2.58	0.64	1.33	3.83	< 0.001
Miriam E. Silfen, 2003	0.84	0.35	0.16	1.53	0.016
Fatma Demirel, 2007	1.59	0.32	0.97	2.21	< 0.001
Aviva B Sopher, 2012	0.69	0.42	−0.14	1.53	0.102
Aviva B Sopher, 2014	0.72	0.37	−0.01	1.45	0.055
Claudio Villarroel, 2015	0.57	0.24	0.11	1.04	0.016
Donna F Vine, 2020	0.59	0.19	0.21	0.97	0.002
Agnieszka Zachurzok, 2021	0.40	0.28	−0.14	0.94	0.143
Merve Gungor, 2022	0.19	0.25	−0.30	0.69	0.450
Karolina Skrzynska, 2024	0.71	0.23	0.25	1.17	0.002
Random effects model	0.76	0.15	0.47	1.05	< 0.001
Heterogeneity: $I^2 = 60.1\%$, $\tau^2 = 0.12$, $p = 0.01$					
**Subgroup: 2 in 3, Rotterdam criteria**					
Aysun Bideci, 2008	1.20	0.37	0.47	1.93	0.001
Orit Pinhas−Hamiel, 2009 (1)	0.73	0.39	−0.04	1.50	0.062
Orit Pinhas−Hamiel, 2009 (2)	0.69	0.38	−0.05	1.43	0.067
Ayla Guven, 2010 (1)	0.81	0.52	−0.21	1.82	0.119
Ayla Guven, 2010 (2)	1.23	0.49	0.27	2.18	0.012
Stefano Angioni, 2010	−0.19	0.18	−0.55	0.16	0.294
Tugba Kale−Gurbuz, 2013	0.23	0.33	−0.41	0.87	0.477
Andrea Hsu Roe, 2013	0.18	0.16	−0.13	0.48	0.252
Sevil Cankaya, 2014	0.40	0.23	−0.05	0.85	0.078
Aylin Yetim, 2016	0.63	0.24	0.15	1.11	0.010
Flora Bacopoulou, 2018	0.77	0.32	0.14	1.40	0.017
Antigoni Manousopoulou, 2019	1.18	0.35	0.50	1.86	< 0.001
Elena Khashchenko, 2020	0.57	0.21	0.17	0.97	0.005
Selma Tunc, 2021	1.64	0.28	1.10	2.18	< 0.001
Giannouli. A, 2023 (2)	0.08	0.29	−0.48	0.64	0.782
Giannouli.A, 2023 (1)	0.03	0.28	−0.51	0.57	0.919
Leyla Kara, 2025	0.45	0.18	0.10	0.79	0.012
Random effects model	0.57	0.12	0.33	0.81	< 0.001
Heterogeneity: $I^2 = 69.8\%$, $\tau^2 = 0.17$ $p < 0.001$					
**Subgroup: 3 in 3, Rotterdam criteria**					
Aytekin Tokmak, 2015	0.59	0.22	0.17	1.01	0.006
Aytekin Tokmak, 2018	0.76	0.22	0.32	1.19	< 0.001
Random effects model	0.67	0.15	0.37	0.97	< 0.001
Heterogeneity: $I^2 = 0\%$, $\tau^2 = 0$, $p = 0.59$					
**Random effects model**	**0.64**	**0.09**	**0.47**	**0.81**	**< 0.001**
Heterogeneity: $I^2 = 65.5\%$, $\tau^2 = 0.13$, $p < 0.001$					
Test for subgroup differences: $\chi^2_2 = 0.97$, df = 2 ($p = 0.62$)					

Hedges's g and 95% CI

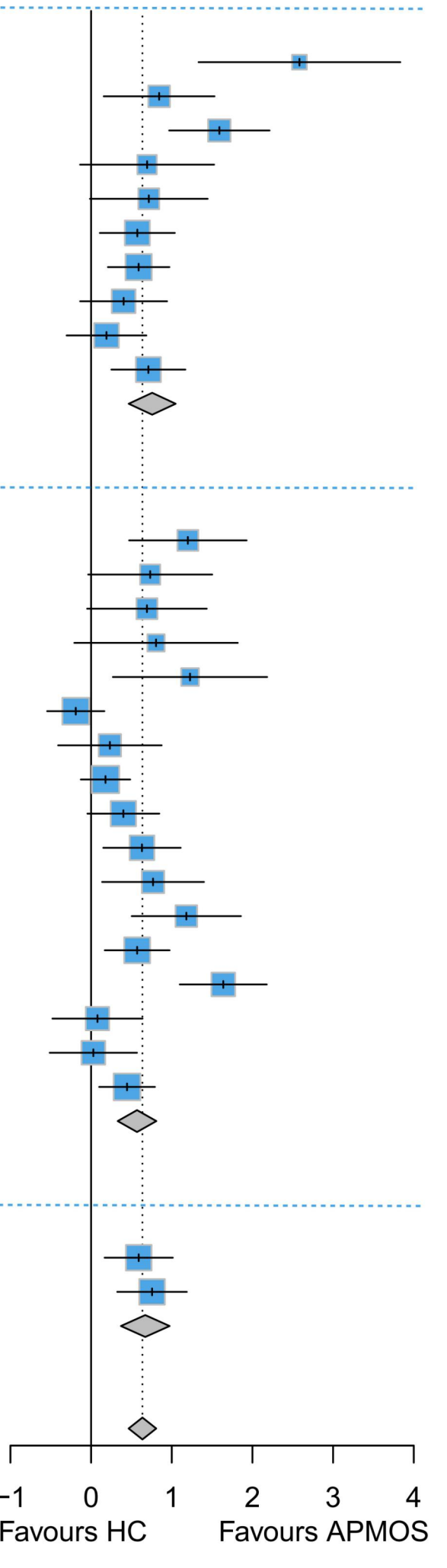

D

LH/FSH

Study name	Hedges's g	SE	Lower	Upper	*p*–value
**Subgroup: NIH criteria**					
Fatma Demirel, 2007	1.14	0.30	0.56	1.73	< 0.001
Claudio Villarroel, 2015	1.09	0.25	0.61	1.58	< 0.001
Seda Ates, 2018	0.64	0.21	0.22	1.06	0.003
Gonul Buyukyilmaz, 2023 (2)	1.13	0.28	0.58	1.68	< 0.001
Gonul Buyukyilmaz, 2023 (1)	1.39	0.29	0.81	1.96	< 0.001
Random effects model	1.04	0.14	0.77	1.31	< 0.001
Heterogeneity: $I^2 = 22.7\%$, $\tau^2 = 0.03$, $p = 0.27$					
**Subgroup: 2 in 3, Rotterdam**					
Aysun Bideci, 2008	1.79	0.41	0.99	2.59	< 0.001
Ayla Guven, 2010 (2)	1.40	0.56	0.30	2.50	0.013
Ayla Guven, 2010 (1)	2.02	0.56	0.93	3.12	< 0.001
Stefano Angioni, 2010	0.69	0.19	0.33	1.05	< 0.001
Tugba Kale−Gurbuz, 2013	1.07	0.35	0.38	1.76	0.002
Esra Isci Bostanci, 2018	1.69	0.29	1.12	2.25	< 0.001
Selma Tunc, 2021	0.95	0.25	0.46	1.45	< 0.001
Shivi Jain, 2021	0.87	0.21	0.46	1.28	< 0.001
Random effects model	1.19	0.17	0.86	1.52	< 0.001
Heterogeneity: $I^2 = 56.8\%$, $\tau^2 = 0.12$, $p = 0.02$					
**Subgroup: 3 in 3, Rotterdam**					
Aytekin Tokmak, 2015	0.83	0.22	0.40	1.27	< 0.001
**Random effects model**	**1.07**	**0.10**	**0.88**	**1.27**	**< 0.001**
Heterogeneity: $I^2 = 41.4\%$, $\tau^2 = 0.05$, $p = 0.05$					
Test for subgroup differences: $\chi^2_2 = 1.63$, df = 2 ($p = 0.44$)					

Hedges's g and 95% CI

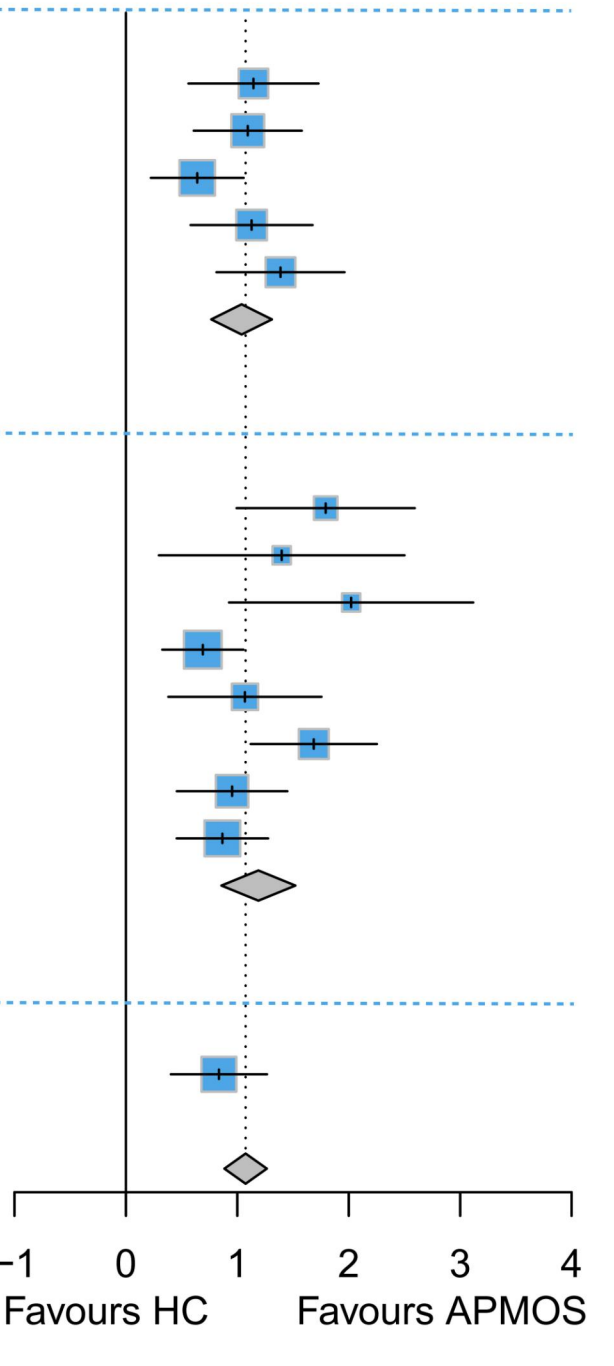

Supplementary Figure 2

(Continued)

E

AMH

Study name	Hedges's g	SE	Lower	Upper	p–value
**Subgroup: NIH criteria**					
Aviva B. Sopher, 2014	0.77	0.37	0.03	1.50	0.040
Claudio Villarroel, 2015	0.06	0.23	−0.40	0.51	0.806
Melanie Cree−Green, 2017	1.17	0.32	0.55	1.80	< 0.001
Gonul Buyukyilmaz, 2023 (1)	1.54	0.30	0.96	2.12	< 0.001
Gonul Buyukyilmaz, 2023 (2)	2.40	0.34	1.73	3.07	< 0.001
Random effects model	1.17	0.40	0.40	1.95	0.003
Heterogeneity: I^2 = 89.2%, τ^2 = 0.68, p < 0.001					
**Subgroup: 2 in 3, Rotterdam criteria**					
Orit Pinhas−Hamiel, 2009 (1)	1.10	0.41	0.30	1.90	0.007
Orit Pinhas−Hamiel, 2009 (2)	1.05	0.39	0.28	1.82	0.008
Selma Tunc, 2021	0.87	0.25	0.38	1.37	< 0.001
Random effects model	0.96	0.19	0.59	1.33	< 0.001
Heterogeneity: I^2 = 0%, τ^2 = 0, p = 0.87					
**Subgroup: 3 in 3, Rotterdam criteria**					
Aytekin Tokmak, 2015	0.11	0.21	−0.30	0.53	0.597
**Random effects model**	**0.98**	**0.24**	**0.50**	**1.46**	**< 0.001**

Heterogeneity: I^2 = 84.4%, τ^2 = 0.44, p < 0.001

Test for subgroup differences: χ^2_2 = 10.97, df = 2 (p = 0.004)

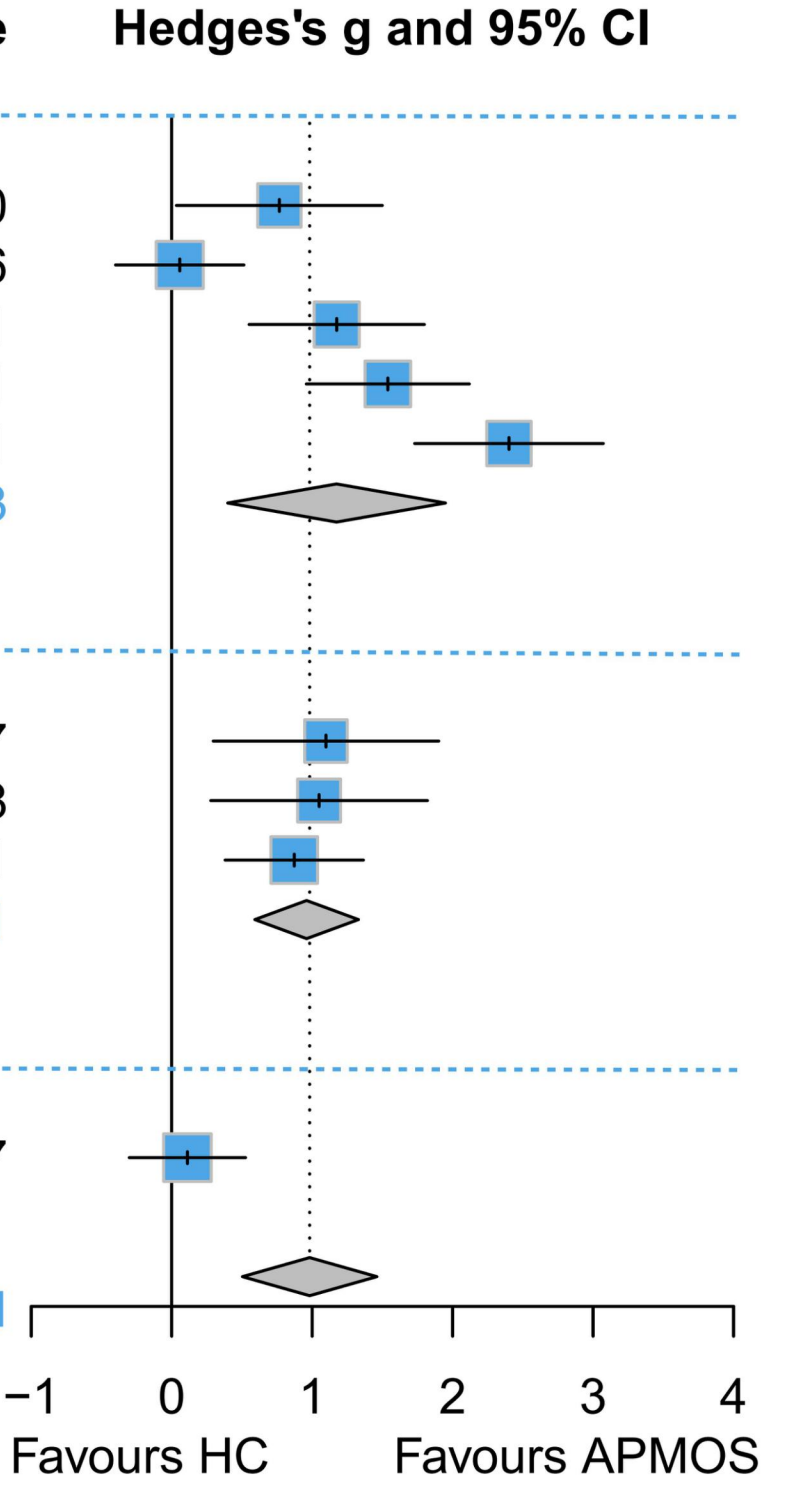

Supplementary figure 2. Effect sizes of the reproductive hormone levels in overall adolescent PMOS and subgroups stratified by criteria (A-E). Effect size was calculated using Hedges' *g*. Abbreviation: total testosterone (TT), free androgen index (FAI), ratio of luteinizing hormone to follicle-stimulating hormone (LH/FSH), dehydroepiandrosterone sulphate (DHEAS), Anti-Müllerian hormone (AMH), polyendocrine metabolic ovarian syndrome (PMOS), healthy control (HC), confidence interval (CI).

Supplementary Figure 3

A

TT

Study name	Hedges's g	SE	Lower	Upper	*p*−value
**Subgroup: < 17 years old**					
Vered D. Lewy, 2001	2.83	0.63	1.58	4.07	< 0.001
Miriam E. Silfen, 2003	2.02	0.42	1.20	2.83	< 0.001
Brooke Rossi, 2008	0.83	0.25	0.35	1.31	< 0.001
Franca Fruzzetti, 2009 (1)	0.97	0.21	0.56	1.39	< 0.001
Orit Pinhas−Hamiel, 2009 (1)	0.95	0.40	0.16	1.74	0.018
Orit Pinhas−Hamiel, 2009 (2)	0.72	0.38	−0.02	1.46	0.058
Ayla Guven, 2010	0.27	0.44	−0.60	1.14	0.539
Stefano Angioni, 2010	0.73	0.19	0.37	1.10	< 0.001
Gideon de Sousa, 2010	0.62	0.30	0.03	1.20	0.039
Ferhat Cekmez, 2011	1.37	0.24	0.89	1.85	< 0.001
Paola Villa, 2012	2.19	0.23	1.75	2.63	< 0.001
Aviva B Sopher, 2012	0.69	0.42	−0.14	1.52	0.102
Aviva B Sopher, 2014	0.69	0.37	−0.04	1.41	0.064
Melanie Cree−Green, 2017	0.78	0.30	0.18	1.37	0.011
Sonali S Patel, 2017	0.70	0.30	0.11	1.29	0.021
Flora Bacopoulou, 2018	1.76	0.39	1.00	2.52	< 0.001
Ilker Tolga Ozgen, 2019	0.84	0.25	0.35	1.33	< 0.001
Elena Khashchenko, 2020	1.13	0.21	0.72	1.55	< 0.001
Agnieszka Zachurzok, 2021	0.83	0.28	0.27	1.39	0.004
Elif Sagsak, 2021	3.05	0.34	2.38	3.72	< 0.001
Gonul Buyukyilmaz, 2023	1.53	0.20	1.13	1.93	< 0.001
Giannouli, A, 2023	0.39	0.29	−0.17	0.95	0.174
Karolina Skrzynska, 2024	1.62	0.26	1.10	2.13	< 0.001
Random effects model	1.17	0.14	0.89	1.45	< 0.001
Heterogeneity: $I^2 = 79.2\%$, $\tau^2 = 0.36$, $p < 0.001$					
**Subgroup: ⩾17 years old**					
Franca Fruzzetti (2), 2009	1.54	0.26	1.03	2.05	< 0.001
Xiaoli Chen, 2010	0.57	0.33	−0.07	1.21	0.081
Tugba Kale−Gurbuz, 2013ÿ	0.46	0.33	−0.19	1.11	0.162
Sevil Cankaya, 2014	0.59	0.23	0.14	1.04	0.011
Seda Ates, 2018	0.13	0.21	−0.28	0.54	0.523
Antigoni Manousopoulou, 2019	1.75	0.38	1.01	2.49	< 0.001
Donna F Vine, 2020	1.33	0.20	0.93	1.73	< 0.001
Random effects model	0.90	0.23	0.44	1.35	< 0.001
Heterogeneity: $I^2 = 82.2\%$, $\tau^2 = 0.30$, $p < 0.001$					
**Random effects model**	**1.10**	**0.12**	**0.87**	**1.34**	**< 0.001**

Heterogeneity: $I^2 = 80.1\%$, $\tau^2 = 0.34$, $p < 0.001$
Test for subgroup differences: $\chi^2_1 = 1.00$, df = 1 ($p = 0.32$)

Hedges's g and 95% CI

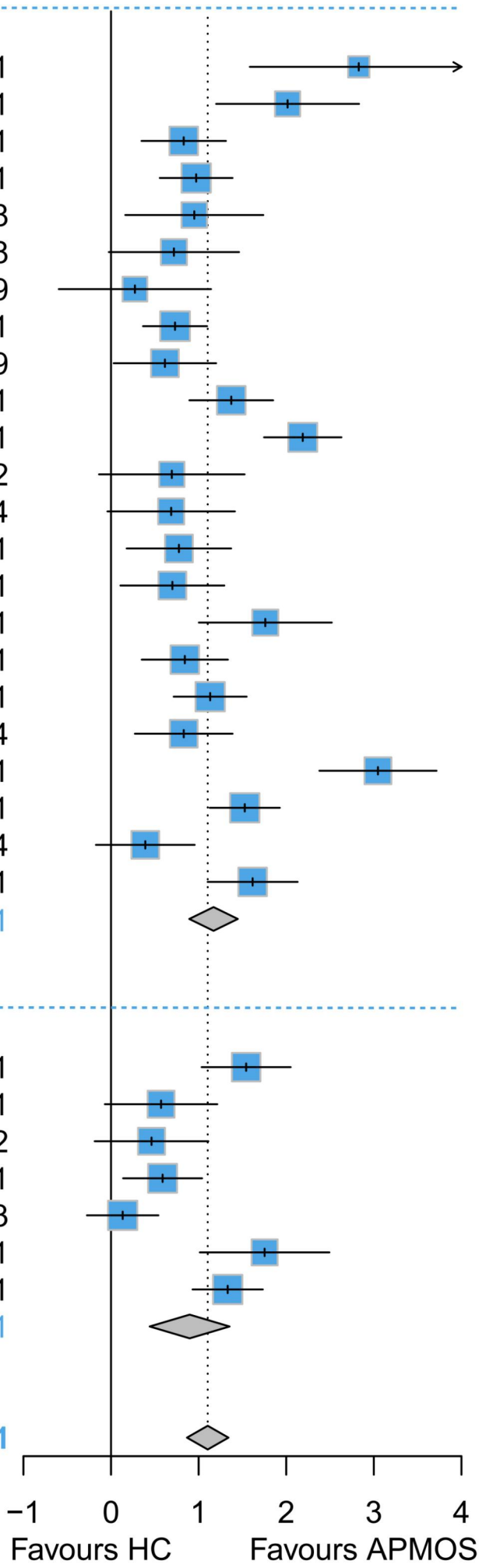

B

FAI

Study name	Hedges's g	SE	Lower	Upper	*p*−value
**Subgroup: < 17 years old**					
Brooke Rossi, 2008	0.81	0.25	0.33	1.29	< 0.001
Franca Fruzzetti, 2009 (1)	0.77	0.21	0.36	1.18	< 0.001
Ferhat Cekmez, 2011	1.01	0.23	0.55	1.46	< 0.001
Paola Villa, 2012	1.28	0.20	0.90	1.67	< 0.001
Melanie Cree Green, 2017	0.94	0.31	0.33	1.54	0.002
Sonali S Patel, 2017	1.26	0.32	0.63	1.89	< 0.001
Elif Sagsak, 2021	2.61	0.32	1.98	3.24	< 0.001
Merve Gungor, 2022	0.91	0.26	0.39	1.43	< 0.001
Gonul Buyukyilmaz, 2023	1.43	0.29	0.86	2.00	< 0.001
Gonul Buyukyilmaz, 2023	1.50	0.30	0.92	2.08	< 0.001
Leyla Kara, 2025	0.89	0.18	0.54	1.25	< 0.001
Random effects model	1.19	0.15	0.91	1.48	< 0.001
Heterogeneity: $I^2 = 69.2\%$, $\tau^2 = 0.17$, $p < 0.001$					
**Subgroup: ⩾17 years old**					
Franca Fruzzetti, 2009 (2)	1.45	0.26	0.95	1.95	< 0.001
Xiaoli Chen, 2010	0.50	0.33	−0.14	1.14	0.125
Seda Ates, 2018	0.48	0.21	0.06	0.89	0.024
Antigoni Manousopoulou, 2019	1.38	0.36	0.69	2.08	< 0.001
Donna F Vine, 2020	2.00	0.22	1.57	2.43	< 0.001
Random effects model	1.17	0.30	0.57	1.76	< 0.001
Heterogeneity: $I^2 = 87.1\%$, $\tau^2 = 0.39$, $p < 0.001$					
**Random effects model**	**1.19**	**0.13**	**0.93**	**1.45**	**< 0.001**

Heterogeneity: $I^2 = 76.4\%$, $\tau^2 = 0.22$, $p < 0.001$
Test for subgroup differences: $\chi^2_1 = 0.00$, df = 1 ($p = 0.95$)

Hedges's g and 95% CI

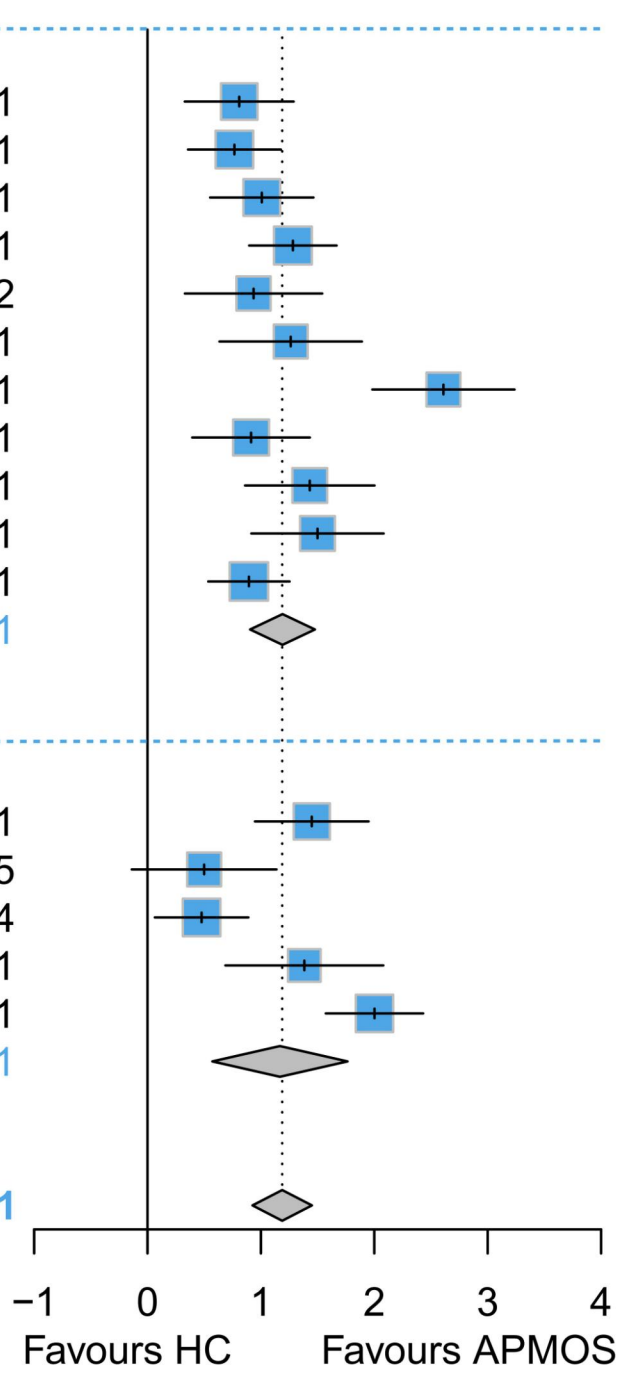

Supplementary Figure 3

(Continued)

C

DHEAS

Study name	Hedges's g	SE	Lower	Upper	*p*–value
**Subgroup: < 17 years old**					
J. D. Veldhuis, 2001	2.58	0.64	1.33	3.83	< 0.001
Miriam E. Silfen, 2003	0.84	0.35	0.16	1.53	0.016
Fatma Demirel, 2007	1.59	0.32	0.97	2.21	< 0.001
Aysun Bideci, 2008	1.20	0.37	0.47	1.93	0.001
Orit Pinhas−Hamiel, 2009 (1)	0.73	0.39	−0.04	1.50	0.062
Orit Pinhas−Hamiel, 2009 (2)	0.69	0.38	−0.05	1.43	0.067
Ayla Guven, 2010 (1)	0.81	0.52	−0.21	1.82	0.119
Ayla Guven, 2010 (2)	1.23	0.49	0.27	2.18	0.012
Stefano Angioni, 2010	−0.19	0.18	−0.55	0.16	0.294
Aviva B Sopher, 2012	0.69	0.42	−0.14	1.53	0.102
Andrea Hsu Roe, 2013	0.18	0.16	−0.13	0.48	0.252
Aviva B Sopher, 2014	0.72	0.37	−0.01	1.45	0.055
Aylin Yetim, 2016	0.63	0.24	0.15	1.11	0.010
Flora Bacopoulou, 2018	0.77	0.32	0.14	1.40	0.017
Elena Khashchenko, 2020	0.57	0.21	0.17	0.97	0.005
Selma Tunc, 2021	1.64	0.28	1.10	2.18	< 0.001
Agnieszka Zachurzok, 2021	0.40	0.28	−0.14	0.94	0.143
Merve Gungor, 2022	0.19	0.25	−0.30	0.69	0.450
Giannouli. A, 2023 (2)	0.08	0.29	−0.48	0.64	0.782
Giannouli. A, 2023 (1)	0.03	0.28	−0.51	0.57	0.919
Karolina Skrzynska, 2024	0.71	0.23	0.25	1.17	0.002
Leyla Kara, 2025	0.45	0.18	0.10	0.79	0.012
Random effects model	0.67	0.12	0.44	0.89	< 0.001
Heterogeneity: $I^2 = 72.1\%$, $\tau^2 = 0.20$, $p < 0.001$					
**Subgroup: ⩾ 17 years old**					
Tugba Kale−Gurbuz, 2013	0.23	0.33	−0.41	0.87	0.477
Sevil Cankaya, 2014	0.40	0.23	−0.05	0.85	0.078
Claudio Villarroel, 2015	0.57	0.24	0.11	1.04	0.016
Aytekin Tokmak, 2015	0.59	0.22	0.17	1.01	0.006
Aytekin Tokmak, 2018	0.76	0.22	0.32	1.19	< 0.001
Antigoni Manousopoulou, 2019	1.18	0.35	0.50	1.86	< 0.001
Donna F Vine, 2020	0.59	0.19	0.21	0.97	0.002
Random effects model	0.60	0.09	0.42	0.78	< 0.001
Heterogeneity: $I^2 = 0\%$, $\tau^2 = < 0.001$, $p = 0.50$					
**Random effects model**	**0.64**	**0.09**	**0.47**	**0.81**	**< 0.001**
Heterogeneity: $I^2 = 65.5\%$, $\tau^2 = 0.13$, $p < 0.001$					
Test for subgroup differences: $\chi^2_1 = 0.21$, df = 1 ($p = 0.65$)					

Hedges's g and 95% CI

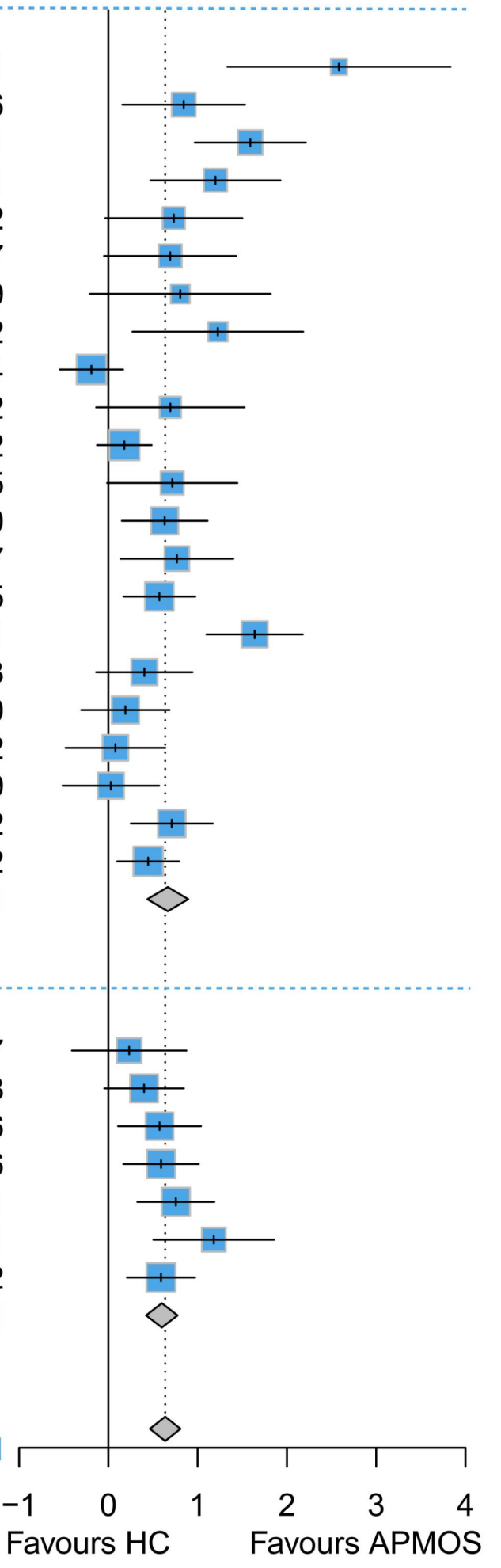

D

LH/FSH

Study name	Hedges's g	SE	Lower	Upper	*p*–value
**Subgroup: < 17 years old**					
Fatma Demirel, 2007	1.14	0.30	0.56	1.73	< 0.001
Aysun Bideci, 2008	1.79	0.41	0.99	2.59	< 0.001
Ayla Guven, 2010 (2)	1.40	0.56	0.30	2.50	0.013
Ayla Guven, 2010 (1)	2.02	0.56	0.93	3.12	< 0.001
Stefano Angioni, 2010	0.69	0.19	0.33	1.05	< 0.001
Selma Tunc, 2021	0.95	0.25	0.46	1.45	< 0.001
Shivi Jain, 2021	0.87	0.21	0.46	1.28	< 0.001
Gonul Buyukyilmaz, 2023 (2)	1.13	0.28	0.58	1.68	< 0.001
Gonul Buyukyilmaz, 2023 (1)	1.39	0.29	0.81	1.96	< 0.001
Random effects model	1.11	0.12	0.86	1.35	< 0.001
Heterogeneity: $I^2 = 37.8\%$, $\tau^2 = 0.05$, $p = 0.12$					
**Subgroup: ⩾ 17 years old**					
Tugba Kale−Gurbuz, 2013	1.07	0.35	0.38	1.76	0.002
Claudio Villarroel, 2015	1.09	0.25	0.61	1.58	< 0.001
Aytekin Tokmak, 2015	0.83	0.22	0.40	1.27	< 0.001
Esra Isci Bostanci, 2018	1.69	0.29	1.12	2.25	< 0.001
Seda Ates, 2018	0.64	0.21	0.22	1.06	0.003
Random effects model	1.03	0.18	0.69	1.38	< 0.001
Heterogeneity: $I^2 = 56.6\%$, $\tau^2 = 0.09$, $p = 0.06$					
**Random effects model**	**1.07**	**0.10**	**0.88**	**1.27**	**< 0.001**
Heterogeneity: $I^2 = 41.4\%$, $\tau^2 = 0.05$, $p = 0.05$					
Test for subgroup differences: $\chi^2_1 = 0.12$, df = 1 ($p = 0.73$)					

Hedges's g and 95% CI

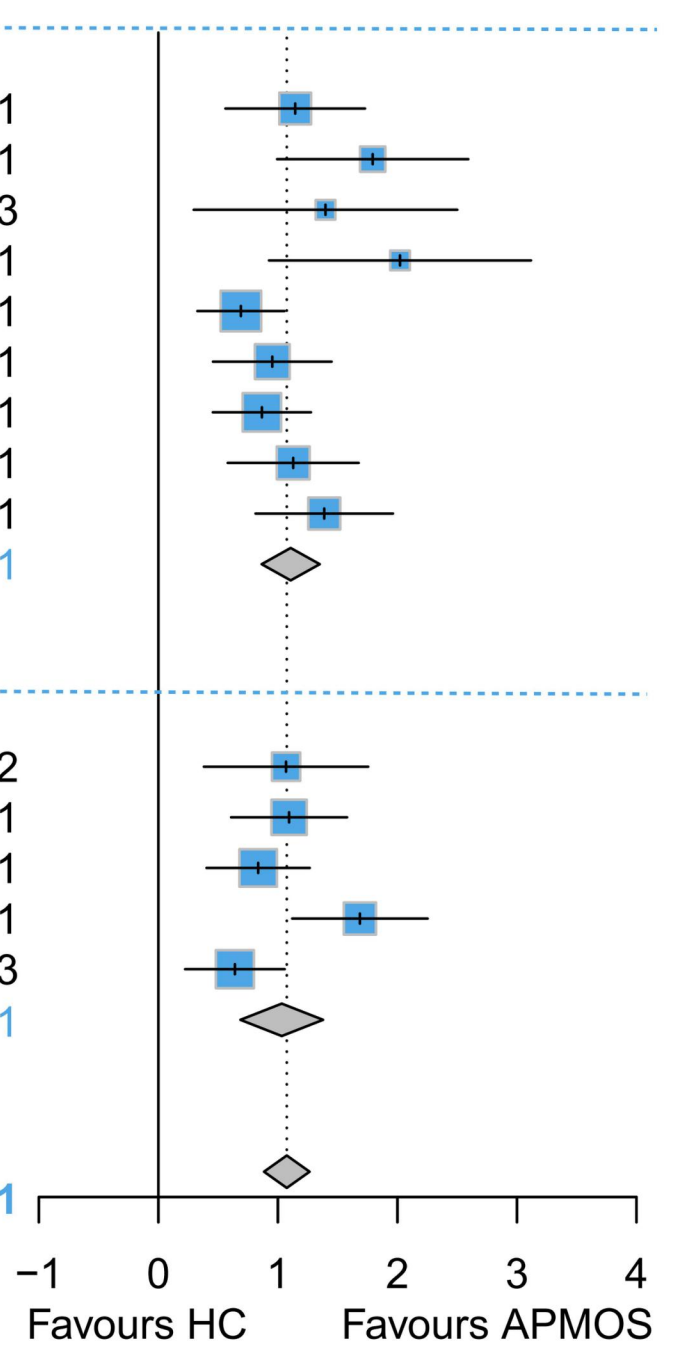

Supplementary Figure 3

(Continued)

E

AMH

Study name	Hedges's g	SE	Lower	Upper	*p*-value
**Subgroup: < 17 years old**					
Orit Pinhas−Hamiel, 2009 (1)	1.10	0.41	0.30	1.90	0.007
Orit Pinhas−Hamiel, 2009 (2)	1.05	0.39	0.28	1.82	0.008
Aviva B. Sopher, 2014	0.77	0.37	0.03	1.50	0.040
Melanie Cree−Green, 2017	1.17	0.32	0.55	1.80	< 0.001
Selma Tunc, 2021	0.87	0.25	0.38	1.37	< 0.001
Gonul Buyukyilmaz, 2023 (1)	1.54	0.30	0.96	2.12	< 0.001
Gonul Buyukyilmaz, 2023 (2)	2.40	0.34	1.73	3.07	< 0.001
Random effects model	1.28	0.21	0.86	1.69	< 0.001
Heterogeneity: $I^2 = 63.9\%$, $\tau^2 = 0.19$, $p = 0.01$					
**Subgroup: ⩾ 17 years old**					
Claudio Villarroel, 2015	0.06	0.23	−0.40	0.51	0.806
Aytekin Tokmak, 2015	0.11	0.21	−0.30	0.53	0.597
Random effects model	0.09	0.16	−0.22	0.39	0.578
Heterogeneity: $I^2 = 0\%$, $\tau^2 = 0$, $p = 0.86$					
**Random effects model**	**0.98**	**0.24**	**0.50**	**1.46**	**< 0.001**
Heterogeneity: $I^2 = 84.4\%$, $\tau^2 = 0.44$, $p < 0.001$					
Test for subgroup differences: $\chi^2_1 = 20.47$, df = 1 ($p < 0.001$)					

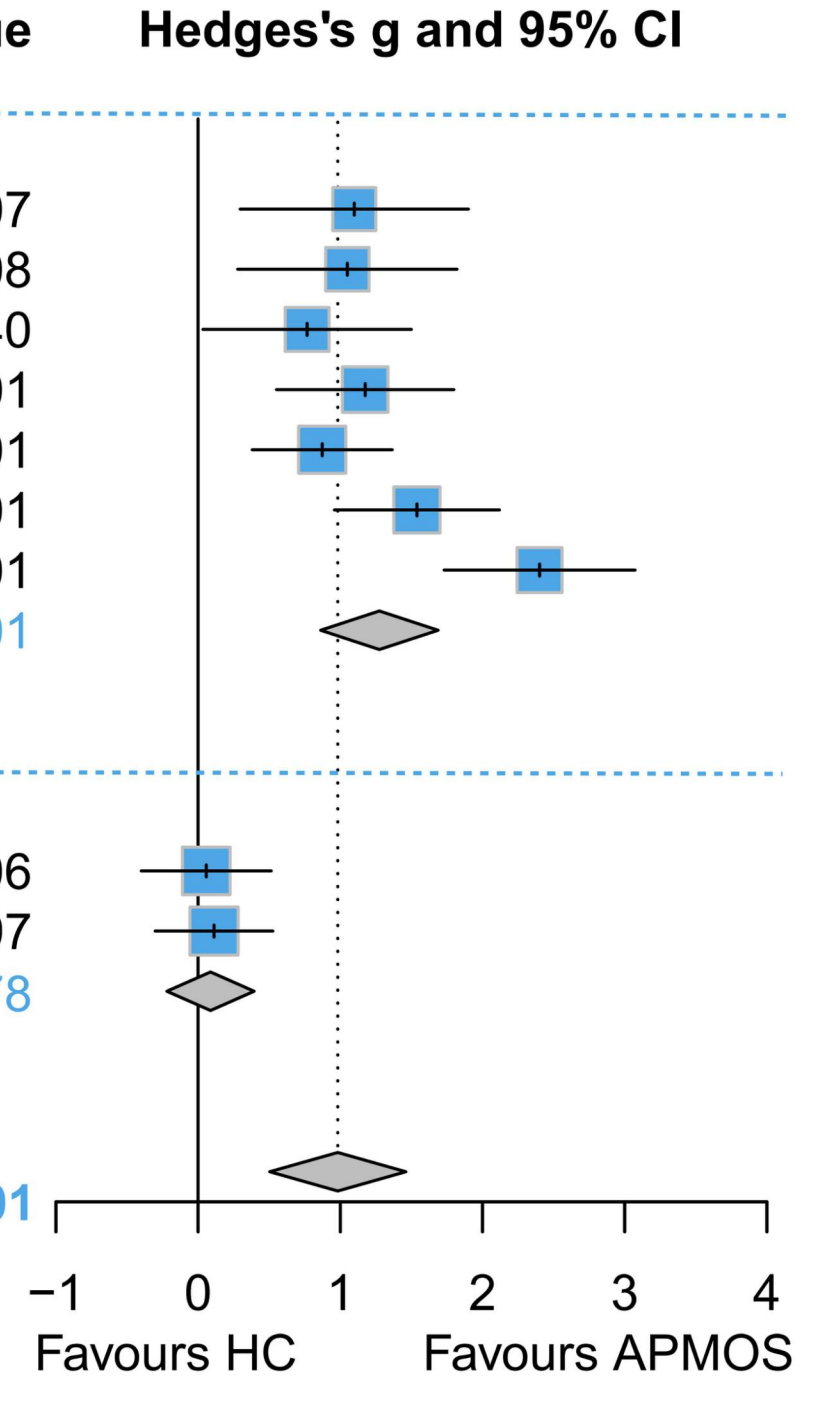

Supplementary figure 3. Effect sizes of the reproductive hormone levels in overall adolescent PMOS and subgroups stratified by age (A-E). Effect size was calculated using Hedges' *g*. Abbreviation: total testosterone (TT), free androgen index (FAI), ratio of luteinizing hormone to follicle-stimulating hormone (LH/FSH), dehydroepiandrosterone sulphate (DHEAS), Anti-Müllerian hormone (AMH), polyendocrine metabolic ovarian syndrome (PMOS), healthy control (HC), confidence interval (CI).

Supplementary Figure 4

A

HOMA-IR

Study name	Hedges's g	SE	Lower	Upper	p–value
**Subgroup: < 17 years old**					
Fatma Demirel, 2007	0.89	0.29	0.32	1.45	0.002
Aysun Bideci, 2008	0.42	0.34	−0.25	1.10	0.219
Franca Fruzzetti, 2009 (1)	0.60	0.21	0.20	1.01	0.004
Ayla Guven, 2010 (1)	1.26	0.55	0.18	2.34	0.022
Ayla Guven, 2010 (2)	0.39	0.45	−0.49	1.26	0.385
Gideon de Sousa, 2010	0.26	0.29	−0.31	0.83	0.376
Ferhat Cekmez, 2011	2.14	0.28	1.60	2.68	< 0.001
Aviva B Sopher, 2012	0.30	0.41	−0.51	1.11	0.463
Paola Villa, 2012	0.44	0.18	0.08	0.80	0.016
Melissa Pawelczak, 2014	0.10	0.44	−0.76	0.97	0.815
Kara S Hughan, 2016	1.76	0.24	1.29	2.23	< 0.001
Melanie Cree−Green, 2017	0.52	0.30	−0.07	1.10	0.082
Ilker Tolga Ozgen, 2019	0.51	0.24	0.03	0.99	0.036
Elif Sagsak, 2021	1.90	0.29	1.33	2.47	< 0.001
Elvan Bayramoglu, 2021 (1)	−0.06	0.31	−0.66	0.54	0.844
Elvan Bayramoglu, 2021 (2)	0.97	0.36	0.26	1.67	0.007
Merve Gungor, 2022	0.26	0.25	−0.24	0.75	0.312
Karolina Skrzynska, 2024	−0.01	0.23	−0.46	0.43	0.950
Elzbieta Forys, 2025	0.11	0.22	−0.32	0.54	0.613
Random effects model	0.67	0.15	0.37	0.97	< 0.001
Heterogeneity: $I^2 = 82.1\%$, $\tau^2 = 0.35$, $p < 0.001$					
**Subgroup: ⩾17 years old**					
Franca Fruzzetti, 2009 (2)	0.96	0.24	0.49	1.44	< 0.001
Xiaoli Chen, 2010	0.80	0.33	0.15	1.46	0.016
Haleh Rahmanpour, 2012	0.26	0.22	−0.17	0.69	0.230
Ruya Deveer, 2012	1.51	0.32	0.88	2.15	< 0.001
Tugba Kale−Gurbuz, 2013	−0.17	0.33	−0.81	0.47	0.604
Sevil Cankaya, 2014	1.09	0.24	0.62	1.57	< 0.001
Claudio Villarroel, 2015	0.46	0.24	0.00	0.93	0.049
Aytekin Tokmak, 2015	0.71	0.22	0.28	1.13	0.001
Esra Isci Bostanci, 2018	0.22	0.25	−0.26	0.71	0.368
Aytekin Tokmak, 2018	0.45	0.22	0.03	0.88	0.035
Seda Ates, 2018	0.43	0.21	0.02	0.84	0.042
Donna F Vine, 2020	0.61	0.19	0.23	0.99	0.002
Random effects model	0.60	0.11	0.38	0.82	< 0.001
Heterogeneity: $I^2 = 58.7\%$, $\tau^2 = 0.09$, $p = 0.005$					
**Random effects model**	**0.64**	**0.10**	**0.44**	**0.84**	**< 0.001**
Heterogeneity: $I^2 = 76.5\%$, $\tau^2 = 0.24$, $p < 0.001$					
Test for subgroup differences: $\chi^2_1 = 0.13$, df = 1 ($p = 0.72$)					

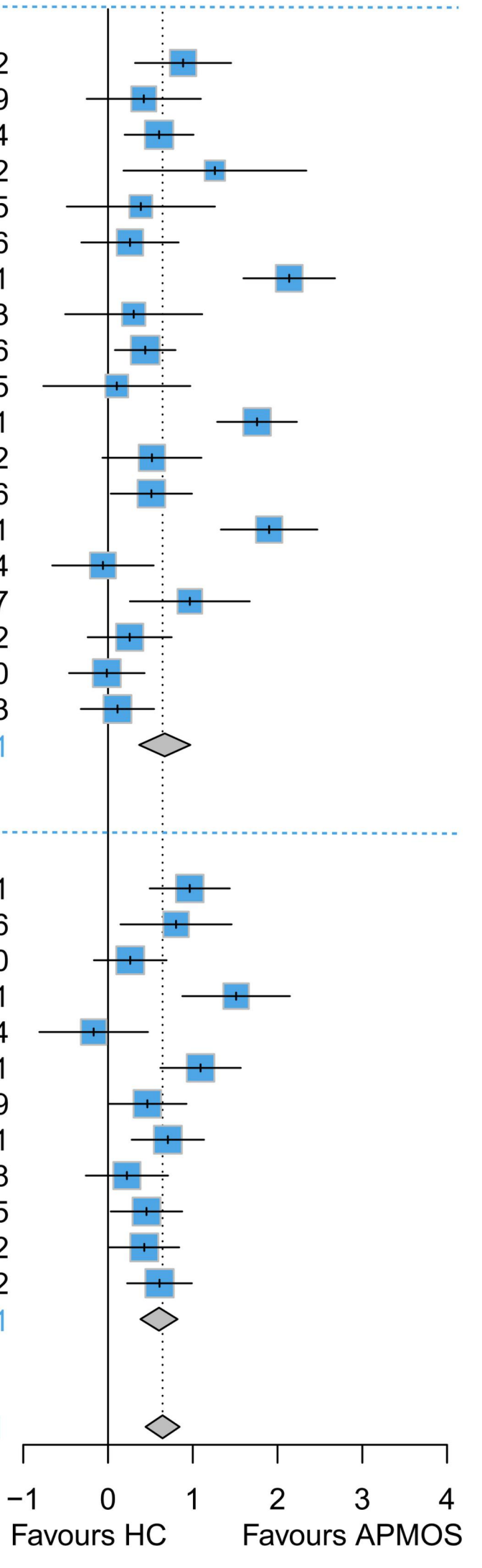

B

QUICKI

Study name	Hedges's g	SE	Lower	Upper	p–value
**Subgroup: < 17 years old**					
Miriam E. Silfen, 2003	−0.37	0.34	−1.03	0.29	0.274
Ayla Guven, 2010 (1)	−1.28	0.55	−2.36	−0.20	0.020
Ayla Guven, 2010 (2)	−0.73	0.46	−1.63	0.16	0.109
Gideon de Sousa, 2010	0.00	0.29	−0.57	0.57	1.000
Stefano Angioni, 2010	−0.99	0.19	−1.37	−0.62	< 0.001
Paola Villa, 2012	−0.13	0.18	−0.48	0.22	0.472
Melanie Cree−Green, 2017	−0.80	0.30	−1.40	−0.20	0.009
Flora Bacopoulou, 2018	−0.49	0.32	−1.11	0.13	0.119
Elzbieta Forys, 2025	0.00	0.22	−0.43	0.43	1.000
Random effects model	−0.47	0.15	−0.76	−0.17	0.002
Heterogeneity: $I^2 = 63.3\%$, $\tau^2 = 0.11$, $p = 0.005$					
**Subgroup: ⩾17 years old**					
Antigoni Manousopoulou, 2019	−0.48	0.32	−1.10	0.15	0.139
**Random effects model**	**−0.47**	**0.14**	**−0.73**	**−0.20**	**0.001**
Heterogeneity: $I^2 = 58.8\%$, $\tau^2 = 0.10$, $p = 0.01$					
Test for subgroup differences: $\chi^2_1 = 0.00$, df = 1 ($p = 0.98$)					

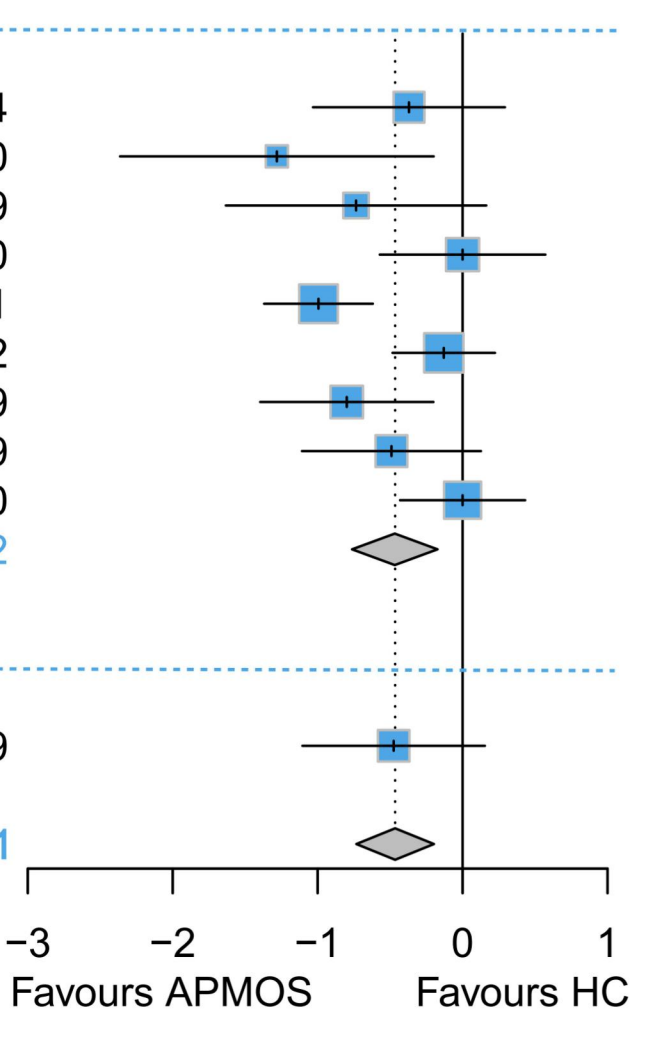

Supplementary Figure 4

(Continued)

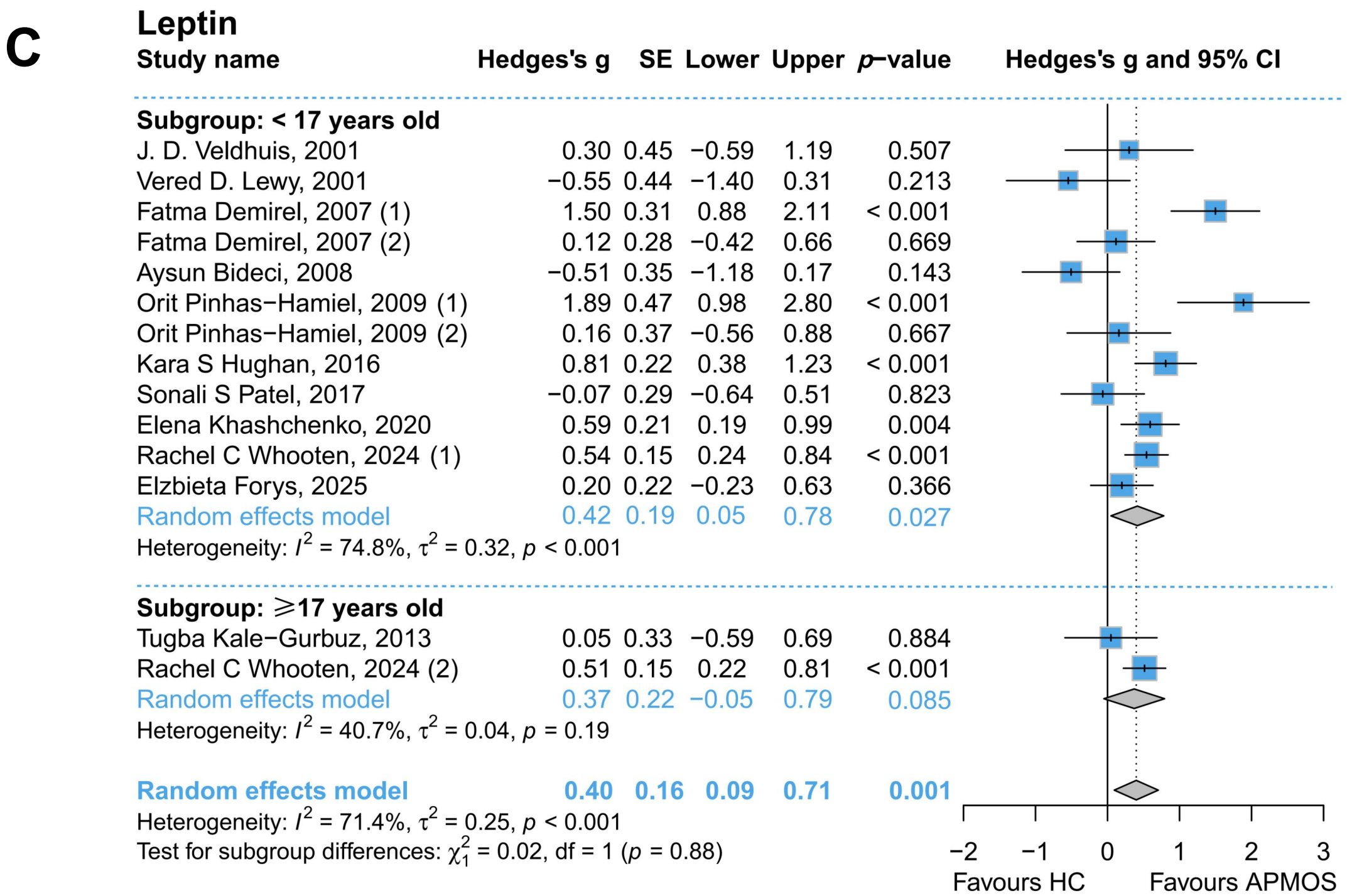

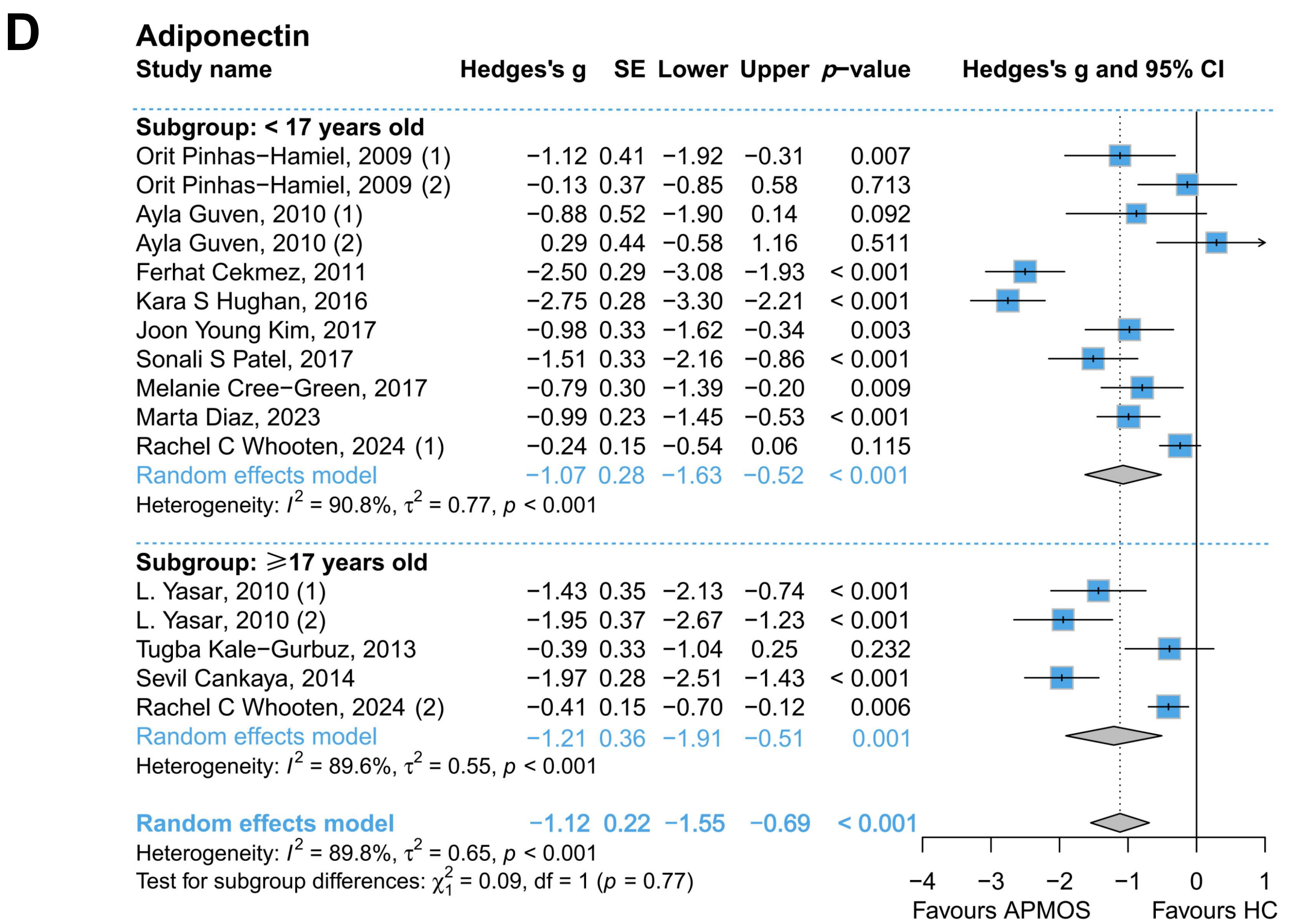

Supplementary figure 4. Effect sizes of the insulin resistance index and adipokines in overall adolescent PMOS and subgroup analysis stratified by age (A-D). Effect size was calculated using Hedges' *g*. Abbreviation: homeostatic model assessment of insulin resistance (HOMA-IR), quantitative insulin sensitivity check index (QUICKI), polyendocrine metabolic ovarian syndrome (PMOS), healthy control (HC), confidence interval (CI).

Supplementary Figure 5

A

HOMA-IR

Study name	Hedges's g	SE	Lower	Upper	p-value
**Subgroup: NIH criteria**					
Fatma Demirel, 2007	0.89	0.29	0.32	1.45	0.002
Franca Fruzzetti, 2009 (1)	0.60	0.21	0.20	1.01	0.004
Franca Fruzzetti, 2009 (2)	0.96	0.24	0.49	1.44	< 0.001
Gideon de Sousa, 2010	0.26	0.29	−0.31	0.83	0.376
Aviva B Sopher, 2012	0.30	0.41	−0.51	1.11	0.463
Haleh Rahmanpour, 2012	0.26	0.22	−0.17	0.69	0.230
Paola Villa, 2012	0.44	0.18	0.08	0.80	0.016
Claudio Villarroel, 2015	0.46	0.24	0.00	0.93	0.049
Kara S Hughan, 2016	1.76	0.24	1.29	2.23	< 0.001
Melanie Cree−Green, 2017	0.52	0.30	−0.07	1.10	0.082
Seda Ates, 2018	0.43	0.21	0.02	0.84	0.042
Donna F Vine, 2020	0.61	0.19	0.23	0.99	0.002
Elvan Bayramoglu, 2021 (1)	−0.06	0.31	−0.66	0.54	0.844
Elvan Bayramoglu, 2021 (2)	0.97	0.36	0.26	1.67	0.007
Merve Gungor, 2022	0.26	0.25	−0.24	0.75	0.312
Karolina Skrzynska, 2024	−0.01	0.23	−0.46	0.43	0.950
Random effects model	0.54	0.11	0.32	0.76	< 0.001
Heterogeneity: I^2 = 67.8%, τ^2 = 0.13, p < 0.001					
**Subgroup: 2 in 3, Rotterdam criteria**					
Aysun Bideci, 2008	0.42	0.34	−0.25	1.10	0.219
Ayla Guven, 2010 (1)	1.26	0.55	0.18	2.34	0.022
Ayla Guven, 2010 (2)	0.39	0.45	−0.49	1.26	0.385
Xiaoli Chen, 2010	0.80	0.33	0.15	1.46	0.016
Ferhat Cekmez, 2011	2.14	0.28	1.60	2.68	< 0.001
Ruya Deveer, 2012	1.51	0.32	0.88	2.15	< 0.001
Tugba Kale−Gurbuz, 2013	−0.17	0.33	−0.81	0.47	0.604
Melissa Pawelczak, 2014	0.10	0.44	−0.76	0.97	0.815
Sevil Cankaya, 2014	1.09	0.24	0.62	1.57	< 0.001
Esra Isci Bostanci, 2018	0.22	0.25	−0.26	0.71	0.368
Ilker Tolga Ozgen, 2019	0.51	0.24	0.03	0.99	0.036
Elzbieta Forys, 2025	0.11	0.22	−0.32	0.54	0.613
Random effects model	0.70	0.20	0.30	1.09	0.001
Heterogeneity: I^2 = 81.3%, τ^2 = 0.38, p < 0.001					
**Subgroup: 3 in 3, Rotterdam criteria**					
Aytekin Tokmak, 2015	0.71	0.22	0.28	1.13	0.001
Aytekin Tokmak, 2018	0.45	0.22	0.03	0.88	0.035
Elif Sagsak, 2021	1.90	0.29	1.33	2.47	< 0.001
Random effects model	1.00	0.44	0.15	1.86	0.022
Heterogeneity: I^2 = 88.2%, τ^2 = 0.51, p < 0.001					
**Random effects model**	**0.64**	**0.10**	**0.44**	**0.84**	**< 0.001**
Heterogeneity: I^2 = 76.5%, τ^2 = 0.24, p < 0.001					
Test for subgroup differences: χ^2_2 = 1.35, df = 2 (p = 0.51)					

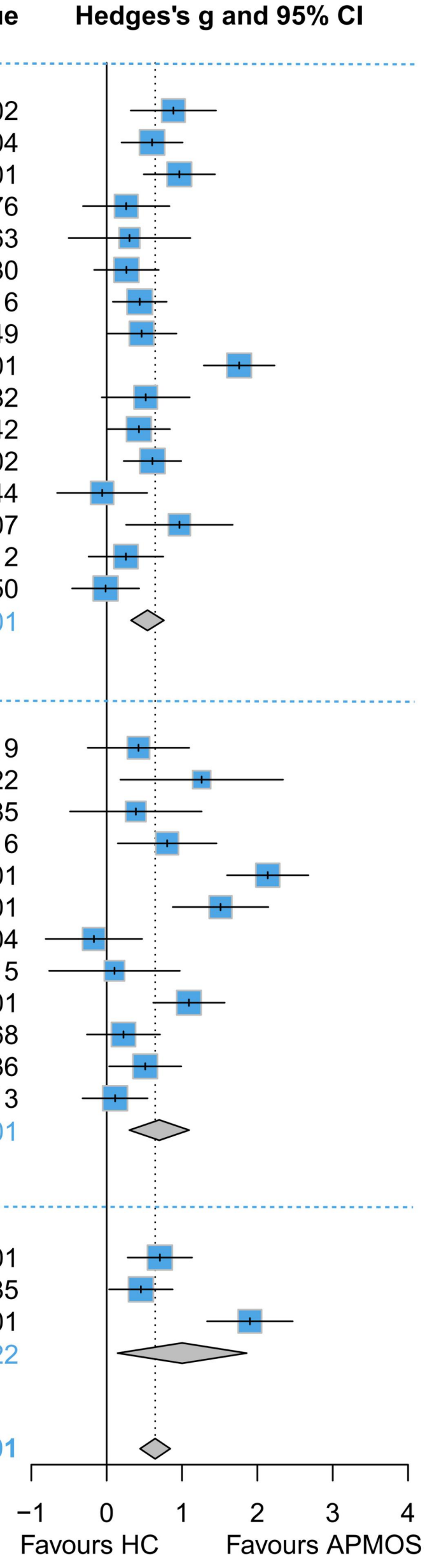

B

QUICKI

Study name	Hedges's g	SE	Lower	Upper	p-value
**Subgroup: NIH**					
Miriam E. Silfen, 2003	−0.37	0.34	−1.03	0.29	0.274
Gideon de Sousa, 2010	0.00	0.29	−0.57	0.57	1.000
Paola Villa, 2012	−0.13	0.18	−0.48	0.22	0.472
Melanie Cree−Green, 2017	−0.80	0.30	−1.40	−0.20	0.009
Random effects model	−0.28	0.16	−0.60	0.04	0.083
Heterogeneity: I^2 = 34%, τ^2 = 0.04, p = 0.21					
**Subgroup: 2 in 3, Rotterdam criteria**					
Ayla Guven, 2010 (1)	−1.28	0.55	−2.36	−0.20	0.020
Ayla Guven, 2010 (2)	−0.73	0.46	−1.63	0.16	0.109
Stefano Angioni, 2010	−0.99	0.19	−1.37	−0.62	< 0.001
Flora Bacopoulou, 2018	−0.49	0.32	−1.11	0.13	0.119
Antigoni Manousopoulou, 2019	−0.48	0.32	−1.10	0.15	0.139
Elzbieta Forys, 2025	0.00	0.22	−0.43	0.43	1.000
Random effects model	−0.60	0.20	−0.98	−0.22	0.002
Heterogeneity: I^2 = 63.1%, τ^2 = 0.12, p = 0.02					
**Random effects model**	**−0.47**	**0.14**	**−0.73**	**−0.20**	**0.001**
Heterogeneity: I^2 = 58.8%, τ^2 = 0.10, p = 0.01					
Test for subgroup differences: χ^2_1 = 1.53, df = 1 (p = 0.22)					

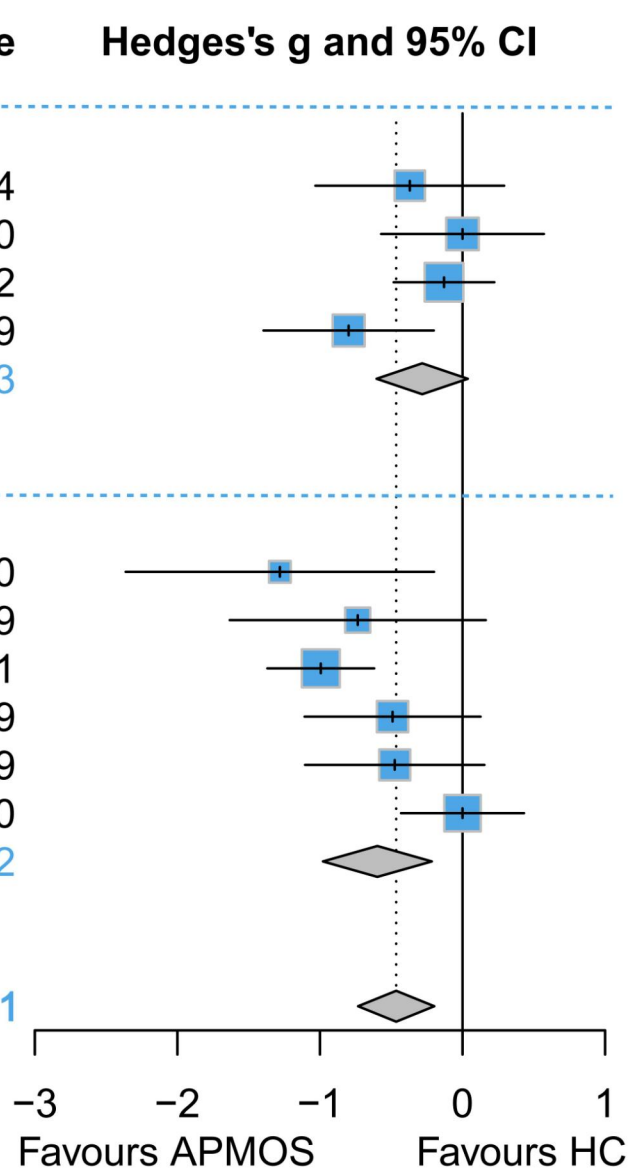

Supplementary Figure 5

(Continued)

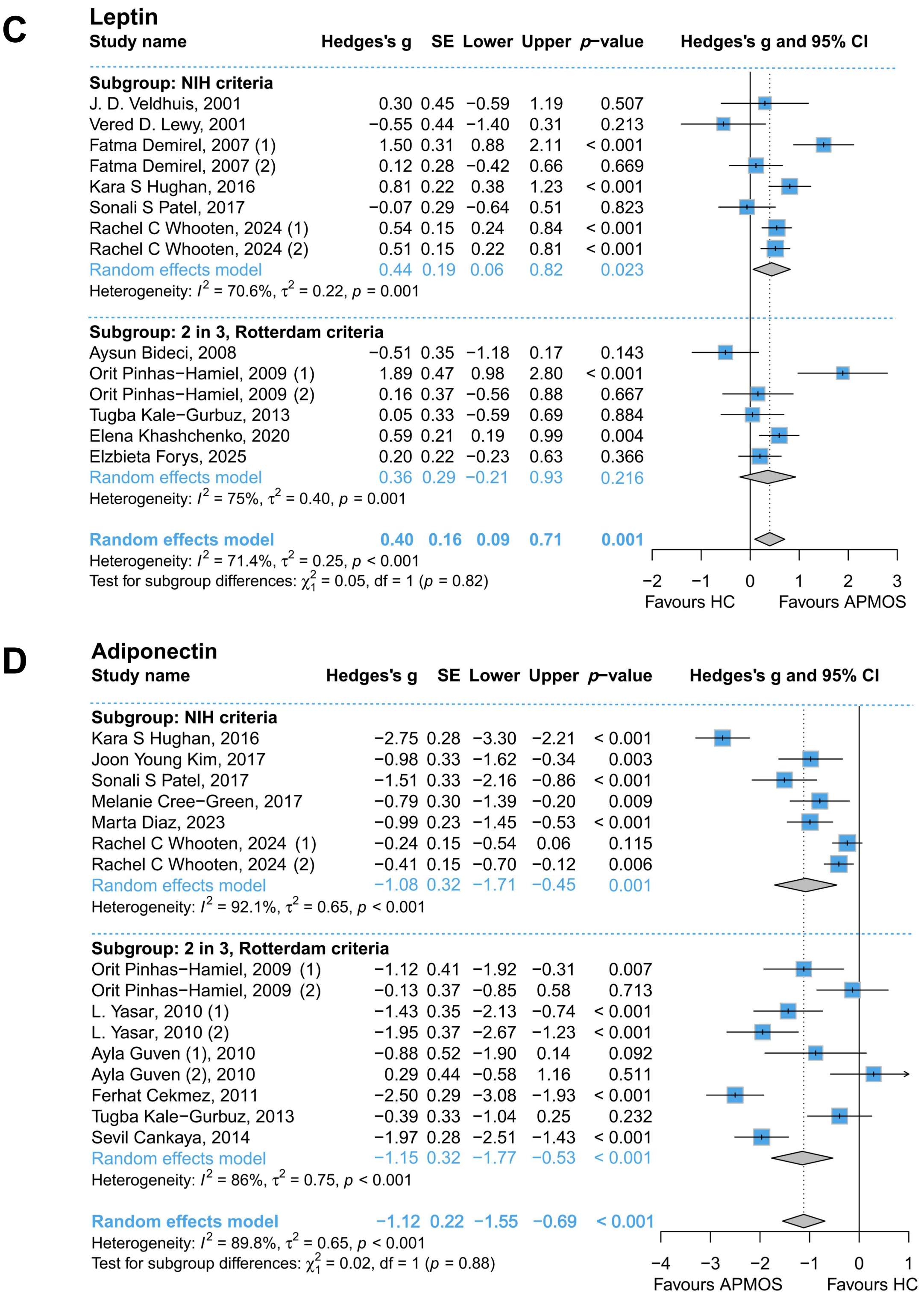

Supplementary figure 5. Effect sizes of the insulin resistance index and adipokines in overall adolescent PMOS and subgroup analysis stratified by diagnostic criteria (A-D). Effect size was calculated using Hedges' *g*. Abbreviation: homeostatic model assessment of insulin resistance (HOMA-IR), quantitative insulin sensitivity check index (QUICKI), polyendocrine metabolic ovarian syndrome (PMOS), healthy control (HC), confidence interval (CI).

Supplementary figure 6

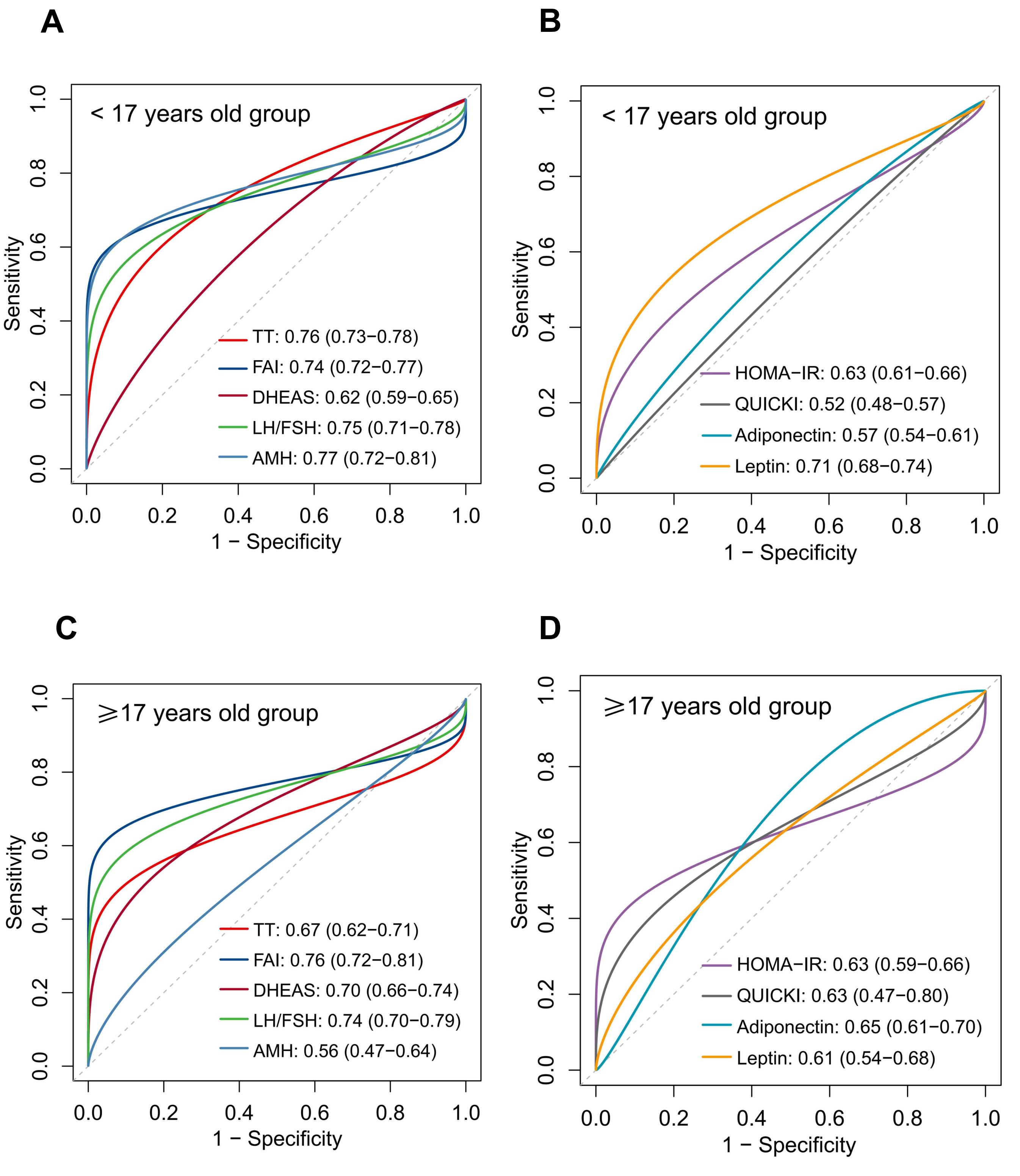

Supplementary figure 6

(Continued)

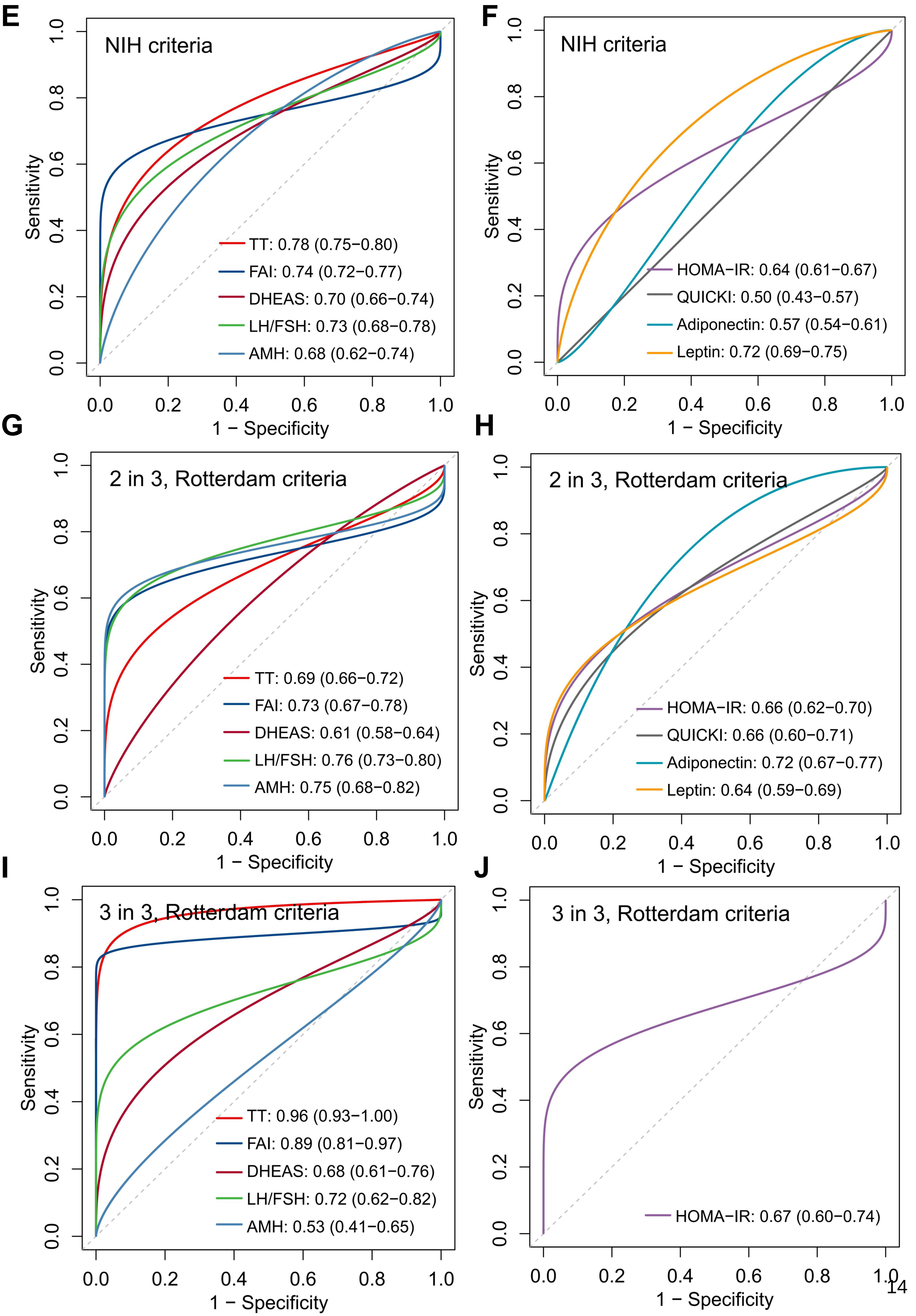

Supplementary figure 6

(Continued)

Supplementary figure 6. Diagnostic performance of the biomarkers in subgroups stratified by age and diagnostic criteria (A-J). Under binormal assumption and moderate heterogeneity, the receiver operating characteristic (ROC) curves were generated by a series of cutoff values. , Area under the receiver operating characteristic curve (AUC) and 95% confidence interval were demonstrated for each biomarker. Abbreviation: total testosterone (TT), free androgen index (FAI), ratio of luteinizing hormone to follicle-stimulating hormone (LH/FSH), dehydroepiandrosterone sulphate (DHEAS), Anti-Müllerian hormone (AMH), homeostatic model assessment of insulin resistance (HOMA-IR), quantitative insulin sensitivity check index (QUICKI).

Supplementary table 1 Searching strategy

Database	Searching engines	Result
PubMed	(“adolescent” OR “teenager” OR “girl”) AND (“polycystic ovary syndrome” OR “PCOS”) AND (“diagnosis” OR “biomarker” OR “free androgen index” OR “FAI” OR “LH/FSH” OR "dehydroepiandrosterone sulfate" OR "DHEA-S" OR “DHEAS” OR “androstenedione” OR “A4” OR "anti-mullerian hormone" OR "AMH" OR "insulin resistance" OR “HOMA-IR” OR “QUICKI”)	2590
Web of Science	(TS=((“adolescent” OR “teenager” OR “girl”) AND (“polycystic ovary syndrome” OR “PCOS”) AND (“diagnosis” OR “biomarker” OR “free androgen index” OR “FAI” OR “LH/FSH” OR "dehydroepiandrosterone sulfate" OR "DHEA-S" OR “DHEAS” OR “androstenedione” OR “A4” OR "anti-mullerian hormone" OR "AMH" OR "insulin resistance" OR “HOMA-IR” OR “QUICKI”))) AND DT=(Article)	3542
Scopus	TITLE-ABS-KEY (("adolescent" OR "teenager" OR "girl") AND ("polycystic ovary syndrome" OR "PCOS") AND ("diagnosis" OR "biomarker" OR "free androgen index" OR "FAI" OR "LH/FSH" OR "dehydroepiandrosterone sulfate" OR "DHEA-S" OR "DHEAS" OR “androstenedione” OR “A4” OR "anti-mullerian hormone" OR "AMH" OR "insulin resistance" OR "HOMA-IR" OR "QUICKI")) AND (LIMIT-TO (DOCTYPE , "ar")) AND (LIMIT-TO (LANGUAGE , "English")) AND (LIMIT-TO (SRCTYPE , "j"))	2213
Embase	(“adolescent” OR “teenager” OR “girl”) AND (“polycystic ovary syndrome” OR “PCOS”) AND (“diagnosis” OR “biomarker” OR “free androgen index” OR “FAI” OR “LH/FSH” OR "dehydroepiandrosterone sulfate" OR "DHEA-S" OR “DHEAS” OR “androstenedione” OR “A4” OR "anti-mullerian hormone" OR "AMH" OR "insulin resistance" OR “HOMA-IR” OR “QUICKI”) AND “article”	2242
Science Direct	(“adolescent” AND “Polycystic Ovary Syndrome” AND “diagnosis” AND “biomarker”) limited to “Research Articles” AND “English”	165
Cochrane Library	(“adolescent” OR “teenager” OR “girl”) AND (“polycystic ovary syndrome” OR “PCOS”) AND (“diagnosis” OR “biomarker”)	164
Total		10926

Supplementary table 2. Studies excluded from meta-analysis after full text screening

Author	Region	Year	Biomarker	Reason for exclusion
Sevgi Yardım Akaydın	Turkey	2024	Adipsin	not targeted biomarker
I.T. Ozgen	Turkey	2010	LH/FSH, DHEAS, HOMA-IR	using rare criteria
Sesil Erolova Kedikova	Bulgaria	2013	Leptin, LH/FSH, HOMA-IR	using rare criteria
Ismail Guzelkas	Turkey	2022	Leptin, LH/FSH	unspecified criteria
Lin Li	China	2010	AMH	population mean age > 19 years old
Ülker Gümüş	Turkey	2015	DHEAS, HOMA-IR, Visfatin	population mean age > 19 years old
Franca Fruzzetti	Italy	2016	DHEAS, FAI, HOMA-IR	population mean age > 19 years old
Agnieszka Białka-Kosiec1	Poland	2022	Leptin, AMH, HOMA-IR	population mean age > 19 years old

Abbreviation: ratio of luteinizing hormone to follicle-stimulating hormone (LH/FSH), dehydroepiandrosterone sulphate (DHEAS), antimullerian hormone (AMH), homeostatic model assessment of insulin resistance (HOMA-IR).

Supplementary table 3. Quality assessment of the included studies

Author	Year	Source of information	Inclusion and exclusion criteria	Time period for identifying patients	Consecutive subjects (if not population-based)	Mask subjective components	Assessments for quality assurance	Patient exclusions from analysis	Confounding assessment and/or control	Missing data handling	Response rates and completeness	Clarify follow-up	Overall quality
J. D. Veldhuis	2001	+	+	+	+	+	+	○	○	○	+	○	moderate
Vered D. Lewy	2001	+	+	+	+	+	+	○	○	○	+	○	moderate
Miriam E. Silfen	2003	+	+	+	+	+	+	○	○	○	+	○	moderate
Fatma Demirel	2007	+	+	+	+	+	+	○	+	○	+	○	high
Aysun Bideci	2008	+	+	+	+	+	+	○	○	○	+	○	moderate
Brooke Rossi	2008	+	+	+	+	+	+	○	○	○	+	○	moderate
Franca Fruzzetti	2009	+	+	+	+	+	+	○	+	○	+	○	high
Orit Pinhas-Hamiel	2009	+	+	+	+	+	+	○	○	○	+	○	moderate
Ayla Güven	2010	+	+	+	+	+	+	○	○	○	+	○	moderate
Gideon de Sousa	2010	+	+	+	+	+	+	○	○	○	+	○	moderate
L. Yasar	2010	+	+	+	+	+	+	○	+	○	+	○	moderate
Stefano Angioni	2010	+	+	+	+	+	+	○	○	○	+	○	moderate

(Continued)

Xiaoli Chen	2010	+	+	+	+	+	+	○	+	○	+	○	high
Ferhat Cekmez	2011	+	+	+	+	+	+	○	○	○	+	○	moderate
Aviva B Sopher	2012	+	+	+	+	+	+	○	○	○	+	○	moderate
Haleh Rahmanpour	2012	+	+	+	+	+	+	○	○	○	+	○	moderate
Paola Villa	2012	+	+	+	+	+	+	○	○	○	+	○	moderate
Rüya Deveer	2012	+	+	+	+	+	+	○	○	○	+	○	moderate
Andrea Hsu Roe	2013	+	+	+	+	+	+	○	○	○	+	○	moderate
Tugba Kale-Gurbuz	2013	+	+	+	+	+	+	○	○	○	+	○	moderate
Aviva B Sopher	2014	+	+	+	+	+	+	○	+	○	+	○	high
Melissa Pawelczak	2014	+	+	+	+	+	+	○	+	○	+	○	high
Sevil Cankaya	2014	+	+	+	+	+	+	○	○	○	+	○	moderate
Aytekin Tokmak	2015	+	+	+	+	+	+	○	+	○	+	○	high
Claudio Villarroel	2015	+	+	+	+	+	+	○	○	○	+	○	moderate
Aylin Yetim	2016	+	+	+	+	+	+	○	+	○	+	○	high
Kara S Hughan	2016	+	+	+	+	+	+	○	○	○	+	○	moderate
Joon Young Kim	2017	+	+	+	+	+	+	○	○	○	+	○	moderate

(Continued)

Melanie Cree-Green	2017	+	+	+	+	+	+	○	○	○	+	○	moderate
Sonali S Patel	2017	+	+	+	+	+	+	○	○	○	+	○	moderate
Aytekin Tokmak	2018	+	+	+	+	+	+	○	○	○	+	○	moderate
Esra Isci Bostanci	2018	+	+	+	+	+	+	○	+	○	+	○	high
Flora Bacopoulou	2018	+	+	+	+	+	+	○	○	○	+	○	moderate
Seda Ates	2018	+	+	+	+	+	+	○	+	○	+	○	high
Antigoni Manousopoulou	2019	+	+	+	+	+	+	○	○	○	+	○	moderate
İlker Tolga Özgen	2019	+	+	+	+	+	+	○	○	○	+	○	moderate
Donna F Vine	2020	+	+	+	+	+	+	○	○	○	+	○	moderate
Elena Khashchenko	2020	+	+	+	+	+	+	○	○	○	+	○	moderate
Agnieszka Zachurzok	2021	+	+	+	+	+	+	○	○	○	+	○	moderate
Elif Sağsak	2021	+	+	+	+	+	+	○	○	○	+	○	moderate
Elvan Bayramoğlu	2021	+	+	+	+	+	+	○	+	○	+	○	high
Selma Tunç	2021	+	+	+	+	+	+	○	+	○	+	○	high
Shivi Jain	2021	+	+	+	+	+	+	○	○	○	+	○	moderate
Merve Gungor	2022	+	+	+	+	+	+	○	○	○	+	○	moderate

(Continued)

Giannouli, A	2023	+	+	+	+	+	+	○	+	○	+	○	high
Gönül Büyükyılmaz	2023	+	+	+	+	+	+	○	+	○	+	○	high
Marta Díaz	2023	+	+	+	+	+	+	○	○	○	+	○	moderate
Karolina Skrzyńska	2024	+	+	+	+	+	+	○	+	○	+	○	high
Rachel C Whooten	2024	+	+	+	+	+	+	○	+	○	+	○	high
Leyla Kara	2025	+	+	+	+	+	+	○	○	○	+	○	moderate
Elzbieta Forys	2025	+	+	+	+	+	+	○	○	○	+	○	moderate

Annotation: “-” represents “high risk”, “○” represents “unclear risk”, and “+” represents “low risk”.

Supplementary table 4. Sensitivity analysis by omitting single study

Biomarker	Omitted study	Study quality	Hedges'g	Lower 95% CI	Upper 95% CI	*p*-value
TT	Vered D. Lewy, 2001	moderate	1.07	0.84	1.30	< 0.001
	Miriam E. Silfen, 2003	moderate	1.08	0.84	1.31	< 0.001
	Brooke Rossi, 2008	moderate	1.12	0.87	1.36	< 0.001
	Franca Fruzzetti (1), 2009	high	1.11	0.86	1.36	< 0.001
	Franca Fruzzetti (2), 2009	high	1.09	0.84	1.33	< 0.001
	Orit Pinhas-Hamiel (1), 2009	moderate	1.11	0.87	1.35	< 0.001
	Orit Pinhas-Hamiel (2), 2009	moderate	1.12	0.87	1.36	< 0.001
	Xiaoli Chen, 2010	high	1.12	0.88	1.36	< 0.001
	Ayla Guven (2), 2010	moderate	1.13	0.89	1.37	< 0.001
	Stefano Angioni, 2010	moderate	1.12	0.87	1.36	< 0.001
	Gideon de Sousa, 2010	moderate	1.12	0.88	1.36	< 0.001
	Ferhat Cekmez, 2011	moderate	1.09	0.85	1.34	< 0.001
	Paola Villa, 2012	moderate	1.06	0.83	1.29	< 0.001
	Aviva B Sopher, 2012	moderate	1.12	0.87	1.36	< 0.001
	Tugba Kale-Gurbuz, 2013	moderate	1.13	0.88	1.37	< 0.001
	Aviva B Sopher, 2014	high	1.12	0.87	1.36	< 0.001
	Sevil Cankaya, 2014	moderate	1.12	0.88	1.37	< 0.001
	Melanie Cree-Green, 2017	moderate	1.12	0.87	1.36	< 0.001
	Sonali S Patel, 2017	moderate	1.12	0.87	1.36	< 0.001
	Seda Ates, 2018	high	1.14	0.91	1.37	< 0.001
	Flora Bacopoulou, 2018	moderate	1.08	0.84	1.32	< 0.001
	Antigoni Manousopoulou, 2019	moderate	1.08	0.84	1.32	< 0.001
	Ilker Tolga Ozgen, 2019	moderate	1.11	0.87	1.36	< 0.001
	Donna F Vine, 2020	moderate	1.10	0.85	1.34	< 0.001
	Elena Khashchenko, 2020	moderate	1.10	0.86	1.35	< 0.001
	Agnieszka Zachurzok, 2021	moderate	1.11	0.87	1.36	< 0.001
	Elif Sagsak, 2021	moderate	1.04	0.83	1.24	< 0.001
	Gonul Buyukyilmaz, 2023	high	1.09	0.84	1.33	< 0.001
	Giannouli, A, 2023	high	1.13	0.89	1.37	< 0.001
	Karolina Skrzynska, 2024	high	1.09	0.84	1.33	< 0.001

(Continued)

	Brooke Rossi, 2008	moderate	1.22	0.94	1.49	< 0.001
	Franca Fruzzetti, 2009	high	1.22	0.94	1.50	< 0.001
	Franca Fruzzetti, 2009	high	1.17	0.89	1.45	< 0.001
	Xiaoli Chen, 2010	high	1.23	0.96	1.49	< 0.001
	Ferhat Cekmez, 2011	moderate	1.20	0.92	1.48	< 0.001
	Paola Villa, 2012	moderate	1.18	0.90	1.46	< 0.001
	Melanie Cree Green, 2017	moderate	1.20	0.93	1.48	< 0.001
	Sonali S Patel, 2017	moderate	1.18	0.91	1.46	< 0.001
FAI	Seda Ates, 2018	high	1.24	0.98	1.50	< 0.001
	Antigoni Manousopoulou, 2019	moderate	1.18	0.90	1.46	< 0.001
	Donna F Vine, 2020	moderate	1.13	0.88	1.38	< 0.001
	Elif Sagsak, 2021	moderate	1.10	0.88	1.32	< 0.001
	Merve Gungor, 2022	moderate	1.21	0.93	1.49	< 0.001
	Gonul Buyukyilmaz, 2023	high	1.17	0.90	1.45	< 0.001
	Gonul Buyukyilmaz, 2023	high	1.17	0.89	1.45	< 0.001
	Leyla Kara, 2025	moderate	1.21	0.93	1.49	< 0.001
	J. D. Veldhuis, 2001	moderate	0.60	0.44	0.77	< 0.001
	Miriam E. Silfen, 2003	moderate	0.63	0.46	0.81	< 0.001
	Fatma Demirel, 2007	high	0.60	0.44	0.76	< 0.001
	Aysun Bideci, 2008	moderate	0.62	0.45	0.79	< 0.001
	Orit Pinhas-Hamiel, 2009	moderate	0.64	0.46	0.81	< 0.001
	Orit Pinhas-Hamiel, 2009	moderate	0.64	0.46	0.81	< 0.001
	Ayla Guven, 2010	moderate	0.63	0.46	0.81	< 0.001
	Ayla Guven, 2010	moderate	0.62	0.45	0.80	< 0.001
DHEAS	Stefano Angioni, 2010	moderate	0.67	0.51	0.83	< 0.001
	Aviva B Sopher, 2012	moderate	0.64	0.46	0.81	< 0.001
	Tugba Kale-Gurbuz, 2013	moderate	0.65	0.48	0.83	< 0.001
	Andrea Hsu Roe, 2013	moderate	0.66	0.49	0.83	< 0.001
	Aviva B Sopher, 2014	high	0.64	0.46	0.81	< 0.001
	Sevil Cankaya, 2014	moderate	0.65	0.47	0.83	< 0.001
	Claudio Villarroel, 2015	moderate	0.64	0.46	0.82	< 0.001
	Aytekin Tokmak, 2015	high	0.64	0.46	0.82	< 0.001
	Aylin Yetim, 2016	high	0.64	0.46	0.82	< 0.001

(Continued)

	Flora Bacopoulou, 2018	moderate	0.63	0.46	0.81	< 0.001
	Aytekin Tokmak, 2018	moderate	0.63	0.46	0.81	< 0.001
	Antigoni Manousopoulou, 2019	moderate	0.62	0.45	0.79	< 0.001
	Elena Khashchenko, 2020	moderate	0.64	0.46	0.82	< 0.001
	Donna F Vine, 2020	moderate	0.64	0.46	0.82	< 0.001
	Selma Tunc, 2021	high	0.58	0.43	0.74	< 0.001
	Agnieszka Zachurzok, 2021	moderate	0.65	0.47	0.83	< 0.001
	Merve Gungor, 2022	moderate	0.66	0.48	0.83	< 0.001
	Giannouli. A, 2023	high	0.66	0.48	0.83	< 0.001
	Giannouli.A, 2023	high	0.66	0.49	0.83	< 0.001
	Karolina Skrzynska, 2024	high	0.64	0.46	0.81	< 0.001
	Leyla Kara, 2025	moderate	0.65	0.47	0.83	< 0.001
LH/FSH	Fatma Demirel, 2007	high	1.08	0.87	1.28	< 0.001
	Aysun Bideci, 2008	moderate	1.03	0.85	1.21	< 0.001
	Ayla Guven, 2010	moderate	1.07	0.87	1.26	< 0.001
	Ayla Guven, 2010	moderate	1.04	0.86	1.23	< 0.001
	Stefano Angioni, 2010	moderate	1.12	0.92	1.31	< 0.001
	Tugba Kale-Gurbuz, 2013	moderate	1.08	0.88	1.28	< 0.001
	Claudio Villarroel, 2015	moderate	1.08	0.87	1.29	< 0.001
	Aytekin Tokmak, 2015	high	1.11	0.90	1.32	< 0.001
	Esra Isci Bostanci, 2018	high	1.00	0.83	1.17	< 0.001
	Seda Ates, 2018	high	1.12	0.92	1.31	< 0.001
	Selma Tunc, 2021	high	1.09	0.89	1.30	< 0.001
	Shivi Jain, 2021	moderate	1.11	0.90	1.32	< 0.001
	Gonul Buyukyilmaz, 2023	high	1.08	0.87	1.28	< 0.001
	Gonul Buyukyilmaz, 2023	high	1.05	0.85	1.25	< 0.001
AMH	Orit Pinhas-Hamiel (1), 2009	moderate	0.97	0.44	1.51	< 0.001
	Orit Pinhas-Hamiel (2), 2009	moderate	0.98	0.44	1.51	< 0.001
	Aviva B. Sopher, 2014	high	1.01	0.48	1.55	< 0.001
	Claudio Villarroel, 2015	moderate	1.11	0.63	1.58	< 0.001
	Aytekin Tokmak, 2015	high	1.10	0.62	1.59	< 0.001
	Melanie Cree-Green, 2017	moderate	0.96	0.42	1.50	< 0.001
	Selma Tunc, 2021	high	1.00	0.46	1.55	< 0.001

(Continued)

	Gonul Buyukyilmaz (1), 2023	high	0.91	0.39	1.43	0.001
	Gonul Buyukyilmaz (2), 2023	high	0.80	0.40	1.19	< 0.001
	Fatma Demirel, 2007	high	0.64	0.43	0.84	< 0.001
	Aysun Bideci, 2008	moderate	0.65	0.44	0.86	< 0.001
	Franca Fruzzetti(1), 2009	high	0.65	0.44	0.85	< 0.001
	Franca Fruzzetti(2), 2009	high	0.63	0.43	0.84	< 0.001
	Ayla Guven(1), 2010	moderate	0.63	0.43	0.84	< 0.001
	Ayla Guven (2), 2010	moderate	0.65	0.44	0.86	< 0.001
	Xiaoli Chen, 2010	high	0.64	0.43	0.85	< 0.001
	Gideon de Sousa, 2010	moderate	0.66	0.45	0.86	< 0.001
	Ferhat Cekmez, 2011	moderate	0.59	0.41	0.77	< 0.001
	Aviva B Sopher, 2012	moderate	0.65	0.45	0.86	< 0.001
	Haleh Rahmanpour, 2012	moderate	0.66	0.45	0.86	< 0.001
	Paola Villa, 2012	moderate	0.65	0.44	0.86	< 0.001
	Ruya Deveer, 2012	moderate	0.62	0.42	0.82	< 0.001
	Tugba Kale-Gurbuz, 2013	moderate	0.67	0.47	0.87	< 0.001
	Melissa Pawelczak, 2014	high	0.66	0.45	0.86	< 0.001
HOMA-IR	Sevil Cankaya, 2014	moderate	0.63	0.42	0.83	< 0.001
	Claudio Villarroel, 2015	moderate	0.65	0.44	0.86	< 0.001
	Aytekin Tokmak, 2015	high	0.64	0.43	0.85	< 0.001
	Kara S Hughan, 2016	moderate	0.60	0.41	0.79	< 0.001
	Melanie Cree-Green, 2017	moderate	0.65	0.44	0.86	< 0.001
	Esra Isci Bostanci, 2018	high	0.66	0.45	0.86	< 0.001
	Aytekin Tokmak, 2018	moderate	0.65	0.44	0.86	< 0.001
	Seda Ates, 2018	high	0.65	0.44	0.86	< 0.001
	Ilker Tolga Ozgen, 2019	moderate	0.65	0.44	0.86	< 0.001
	Donna F Vine, 2020	moderate	0.65	0.44	0.85	< 0.001
	Elif Sagsak, 2021	moderate	0.60	0.41	0.79	< 0.001
	Elvan Bayramoglu (1), 2021	high	0.67	0.46	0.87	< 0.001
	Elvan Bayramoglu (2), 2021	high	0.63	0.43	0.84	< 0.001
	Merve Gungor, 2022	moderate	0.66	0.45	0.86	< 0.001
	Karolina Skrzynska, 2024	high	0.67	0.47	0.87	< 0.001
	Elzbieta Forys, 2025	moderate	0.66	0.46	0.87	< 0.001

(Continued)

QUICKI	Miriam E. Silfen, 2003	moderate	-0.48	-0.77	-0.19	0.001
	Ayla Guven (1), 2010	moderate	-0.43	-0.69	-0.16	0.002
	Ayla Guven (2), 2010	moderate	-0.45	-0.73	-0.17	0.002
	Gideon de Sousa, 2010	moderate	-0.52	-0.80	-0.24	< 0.001
	Stefano Angioni, 2010	moderate	-0.34	-0.57	-0.11	0.003
	Paola Villa, 2012	moderate	-0.52	-0.81	-0.23	< 0.001
	Melanie Cree-Green, 2017	moderate	-0.43	-0.72	-0.14	0.003
	Flora Bacopoulou, 2018	moderate	-0.47	-0.76	-0.17	0.002
	Antigoni Manousopoulou, 2019	moderate	-0.47	-0.76	-0.17	0.002
	Elzbieta Forys, 2025	moderate	-0.53	-0.81	-0.26	< 0.001
Adiponectin	Orit Pinhas-Hamiel (1), 2009	moderate	-1.12	-1.57	-0.66	< 0.001
	Orit Pinhas-Hamiel (2), 2009	moderate	-1.18	-1.62	-0.74	< 0.001
	L. Yasar (1), 2010	moderate	-1.10	-1.55	-0.64	< 0.001
	L. Yasar (2), 2010	moderate	-1.06	-1.51	-0.62	< 0.001
	Ayla Guven (1), 2010	moderate	-1.13	-1.58	-0.68	< 0.001
	Ayla Guven (2), 2010	moderate	-1.20	-1.62	-0.78	< 0.001
	Ferhat Cekmez, 2011	moderate	-1.02	-1.44	-0.61	< 0.001
	Tugba Kale-Gurbuz, 2013	moderate	-1.17	-1.61	-0.72	< 0.001
	Sevil Cankaya, 2014	moderate	-1.06	-1.50	-0.62	< 0.001
	Kara S Hughan, 2016	moderate	-1.00	-1.40	-0.61	< 0.001
	Joon Young Kim, 2017	moderate	-1.13	-1.58	-0.67	< 0.001
	Sonali S Patel, 2017	moderate	-1.09	-1.54	-0.64	< 0.001
	Melanie Cree-Green, 2017	moderate	-1.14	-1.59	-0.68	< 0.001
	Marta Diaz, 2023	moderate	-1.13	-1.58	-0.67	< 0.001
	Rachel C Whooten (1), 2024	high	-1.18	-1.62	-0.75	< 0.001
	Rachel C Whooten (2), 2024	high	-1.17	-1.62	-0.72	< 0.001
Leptin	J. D. Veldhuis, 2001	moderate	0.40	0.08	0.73	0.015
	Vered D. Lewy, 2001	moderate	0.45	0.15	0.75	0.003
	Fatma Demirel (1), 2007	high	0.32	0.06	0.59	0.016
	Fatma Demirel (2), 2007	high	0.42	0.09	0.75	0.013
	Aysun Bideci, 2008	moderate	0.46	0.17	0.76	0.002
	Orit Pinhas-Hamiel (1), 2009	moderate	0.33	0.06	0.60	0.017
	Orit Pinhas-Hamiel (2), 2009	moderate	0.42	0.09	0.74	0.013

(Continued)

	Tugba Kale-Gurbuz, 2013	moderate	0.42	0.10	0.75	0.011
	Kara S Hughan, 2016	moderate	0.36	0.04	0.69	0.03
	Sonali S Patel, 2017	moderate	0.44	0.11	0.76	0.008
	Elena Khashchenko, 2020	moderate	0.38	0.05	0.72	0.026
	Rachel C Whooten (1), 2024	high	0.39	0.05	0.72	0.026
	Rachel C Whooten (2), 2024	high	0.39	0.05	0.73	0.026
	Elzbieta Forys, 2025	moderate	0.42	0.08	0.75	0.015

Annotation: Leave-one-out method was applied for sensesitivity analysis. The table demonstrated the effect size (Hedges' g with 95% confidence interval), and the *p*-value of the effect size after omitting the study.

Abbreviation: total testosterone (TT), free androgen index (FAI), ratio of luteinizing hormone to follicle-stimulating hormone (LH/FSH), dehydroepiandrosterone sulphate (DHEAS), antimullerian hormone (AMH), homeostatic model assessment of insulin resistance (HOMA-IR), quantitative insulin sensitivity check index (QUICKI), confidence interval (CI).

Supplementary table 5. Sensitivity analysis by omitting studies in moderate quality

Biomarker	Quality	Studies	Hedges'g	SE	Lower 95% CI	Upper 95% CI	*P*-value	I^2	Tau^2
TT	High	8	0.94	0.21	0.53	1.35	< 0.001	0.83	0.28
	Overall	30	1.10	0.12	0.87	1.34	< 0.001	0.80	0.34
FAI	High	6	1.01	0.2	0.62	1.40	< 0.001	0.72	0.17
	Overall	16	1.19	0.13	0.93	1.45	< 0.001	0.76	0.22
DHEAS	High	8	0.74	0.21	0.33	1.15	< 0.001	0.77	0.27
	Overall	29	0.64	0.09	0.47	0.81	< 0.001	0.66	0.13
LH/FSH	High	6	1.13	0.16	0.82	1.4	< 0.001	0.51	0.07
	Overall	14	1.07	0.10	0.88	1.27	< 0.001	0.41	0.05
AMH	High	4	1.39	0.37	0.67	2.11	< 0.001	0.81	0.44
	Overall	9	0.98	0.24	0.50	1.46	< 0.001	0.84	0.44
HOMA-IR	High	11	0.51	0.11	0.29	0.73	< 0.001	0.49	0.07
	Overall	31	0.64	0.1	0.44	0.84	< 0.001	0.77	0.24
QUICKI	High	0	NA	NA	NA	NA	NA	NA	NA
	Overall	10	-0.47	0.14	-0.73	-0.2	0.001	0.59	0.10
Adiponectin	High	2	-0.33	0.11	-0.54	-0.12	0.002	< 0.001	< 0.001
	Overall	16	-1.12	0.22	-1.55	-0.69	< 0.001	0.90	0.65
Leptin	High	4	0.64	0.26	0.14	1.15	0.013	0.74	0.21
	Overall	14	0.4	0.16	0.09	0.71	0.01	0.71	0.25

Annotation: Studies in moderate quality were omitted for sensesitivity analysis. The table demonstrated the effect size (Hedges' g with 95% confidence interval), and the *p*-value of the effect size after omitting moderate-quality studies.

Abbreviation: total testosterone (TT), free androgen index (FAI), ratio of luteinizing hormone to follicle-stimulating hormone (LH/FSH), dehydroepiandrosterone sulphate (DHEAS), antimullerian hormone (AMH), homeostatic model assessment of insulin resistance (HOMA-IR), quantitative insulin sensitivity check index (QUICKI), standard error (SE), confidence interval (CI).

Supplementary table 6. Evidence certainty (GRADE) of the biomarkers

	Certainty assessment							Summary of findings	
**Biomarker**	**Studies**	**Risk of bias**	**Inconsistency**	**Indirectness**	**Imprecision**	**Publication bias**	**Overall certainty of evidence**	**Study event**	**Effect size (95% CI)**
TT	30	not serious	not serious	not serious	not serious	none	**Moderate**	1196 cases 969 controls	1.10 (0.87 to 1.34)
FAI	15	not serious	not serious	not serious	not serious	none	**Moderate**	558 cases 898 controls	1.19 (0.93 to 1.45)
DHEAS	28	not serious	not serious	not serious	not serious	strongly suspected	**Very low**	1081 cases 961 controls	0.64 (0.47 to 0.81)
LH/FSH	14	not serious	not serious	not serious	not serious	strongly suspected	**Low**	495 cases 460 controls	1.07 (0.88 to 1.27)
AMH	9	not serious	not serious	not serious	not serious	none	**Moderate**	253 cases 269 controls	0.98 (0.50 to 1.46)
HOMA-IR	31	not serious	not serious	not serious	not serious	none	**Low**	1182 cases 1083 controls	0.64 (0.44 to 0.84)
QUICKI	10	not serious	not serious	not serious	not serious	none	**Low**	377 cases 237 controls	-0.47 (-0.73 to -0.20)
Adiponectin	16	not serious	not serious	not serious	not serious	none	**Moderate**	550 cases 944 controls	-1.12 (-1.55 to -0.69)
Leptin	14	not serious	not serious	not serious	not serious	none	**Low**	558 cases 898 controls	0.40 (0.09 to 0.71)

Abbreviation: total testosterone (TT), free androgen index (FAI), ratio of luteinizing hormone to follicle-stimulating hormone (LH/FSH), dehydroepiandrosterone sulphate (DHEAS), antimullerian hormone (AMH), homeostatic model assessment of insulin resistance (HOMA-IR), quantitative insulin sensitivity check index (QUICKI), confidence interval (CI).

Supplementary table 7. Stratified meta-regression analysis within subgroup

Biomarker	Subgroup	Covariate	Study numbers	QM statistic	*p*-value	R^2 (%)
TT	Non-obese	Age	21	0.36	0.55	0
		Criteria	21	12.3	< 0.001	40.39
	< 17 years old	BMI	23	0	1	0
		Criteria	23	13.26	< 0.001	45.03
	NIH criteria	BMI	16	0.07	0.79	0
		Age	16	0.29	0.59	0
	2 in 3, Rotterdam criteria	BMI	13	0.07	0.79	0
		Age	13	0.22	0.64	0
FAI	Non-obese	Age	12	0.05	0.83	0
		Criteria	12	8.26	0.02	42.69
	< 17 years old	BMI	11	0.14	0.71	0
		Criteria	11	21.03	< 0.001	93.9
	NIH criteria	BMI	11	0	0.98	0
		Age	11	0.4	0.53	0
DHEAS	Non-obese	Age	23	0	0.96	0
		Criteria	23	0.7	0.7	0
	< 17 years old	BMI	22	0.01	0.93	0
		Criteria	22	1.19	0.28	0
	NIH criteria	BMI	10	0.02	0.88	0
		Age	10	0.45	0.5	0
	2 in 3, Rotterdam criteria	BMI	17	0.01	0.92	0
		Age	17	0	0.97	0
LH/FSH	Non-obese	Age	10	0.01	0.94	0
		Criteria	10	0.37	0.83	0
HOMA-IR	Non-obese	Age	22	0.27	0.6	0
		Criteria	22	3.03	0.22	4.35
	< 17 years old	BMI	19	0.58	0.45	0
		Criteria	19	4.42	0.11	14.7
	≥ 17 years old	BMI	12	3.75	0.05	30.46

(Continued)

		Criteria	12	0.3	0.86	0
	NIH criteria	BMI	16	0.5	0.48	0
		Age	16	0	1	0
	2 in 3, Rotterdam criteria	BMI	12	0.01	0.94	0
		Age	12	0	0.98	0
Adiponectin	< 17 years old	BMI	11	9.35	< 0.001	52.31
		Criteria	11	0.26	0.61	0
Leptin	< 17 years old	BMI	12	0.2	0.65	0
		Criteria	12	0	0.99	0

Annotation: Stratified meta-regression analyses were performed using random-effects models within each subgroup (inclued study number ≥10 to ensure adequate statistical power for stable estimation of subgroup-specific random-effects variance and reliable detection of potential effect modifiers) to evaluate the influence of covariates (age, BMI, diagnostic criteria) on the pooled effect size of the biomarkers. The QM statistic tests the joint significance of the regression coefficient(s) for the covariate. *P*-value indicates the corresponding significance level. R^2 (%) quantifies the proportion of between-study heterogeneity explained by the covariate.

Abbreviation: total testosterone (TT), free androgen index (FAI), ratio of luteinizing hormone to follicle-stimulating hormone (LH/FSH), dehydroepiandrosterone sulphate (DHEAS), antimullerian hormone (AMH), homeostatic model assessment of insulin resistance (HOMA-IR), quantitative insulin sensitivity check index (QUICKI).
